# Thiazole–driven heterocyclic hybrids as EGFR inhibitors: progress in synthesis and anticancer applications

**DOI:** 10.1039/d5ra04838a

**Published:** 2026-01-02

**Authors:** Ancilla Dsouza, V. M. Subrahmanyam, Nitinkumar S. Shetty

**Affiliations:** a Department of Chemistry, Manipal Institute of Technology, Manipal Academy of Higher Education Manipal Karnataka 576104 India nitin.shetty@manipal.edu; b Department of Pharmaceutical Biotechnology, Manipal College of Pharmaceutical Sciences, Manipal Academy of Higher Education Manipal Karnataka 576104 India

## Abstract

Despite considerable progress in therapeutic developments, cancer still stands as one of the most common and lethal health challenges worldwide. Targeting the EGFR signaling pathway has emerged as a key approach in cancer therapy. Inhibiting the intracellular tyrosine kinase domain of EGFR has demonstrated significant therapeutic benefits. To discover effective EGFR tyrosine kinase inhibitors (TKIs), numerous small molecules, particularly thiazole-based hybrids have been developed using molecular modelling techniques. However, challenges such as epigenetic mutations and acquired resistance have limited their long-term efficacy, highlighting the need for continued research in this area. Recent efforts have focused on understanding genetic alterations within the EGFR tyrosine kinase domain, paving the way for the development of more selective and potent inhibitors. This review presents an overview of the role and current landscape of EGFR inhibitors in cancer treatment, with a particular emphasis on recent progress in the design, synthesis, and development of novel thiazole hybrid compounds as promising selective EGFR TKIs.

## Abstract 2

An overview of the role and current landscape of EGFR inhibitors in cancer treatment, with a particular emphasis on recent progress in the design, synthesis, and development of novel thiazole hybrid compounds as promising selective EGFR TKIs.

## Graphical abstract

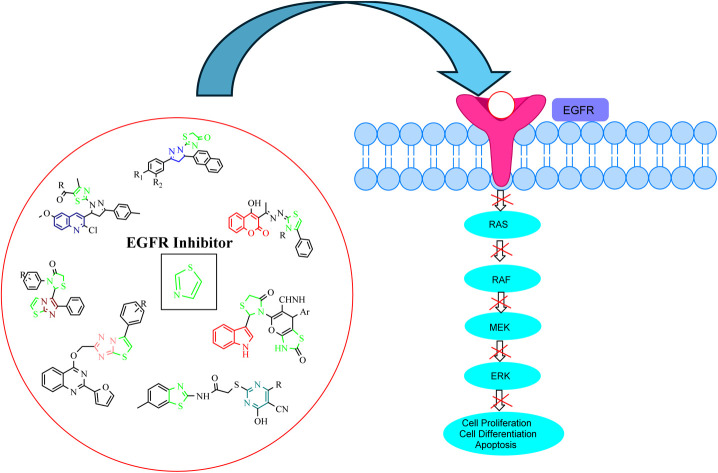

## Introduction

Cancer stands as the second leading cause of death across the globe, representing one of the most critical challenges to human health.^1^ As per the World Health Organisation nearly 10 million lives were lost to cancer in 2020, with the highest prevalence seen in breast, prostate, skin, and gastric cancers. Cancer cases are increasing at an alarming rate, largely driven by significant lifestyle shifts in recent years. Presently, cancer accounts for roughly 21% of global deaths annually, causing around 7.6 million fatalities, with projections indicating a rise to 13 million by 2030. Despite advancements in the development of sophisticated cancer chemotherapy strategies, a fully effective and curative treatment remains elusive.^2^ At present, cancer chemotherapeutic agents often lack adequate selectivity and specificity for cancerous cells or tissues, poising a major limitation. As a result, developing chemotherapy that precisely targets tumours remains one of the greatest challenges faced by chemists and oncologists.^3^ Over the past decade, significant progress has been made in cancer therapy, including the development of numerous new chemotherapeutic agents. However, these drugs often exhibit high cytotoxicity and can trigger resistance, ultimately diminishing their therapeutic effectiveness. As a result, the quest to develop safer and more effective anticancer drugs remains a complex and pressing challenge for medicinal chemists around the world,^4,5^ The adverse effects associated with conventional non-selective chemotherapy, along with the development of resistance to existing treatments, have intensified the pursuit of novel and more effective chemotherapeutic agents that minimize the harmful effects to healthy cells.^6^ Consequently, targeted chemotherapy has become a highly viable path for cancer treatment. Among the most effective and widely used approaches in cancer treatment is the inhibition of protein kinases, owing to their vital function in controlling essential cellular activities, including signal transduction, cell growth, differentiation, programmed cell death, survival, metabolism, and gene expression.^7^

The epidermal growth factor receptor (EGFR), a tyrosine kinase, plays a key role in regulating various biological functions, including cell movement, cell cycle progression, and cell adhesion.^8^ It was also the first discovered receptor tyrosine kinase associated with cancer.^9^ It is a transmembrane glycoprotein with a molecular weight of approximately 170 kDa and features a potential glycosylation at its *N*-terminal end.^10,11^ EGFR is a catalytically active receptor tyrosine kinase (RTK) that operates under strict regulatory mechanisms. It is a member of the ErbB/HER family of ligand-activated RTKs, which also includes ErbB2/Neu/HER2, ErbB3/HER3, and ErbB4/HER4.^12^ Like other receptor tyrosine kinases (RTKs), EGFR is composed of three key structural regions: an extracellular domain responsible for ligand binding, a transmembrane segment that anchors the receptor in the cell membrane, and a cytoplasmic domain that contains the tyrosine kinase responsible for signal transduction.^11^ EGFR is capable of binding to a variety of ligands, such as epidermal growth factor (EGF), transforming growth factor-α (TGF-α), betacellulin (BTC), epiregulin (EPR), heparin-binding EGF-like growth factor (HB-EGF), and amphiregulin (AR).^13^ In the absence of a ligand, EGFR remains as a monomer on the cell surface. Upon ligand binding, it undergoes dimerization, forming either homodimers with another EGFR molecule or heterodimers with other members of the ErbB family.^13^ Ligand-induced dimerization of EGFR activates the receptor's intrinsic kinase activity, resulting in the autophosphorylation of distinct tyrosine residues located in the cytoplasmic region of each monomer.^14,15^ The phosphorylated tyrosine residues act as docking sites for various adapter and signaling proteins, triggering multiple intracellular signaling cascades downstream of the receptor. Among the most well-known EGFR mediated pathways are the RAS-RAF-MEK-ERK pathway, the PI3K-AKT pathway, and the PLCᵧ-PKC pathway, all of which play key roles in promoting cell proliferation, movement, and survival upon activation.^16^

EGFR signaling is frequently disrupted in various human cancers, which can result from gene amplification, increased protein expression, activating mutations, or in-frame deletions within the EGFR gene.^17^ In numerous instances, genetic changes in EGFR lead to abnormal receptor trafficking, resulting in enhanced signaling activity and promoting tumor growth. For example, elevated EGFR levels at the plasma membrane caused by gene amplification or overexpression have been shown to promote receptor homo and heterodimerization, triggering kinase activation.^18^ Specifically, heterodimers formed with the ligand-independent receptor ErbB2 are constitutively active, resistant to ubiquitination and degradation, and are predominantly recycled to the plasma membrane, resulting in prolonged signaling and continuous cell proliferation.^19,20^ Oncogenic mutations and major genetic rearrangements in EGFR, frequently identified in glioblastoma and cancers of the brain, lung, breast, and ovary, often disrupt receptor endocytosis, resulting in heightened and sustained signaling activity.^21^ In certain cases, mutations such as EGFRvIV and EGFRvV directly interfere with the binding site for the E3 ligase Cbl on the receptor's intracellular domain, impairing its ubiquitination and subsequent lysosomal degradation.^22^ Certain mutations, such as EGFRvIII, occur in the extracellular domain and lead to ligand-independent activation of the receptor while simultaneously causing reduced phosphorylation of tyrosine residue 1045, the key Cbl-binding site, through an as-yet unidentified mechanism. As a result, receptor ubiquitination and degradation are disrupted, leading to prolonged signaling activity,^23^ as a result ubiquitination are disrupted, leading to prolonged signaling activity. Somatic activating mutations in EGFR have been identified in approximately 15–20% of patients with non-small cell lung cancer (NSCLC).^24^

Owing to its crucial role in cancer progression, a range of EGFR-targeted therapies have been developed, including humanized monoclonal antibodies that bind to its extracellular domain and selective small-molecule inhibitors that block its tyrosine kinase domain. Several of these small-molecule inhibitors, such as gefitinib, erlotinib, and afatinib, have been authorized as initial treatment options for lung cancer patients harbouring confirmed EGFR mutations.^25,26^ Cetuximab and panitumumab are also the most frequently used monoclonal antibodies that target and neutralize EGFR, commonly utilized in the treatment of head and neck cancers as well as metastatic colorectal cancer.^27–29^ Mechanistically, these agents function by blocking ligand binding, thereby suppressing receptor activation and subsequent downstream signaling. They also promote EGFR dimerization, leading to internalization of the antibody bound receptor dimers. Unlike EGF-bound dimers, these complexes are internalized more slowly and are more effectively recycled back to the plasma membrane.^30,31^ The simultaneous use of anti-EGFR antibodies targeting different, nonoverlapping epitopes has shown greater effectiveness than single-antibody approaches, as it promotes increased EGFR internalization and degradation, thereby offering the potential to enhance antitumor efficacy by regulating EGFR trafficking.^32,33^ Although EGFR-targeted antibodies and small-molecule inhibitors have shown clinical utility, their therapeutic effectiveness is often limited, and patients frequently develop resistance. This resistance may stem from (a) secondary mutations in the EGFR gene itself like the T790M mutation in non-small cell lung cancer or alterations in the extracellular domain associated with cetuximab resistance in colorectal cancer, (b) modifications in parallel signaling molecules including c-MET, PIK3CA, BRAF, and MAPK1, or (c) the activation of alternative signaling routes and compensatory feedback mechanisms that circumvent EGFR blockade.^34^ An emerging area of interest in cancer therapy is exploring how membrane trafficking affects the effectiveness of EGFR-targeted treatments. Given that EGFR is a well-established target for anticancer therapies, one of the most efficient approaches to exhibit its activity involves using small-molecule inhibitors to block the ATP-binding site within its cytoplasmic tyrosine domain.^12^ Advancements in this field may enhance therapeutic efficacy and help prevent or delay the development of resistance, a common challenge observed in most patients.^35^

Although drug discovery has progressed rapidly the major unresolved challenge remains the limited effectiveness, safety, and selectivity of currently available anticancer drugs. A wide range of heterocyclic compounds has been studied for their ability to inhibit receptor tyrosine kinases (RTKs) as a strategy for cancer treatment.^36^ Among the diverse range of heterocycles, the thiazole ring holds significant importance due to its presence in many biologically active molecules, making it one of the most thoroughly researched heterocyclic systems. Recent studies have highlighted the importance of the thiazole core in drug design and the development of new therapeutic agents. As a versatile five-membered heterocycle, the thiazole plays multiple roles in lead identification and optimization serving as a pharmacophoric group, a bio isosteric substitute, and a molecular spacer. Moreover, incorporating a thiazole ring into drug molecules can significantly influence their physicochemical and pharmacokinetic properties.^37,38^

Over the years, molecular hybridisation has emerged as a valuable strategy in drug design, enabling researchers to develop novel hybrid chemical entities (NHCEs) with promising therapeutic benefits. Combining two pharmacophores into a single molecular structure is a well-established method for enhancing drug potency and biological activity. Compared to traditional drug combinations, hybrid molecules targeting multiple pathways offer advantages such as improved pharmacokinetic and pharmacodynamic consistency and better patient adherence through single-drug therapy.^39–41^ This review focuses on the EGFR inhibitory activity of thiazole-based hybrid compounds, specifically those incorporating other heterocyclic moieties such as pyrazole, quinoline, imidazole, coumarin, triazole, indole, thiophene, pyrimidine, and pyridine. It summarizes their anticancer potential and their role in EGFR inhibition.

To date, no FDA-approved EGFR inhibitors feature a thiazole ring as the core structural component of their primary pharmacophore. Most approved EGFR-targeting drugs are based on quinazoline or aniline scaffolds. However, in recent years, thiazole-containing compounds have gained considerable attention in preclinical studies and drug development pipelines due to their favourable properties, including (1) high metabolic stability, (2) capability to form π–π stacking and hydrogen bonding interactions within the ATP-binding pocket, and (3) potential to overcome resistance mutations such as T790M. Despite their promise, thiazole-based EGFR inhibitors have yet to achieve FDA approval. This review outlines a novel approach to cancer-targeted therapy by focusing on the inhibition of the EGFR kinase domain. It presents a comprehensive summary of research conducted between 2015 to 2025 on the development of thiazole-based heterocyclic hybrid molecules as small molecule EGFR tyrosine kinase inhibitors. Additionally, it delves into future direction and design strategies aimed at enhancing the selectivity and efficacy of these thiazole hybrids.

### Thiazole–pyrazole hybrids

**Scheme 1:**
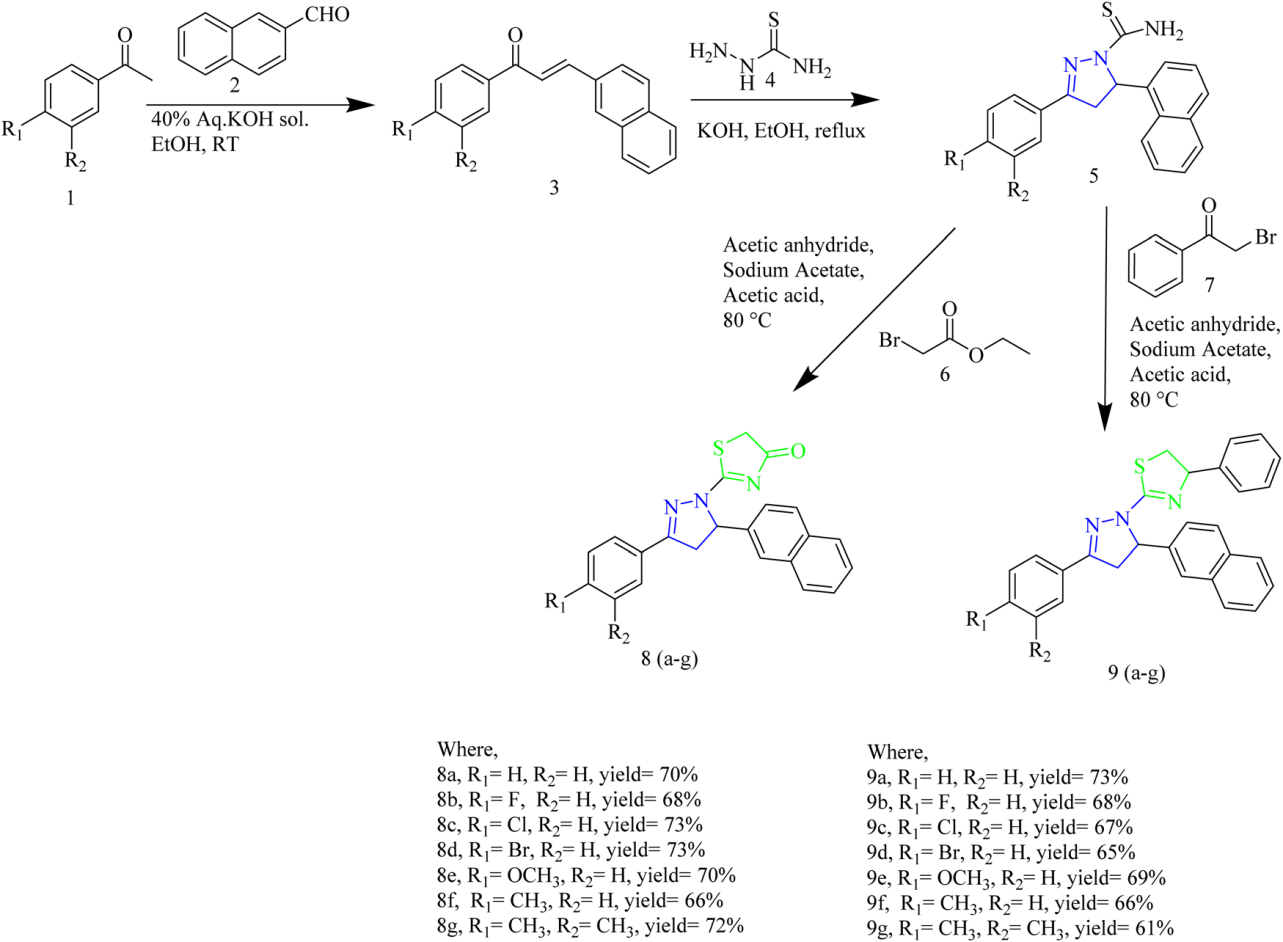
Synthesis of compound 8(a–g) and 9(a–g).

Yuan and co-workers synthesized antitumor agents containing thiazole and pyrazole rings (Table 1) as heterocyclic hybrids along with naphthalene ring. The synthesis involved condensation of naphthaldehyde 2 with aromatic ketone 1 to yield a chalcone 3. The product 3 was then treated with thiosemicarbazide 4 to yield the intermediate 5. The final products 8 and 9 were obtained by reacting the intermediate 5 with bromoacetic acid 6 or 2-bromo-1-phenylethanone 7 (Scheme 1). The compound 8g was found to be more potent compound. The IC_50_ value of 8g against EGFR was found to be 0.12 µM and it showed the IC_50_ value of 0.86 µM against HeLa cell lines. SAR analysis revealed that electron-donating groups improved antiproliferative activity. The presence of two methyl groups in compound 8g contributed to its enhanced potency. From docking studies, it was evident that two π–π bonds between naphthalene ring of 8g and the residue LYS721 in the EGFR active region enhanced the binding affinity.^42^

**Table 1:** Overview of EGFR inhibition activity of thiazole–pyrazole hybrids

Compound	Key substituent	Major interactions	EGFR IC_50_ (µM)	SAR observation
8g	2,5-Dimethyl groups on aromatic ring	π–π stacking with Lys721	0.12	Electron-donating methyl groups enhanced activity
13e	Adamantane cage	Hydrophobic interactions with Leu718 and Val726	—	Bulky hydrophobic moiety enhanced cell permeability
18k	3-Chloro phenyl ring	H-bond with Met769 and π-cation interaction with Lys721	0.07	Halogen substitution improved binding affinity
24f	4-Cyano phenyl	H–π bond with Lys721	4.34	Electron-withdrawing group improved selectivity
35a	C=NNH- linker	Six hydrogen bonds with residues Met769, Leu694, Lys721, and Gly772	—	Hydrazone linkage improved orientation and binding
50b	4-Methylthiaozle ring	H-bond with Thr790, Met793, Leu844, and Asp855	0.06	Extended conjugation enhanced dual inhibition
62e	4-Methoxy group	H-bond with Met793, Pro749, Arg841, Cys797, Gly796, and Leu792	0.009	Pyrazoline moiety stabilized hinge region binding
75d	Ester (C-5) and amide-phenyl substitutions on thiazole	H-bonds with Met769, Gln767, Cys751, Thr766	32.5	Ester and amide groups improve H-bonding and binding orientation also aromatic substitution boosts π interactions and dual EGFR/VEGFR activity
86b	p-hydroxy phenyl group	H-bond with Met769, water hydrogen bond bridge with Thr766 and cation-π bond with Lys721	83 nM	Binding mode similar to erlotinib's quinazoline ring due to its orientation towards the hydrophobic residues
95g	Fluoro-phenyl	H-bond with Met769 and Leu768	267 nM	Presence of the small fluorine substituent enhanced the compound's fit and interaction efficiency within the EGFR binding pocket
100g	4-Cl-phenyl on thiazole	H-bond with Leu694, Asp831, and Lys721	0.18	Electron withdrawing groups improve EGFR affinity
106b	Electron-rich rings	H-bond with Met793, Lys745, Val726, Cys797, and Arg841	0.024	Electron rich ring increased EGFR/HER2 activity
127c	Bis-thiazole–pyrazolyl hybrids with naphthalene moiety	H-bond with hinge residues	4.98 nM	Doubling pharmacophores (bis-thiazole units) significantly increases potency through multivalent interactions
136e	Naphthyl moiety	π cation interaction with Lys721	—	Extended aromaticity improved affinity
139	Benzothiazole-pyrazolidine-dione	H-bonds with Arg836, Lys860, Gly874 residues		Benzothiazole core and pyrazolidine-dione enhance hydrogen-bond formation and π–π stacking
143	Acetamidobenzothiazole–pyrazole hybrid	Fits well in EGFR pocket with favorable geometry	0.239	Acetamide linker and planar benzothiazole-pyrazole system favor selective EGFR binding due to optimal pocket fitting

**Scheme 2:**
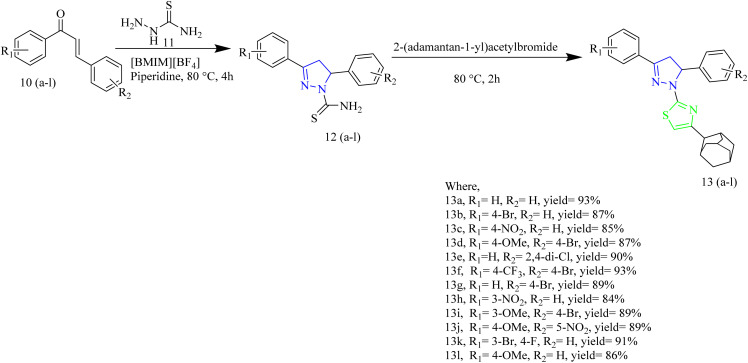
Synthesis of compound 13(a–l).

A series of novel adamantanyl-based thiazole pyrazole hybrids designed to target EGFR protein present in triple-negative breast cancer was synthesized by Sebastian and co-workers. The synthetic route involved the reaction of chalcones 10(a–l) with thiosemicarbazide 11 at 80 °C using an ionic liquid [BMIM][BF_4_] in presence of catalytic amount of piperidine. The intermediate thus obtained 12(a–l) was treated with 2-(adamantan-1-yl)acetyl bromide at 80 °C for 2 h to yield the final product 13(a–l) (Scheme 2). Based on the reduction of cell vitality in triple negative bacteria cells the compound 13e was found to be the potent compound among the series with the IC_50_ value of 4.9 µM in BT549 cell lines. From SAR analysis it was found that incorporation of a dichlorophenyl group on the pyrazole ring, as seen in compound 13e, markedly boosted both antiproliferatve effects and EGFR inhibitory activity. The adamantane unit enhanced membrane penetration and optimized pharmacokinetic behaviour. Additionally, electron-withdrawing substituents such as halogens positioned on the aryl ring contributed to better fit and stronger interactions within the EGFR binding site. 13e efficiently reduced the cell survival by inhibiting phosphorylation at critical sites, which targets EGFR signalling.^43^

**Scheme 3:**
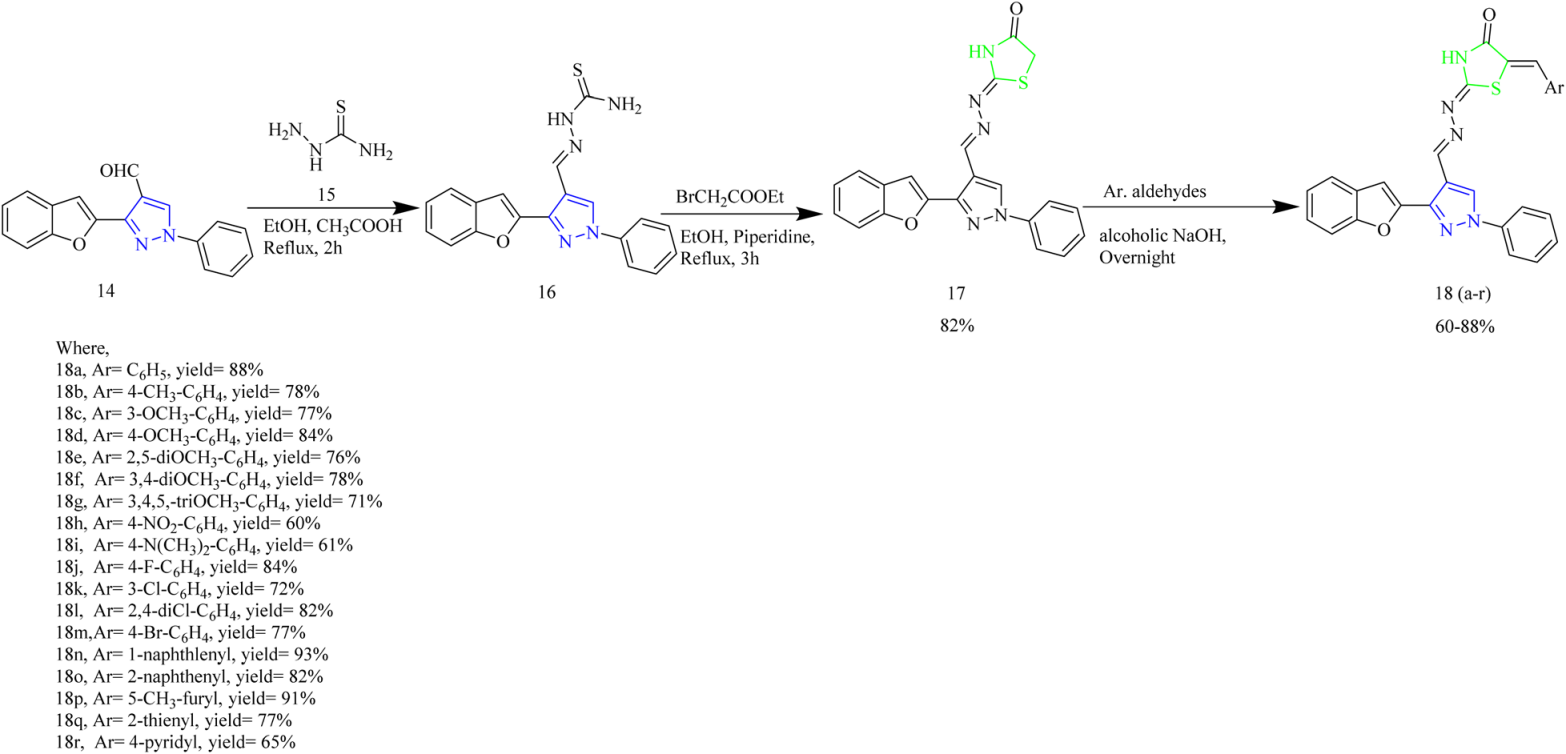
Synthesis of 18(a–r).

Abbas *et al.* synthesized benzofuran containing pyrazole–thiazole hybrids. Firstly, the intermediate 16 was synthesized by condensation of 14 with thiosemicarbazide 15. This intermediate was then treated with ethyl bromoacetate with few drops of piperidine using ethanol as a solvent to yield the compound 17. The target molecules 18(a–r) were synthesized *via* Knoevenagel condensation reaction of thiazolidine intermediate 17 with aromatic aldehydes in alcoholic NaOH condition (Scheme 3). The target compounds 18(c,d,h,j,k,m,n) showed the anticancer activity more than or equal to that of standard drug doxorubicin. And the compound 18k had higher EGFR PK inhibitory action than the reference drug erlotinib (where the IC_50_ values are 0.07, 0.08 µM respectively). SAR analysis revealed that introducing a meta-chlorine (3-Cl) group on the phenyl ring as seen in compound 18k, improved both binding affinity and electron-withdrawing nature of the molecule, thereby enhancing its interaction with the target. Additionally, the halogen atom was found to increase the lipophilicity of the compound, which likely facilitated better cell membrane penetration and elevated intracellular accumulation. From docking studies, it was found that the compound 18k interacts with key amino acid residue Met769 *via* hydrogen bonding, Thr766 *via* water-mediated H-bonding, and Lys721 *via* cation-π interaction through its *m*-chlorophenyl moiety.^44^

**Scheme 4:**
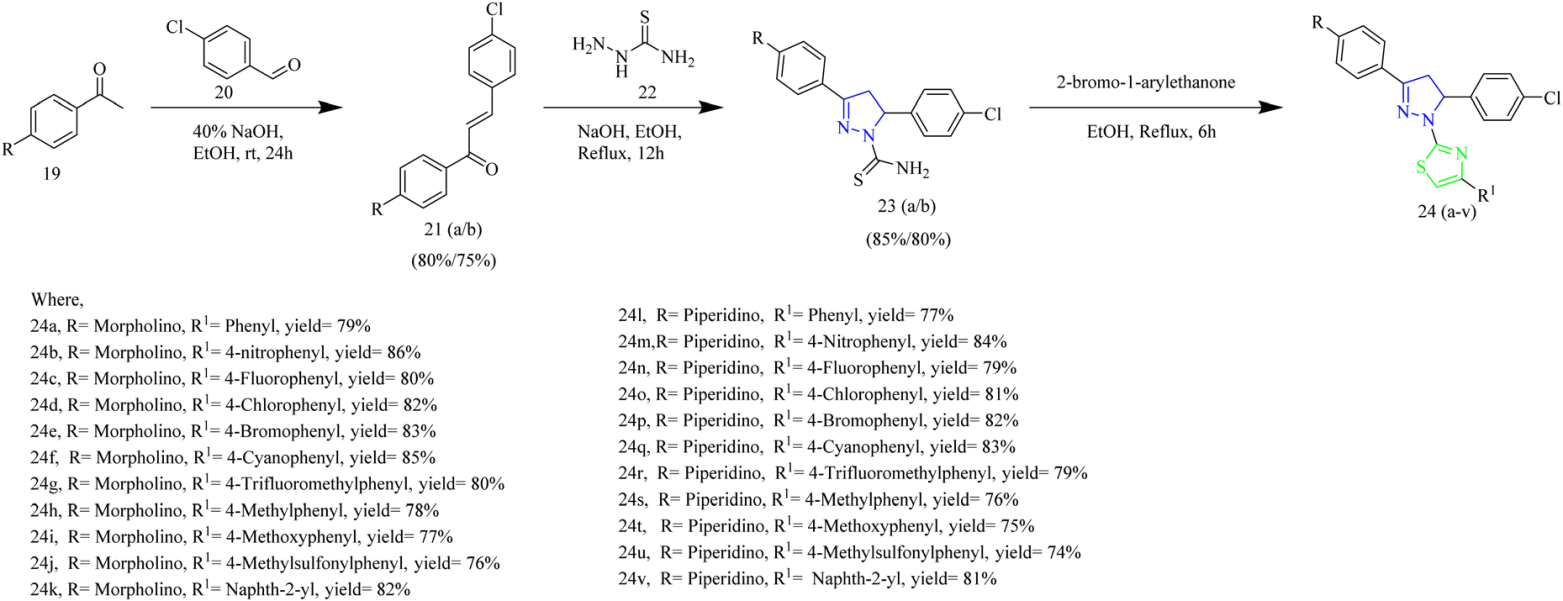
: Synthesis of 24(a–v).

Sever *et al.* developed a novel series of thiazolyl-pyrazolines that functions as dual HER2 and EGFR inhibitors. The target compounds 23(a–v) were synthesized using a multistep process that involved condensation of 4-chlorobenzaldehyde 20 with aromatic ketone 19 which resulted in chalcone formation of chalcone intermediate 21 which was then cyclised with thiosemicarbazide 22 to afford an intermediates 23(a/b) which then underwent ring closure to yield target molecules 24(a–v) when treated with 2-bromo-1-arylethanone (Scheme 4). When compared to erlotinib, the compounds 24(c, f, q) were determined to be the most effective anticancer agents against A549 and breast cancer cell lines (MCF-7). In addition to this, the compound 24f with a cyanophenyl substitution showed dual inhibition of EGFR with IC_50_ = 4.34 µM and HER2 with IC_50_ = 2.28 µM, which are the key targets in cancer therapy. The key SAR findings indicated that incorporating a 4-cyanophenyl group on the thiazole ring enhanced binding through π–π and H–π interactions. Its polar nature also facilitated a favourable alignment within the kinase binding site. It also contributed to the favourable orientation within the kinase pocket due to its polar nature. The cyanophenyl group was found to form a crucial H–π bond with Lys721 residue of the EGFR protein which is absent in the other molecules of the series. Although the compound 24f claimed to be potent it demonstrated IC_50_ value slightly higher than erlotinib which limits its efficiency in additional research.^45^

**Scheme 5:**
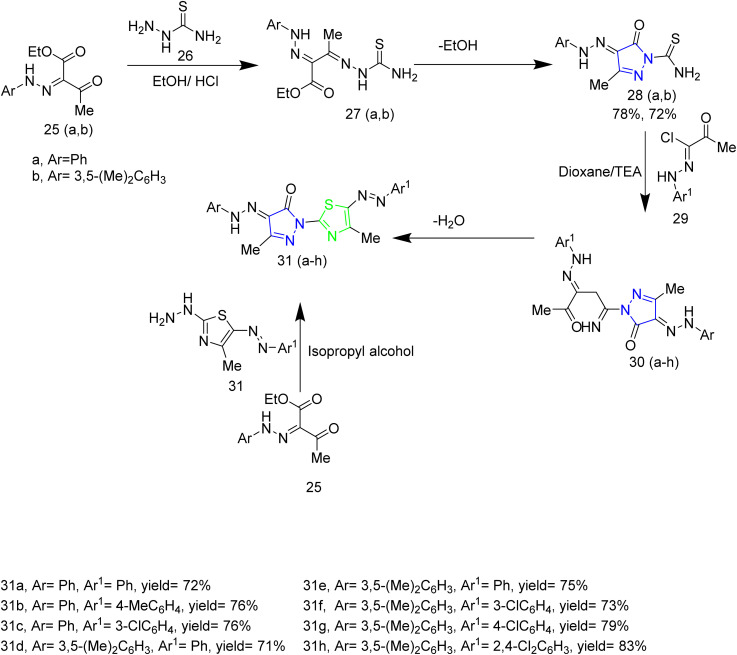
Synthesis of 31(a–h).

**Scheme 6:**
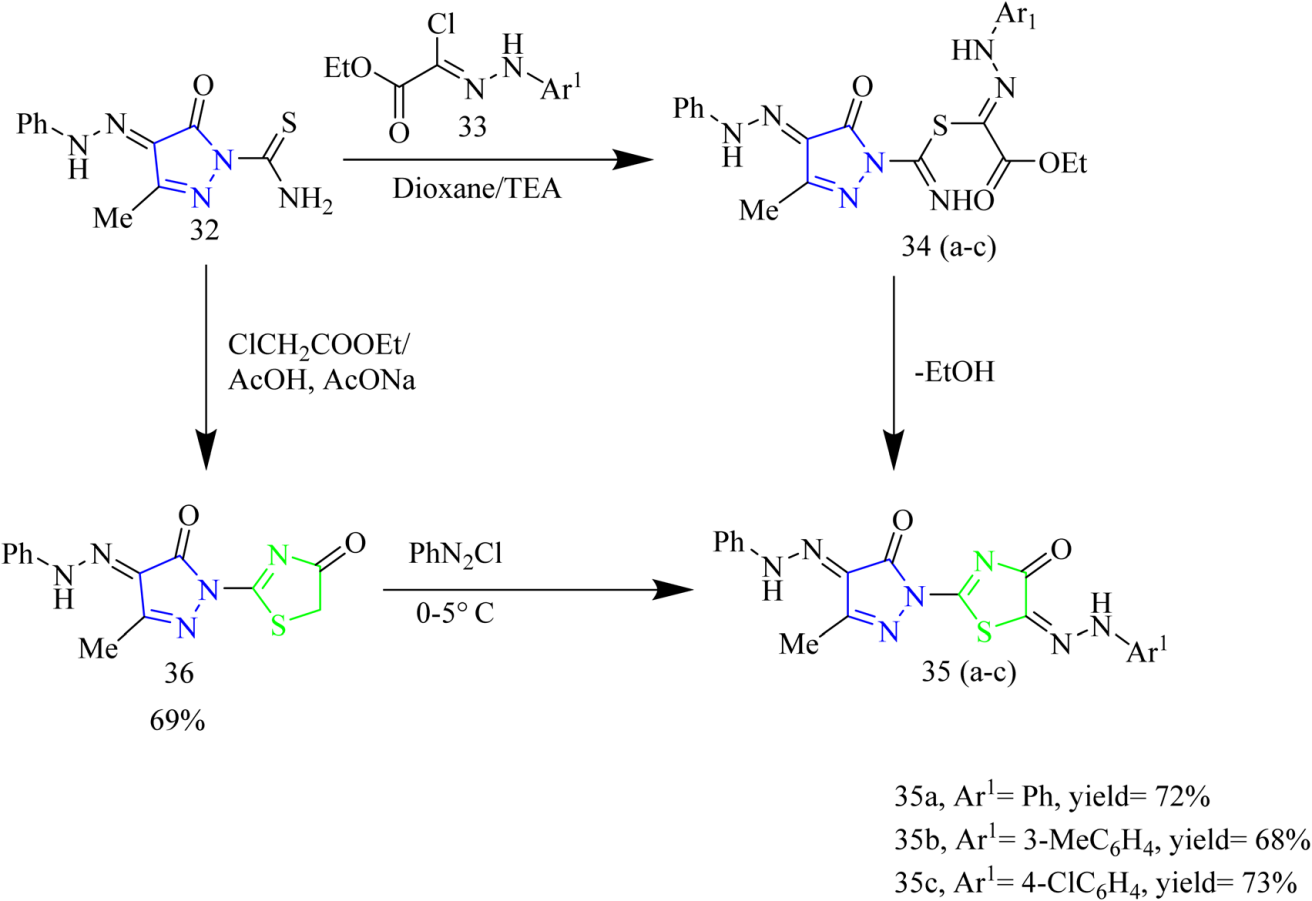
Synthesis of 36 and 35(a–c).

**Scheme 7:**
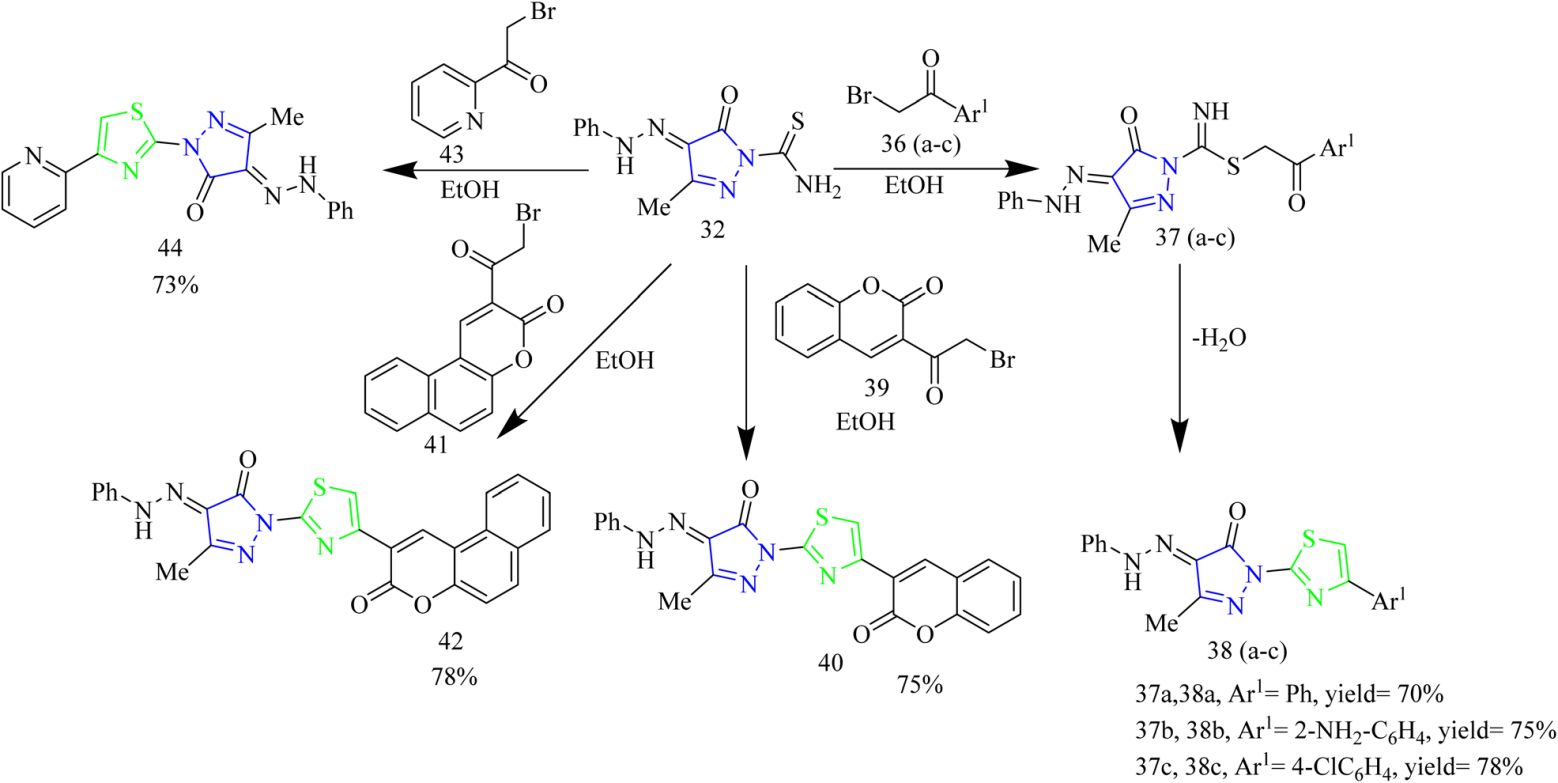
Synthesis of 38(a–c), 40, 42, and 44.

A number of thiazolyl pyrazole derivatives were designed and synthesized by Abdelwahed and co-workers. The starting material 25(a,b) was reacted with thiosemicarbazide 26 in ethanol to afford intermediates 27(a,b) which then underwent cyclisation by elimination of ethanol to yield the pyrazole intermediate 28. The pyrazole intermediate 28 was then reacted with hydrazonyl chlorides 29 to form an intermediate 30(a–h) which upon elimination of water afforded desired thiazole–pyrazole hybrids 31(a–h) (Scheme 5). The pyrazole intermediate 28 was reacted with another hydrazonyl chloride forming an intermediate 34 followed by ethanol elimination to yield compounds 35(a–c) (Scheme 6). The pyrazole intermediates were also reacted with different bromoacetyl derivatives 36(a–c), 39, 41, 43 respectively which yielded various thiazole–pyrazole hybrids 37(a–c), 40, 42, 44 (Scheme 7). The compounds 28, 35d, 35a, 38a, and 44 were docked against 1M17 crystal structure and the docking scores were −1.6, −3.0, −3.4, −2.2, −1.3 Kcal mol^−1^ respectively. Among these potent compounds the compound 35a demonstrated highest anticancer activity. The compound 35a outperformed standard doxorubicin (IC_50_ = 0.07 µM) in terms of anticancer activity, with an IC_50_ value of 2.20 µg mL^−1^ against HepG-2 cell lines. The SAR analysis indicated that the hydrazone linkage in compound 35a enhances conjugation and electron delocalization, thereby strengthening its interaction with the kinase active site. Docking analysis revealed that the compound 35a exhibited the most favourable docking score of −3.4 kcal mol^−1^ among all the tested molecules and established six hydrogen bonds with important residues, surpassing the other molecules in the series in terms of number of key interactions.^46^

**Scheme 8:**
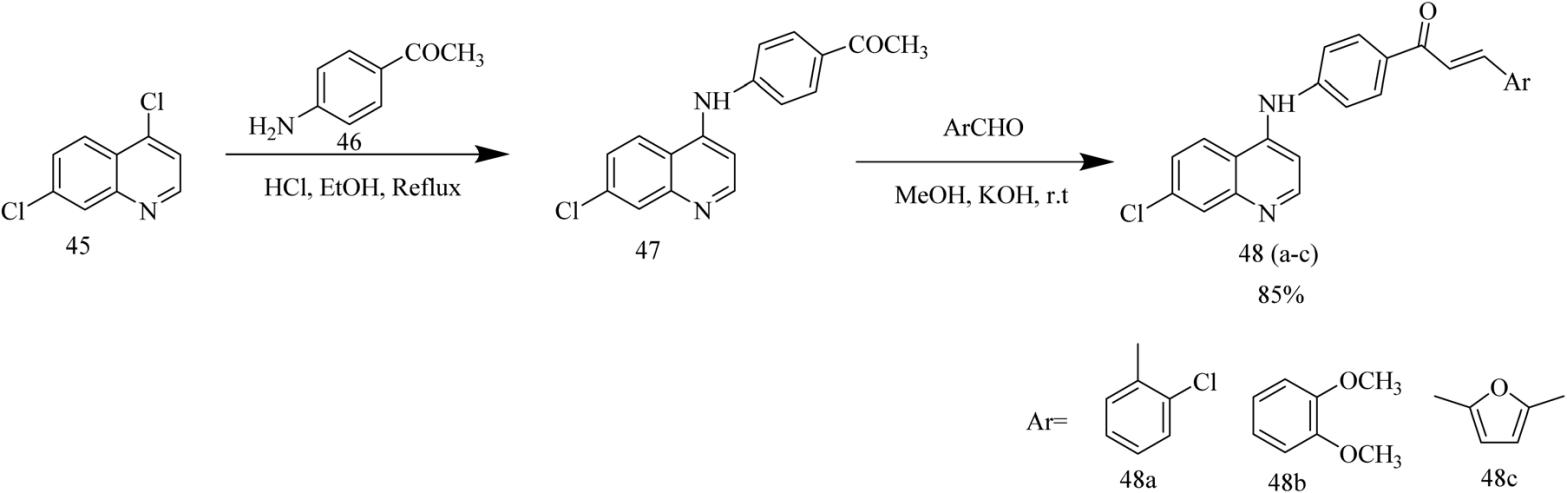
Synthesis of 48(a–c).

**Scheme 9:**
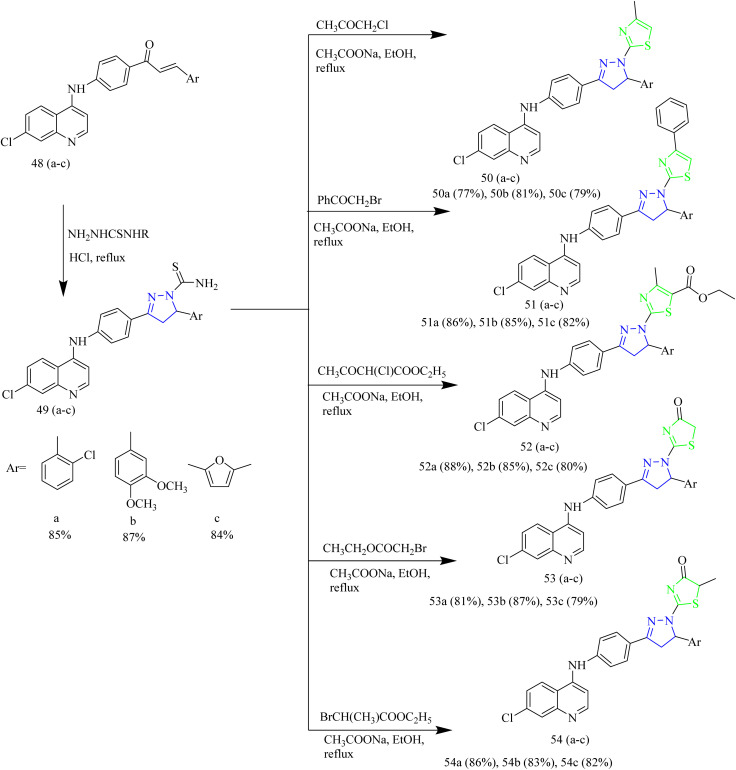
Synthesis of 50(a–c), 51(a–c), 52(a–c), 53(a–c), 54(a–c).

Thiazole pyrazoline based aminoquinoline derivatives functioning as dual inhibitor of EGFR and HER2 was reported by Batran *et al.* The chalcone intermediates 48(a–c) were synthesized *via* Claisen Schmidt condensation between acetyl compound 47 with different aromatic aldehydes. Thiocarbamoyl pyrazoline 49(a–c) intermediates were synthesized by the cyclocondensation reaction between chalcone intermediates 48(a–c) with thiosemicarbazide (Scheme 8). Thiocarbamoyl pyrazoline 49(a–c) intermediates were then reacted with various α-haloketones to yield compounds 50(a–c) and 51(a–c). Similarly, compounds 52(a–c), 53(a–c), and 54(a–c) were obtained by refluxing 49(a–c) intermediates with ethyl-2 chloroacetoacetate, ethylbromoacetate, and ethyl 2-bromopropionate (Scheme 9). The compound 50b demonstrated significant anticancer activity against HCT116, with an IC_50_ value of 3.07 µM and was then evaluated for its activity against EGFR and HER2 kinase. The EGFR inhibition assay results showed that 50b had a strong inhibitory effect with IC_50_ value of 0.06 µM in contrast to standard erlotinib which had the IC_50_ value of 0.04 µM. Likewise, the compound showed HER2 inhibition activity with an IC_50_ value of 0.08 µM in comparison to lapatinib which had an IC_50_ value of 0.05 µM. Docking studies revealed that the compound 50b demonstrated strong binding interactions by fitting inside the EGFR pocket effectively, which was illustrated by its binding energy (BE) score of −15.0774 kcal mol^−1^.^47^

**Scheme 10:**
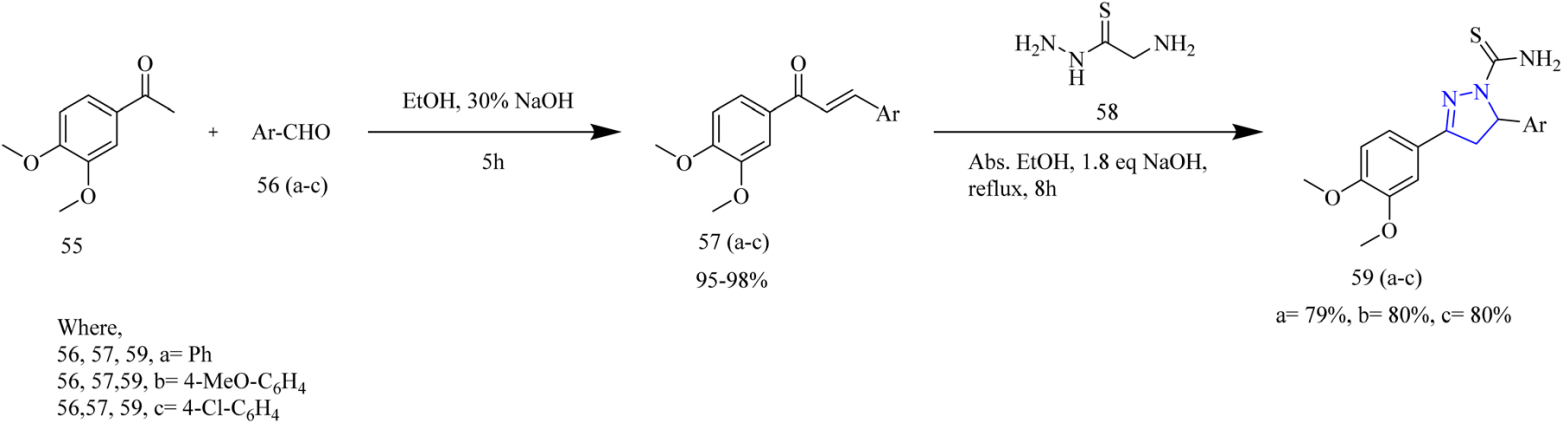
Synthesis of 59(a–c).

**Scheme 11:**
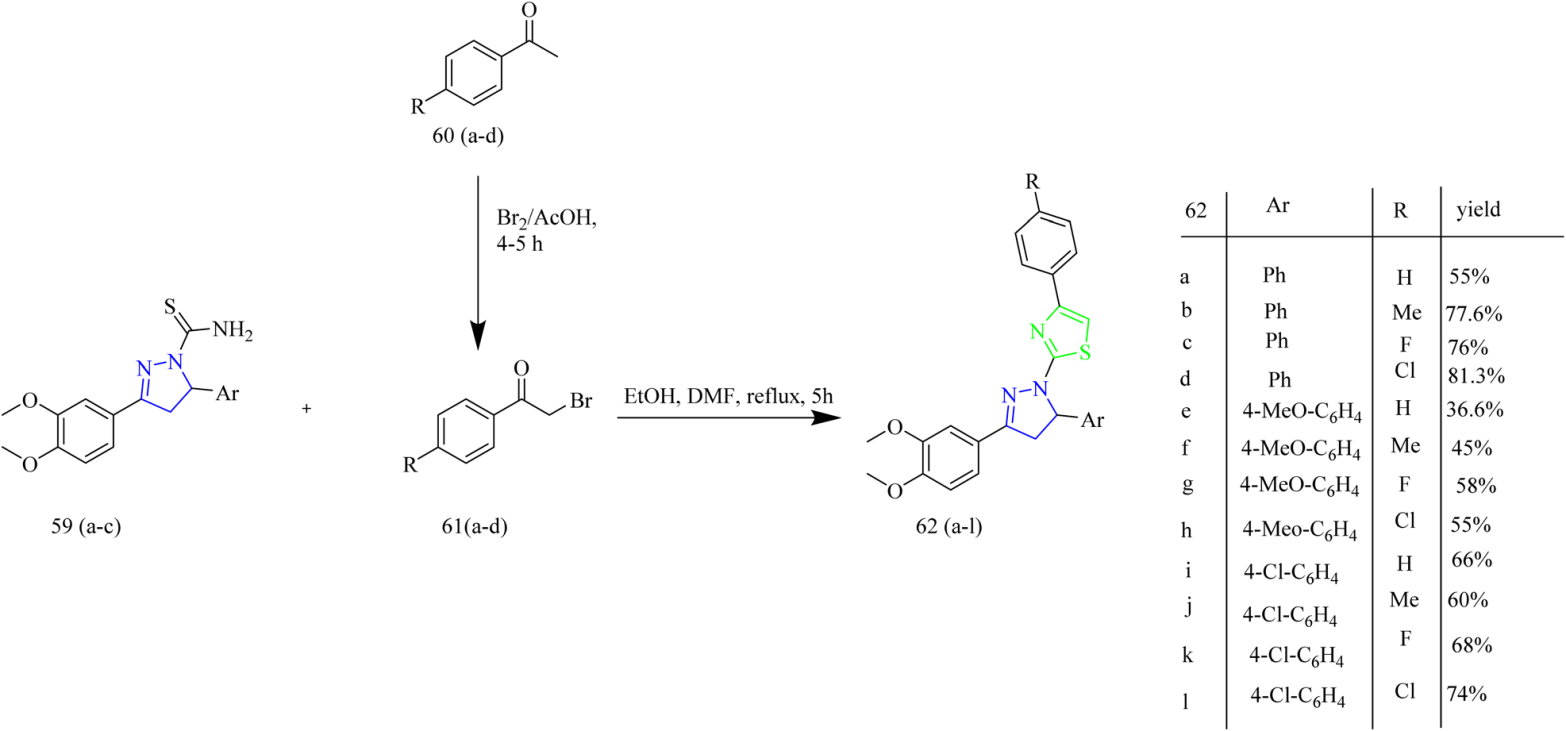
Synthesis of 59(a–c), 61(a–d), and 62(a–i).

**Scheme 12:**
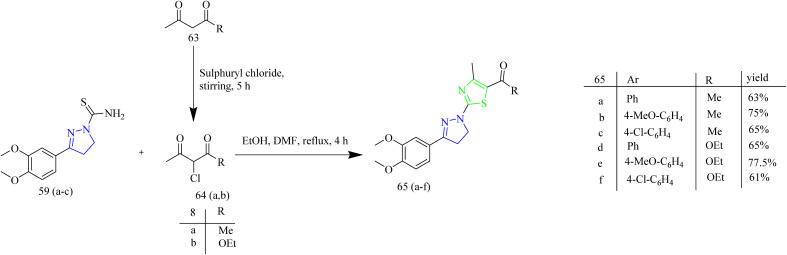
Synthesis of 65(a–f).

Fakhry *et al.* synthesized series of pyrazoline based antiproliferative agents. The synthetic route involved Claisen–Schmidt condensation between dimethoxy acetophenone 55 with aromatic aldehydes 56(a–c) to yield a chalcone intermediate 57(a–c). The chalcone intermediate 56(a–c) was reacted with thiosemicarbazide to yield pyrazole intermediates 59(a–c) (Scheme 10). Next, the pyrazole intermediates were reacted with phenacyl bromide 61(a–d) in ethanol which resulted in formation of thiazolyl-pyrazoline derivatives 62(a–l) (Scheme 11). Furthermore, compounds 65(a–f) were synthesized by the reaction of pyrazole intermediates 59(a–c) with 3-chloropentane-2,4-dione 64a or ethyl 2-chloro-3-oxobutanoate 64b in ethanol refluxing condition (Scheme 12). Compounds 62e and 62k exhibited effective antiproliferative effects on MCF-7 with corresponding IC_50_ values of 7.21 and 8.02 µM. Also, the compound 62e was found to inhibit the activity of EGFR and HER2 enzymes with IC_50_ values of 0.009 and 0.051 µM for EGFR and 0.013 and 0.027 µM for HER2 which could be due to the incorporation of thiazole and pyrazoline rings. SAR studies revealed that the presence of the pyrazoline ring in the synthesized compounds played a crucial role in enabling key interactions within the hinge region of the kinase domain. The methoxy phenyl group was found to boost the activity of the target compound by forming an interaction in the hydrophobic II pocket.^48^

**Scheme 13:**
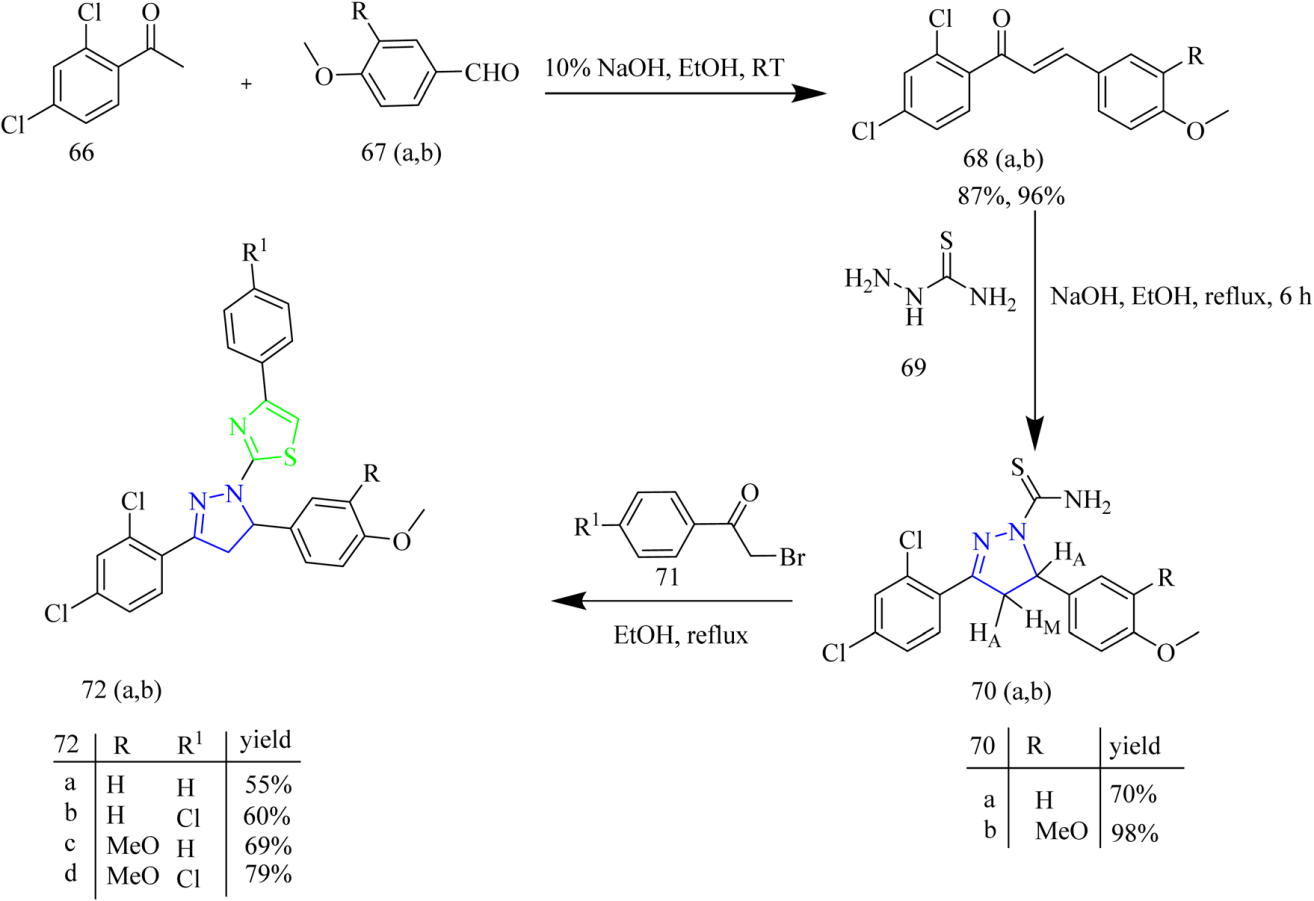
Synthesis of 72(a,b).

**Scheme 14:**
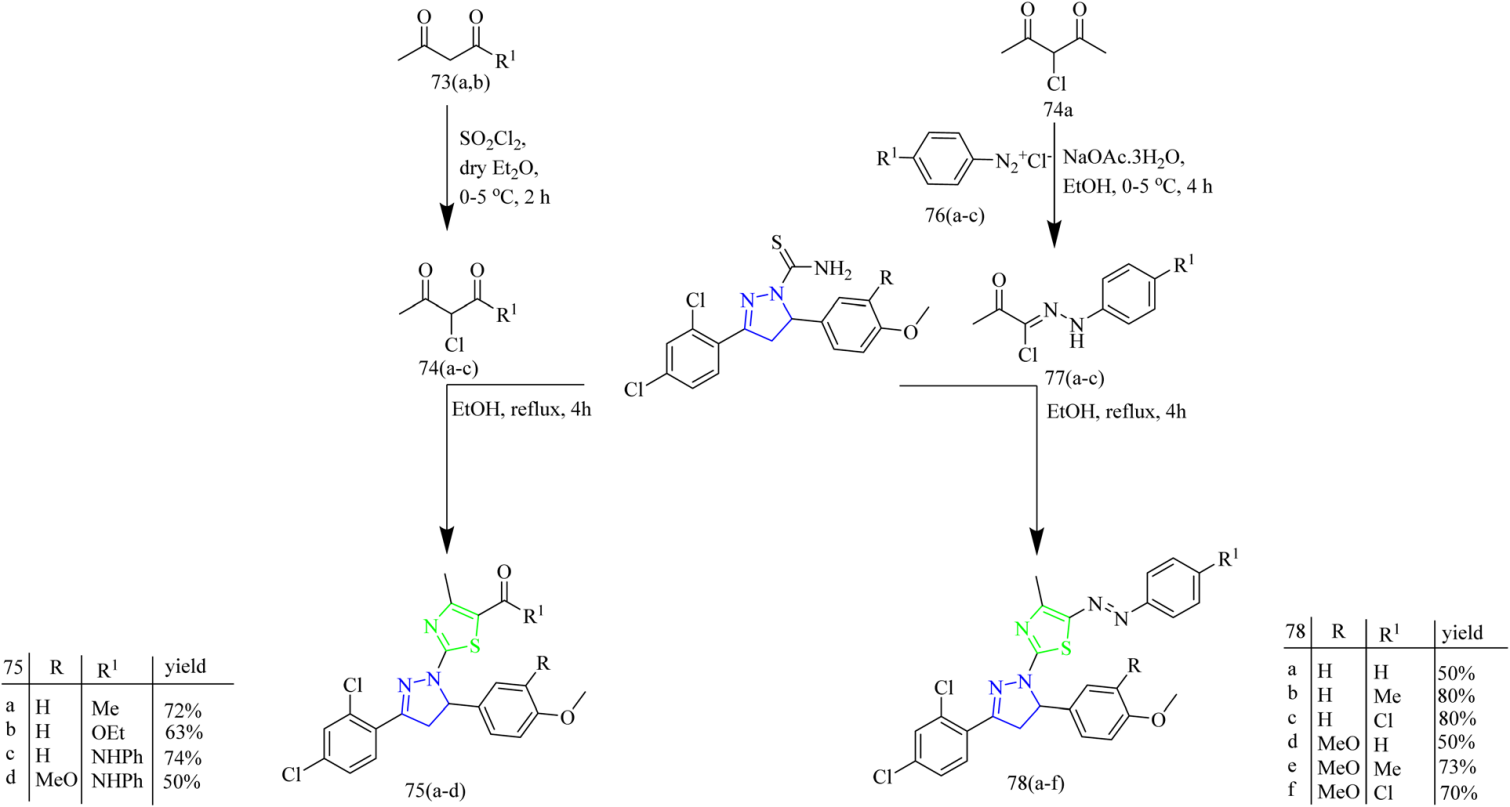
Synthesis of 75(a–d) and 78(a–f).

Thiazolyl-pyrazoline compounds for inhibition of non-small lung cancer was reported by Abdelsalam and co-workers. First, the direct condensation of dichloroacetophenone 66 with aromatic aldehydes 67(a,b) led to the formation of chalcone intermediate 68(a–b). The obtained chalcone intermediates were treated with thiosemicarbazide 69 in presence of base to yield corresponding carbothioamide intermediates 70(a,b). The obtained intermediates then reacted with phenacyl bromide 71 leading to the formation of a thiazole ring 72(a,b)*via* Hantzsch thiazole synthesis (Scheme 13). Similarly, compounds 75(a–d) were synthesized using appropriate α-chloro-1,3-dicarbonyl compounds 74(a–c) Finally, compounds 78(a–f) were obtained by heating the intermediates 70(a,b) with oxo-*N*-arylpropanehydrazonyl chlorides 77(a–c) (Scheme 14). 77(a–c) intermediates were obtained as a result of coupling reaction between aryl diazonium salts 76(a–c) and α-chloroacetylacetone 74a*via* Japp-Klingeman rearrangement. The compounds 75b and 75d were identified to have strong and specific inhibitory activity against the two receptor tyrosine kinases *i.e.*, EGFR (IC_50_ = 40.7 ± 1.0 and 32.5 ± 2.2 nM) and VEGFR-2 (IC_50_ = 78.4 ± 1.5 and 43.0 ± 2.4 nM) with the synthetic yield of 50–72%. Furthermore, compounds 75b and 75d showed significant activity against A549 and H441 cell lines with IC_50_ values of (4.2, 2.9 and 4.8 3.8 µM, respectively). From SAR analysis it was observed that the compound 75b exhibited the optimal activity due to the presence of ester group at C-5 position of thiazole ring and in case of 75d the amide group in addition to phenyl substitution contributed to its superior activity. Both the compounds demonstrated significant hydrogen-bond interactions with critical residues in EGFR and VEGFR-2, as confirmed by molecular docking experiments. The thiazolyl-pyrazoline derivatives 75b and 75d fit well into the EGFR active site, with docking scores of 12.9 and 14.1 kcal mol^−1^, respectively equal to that of erlotinib (11.7 kcal mol^−1^). The carbonyl group of ester in 75b formed a hydrogen link with Met769, as well as hydrogen bonds with Gln767, Cys751, and Thr766 *via* a water molecule.^49^

**Scheme 15:**
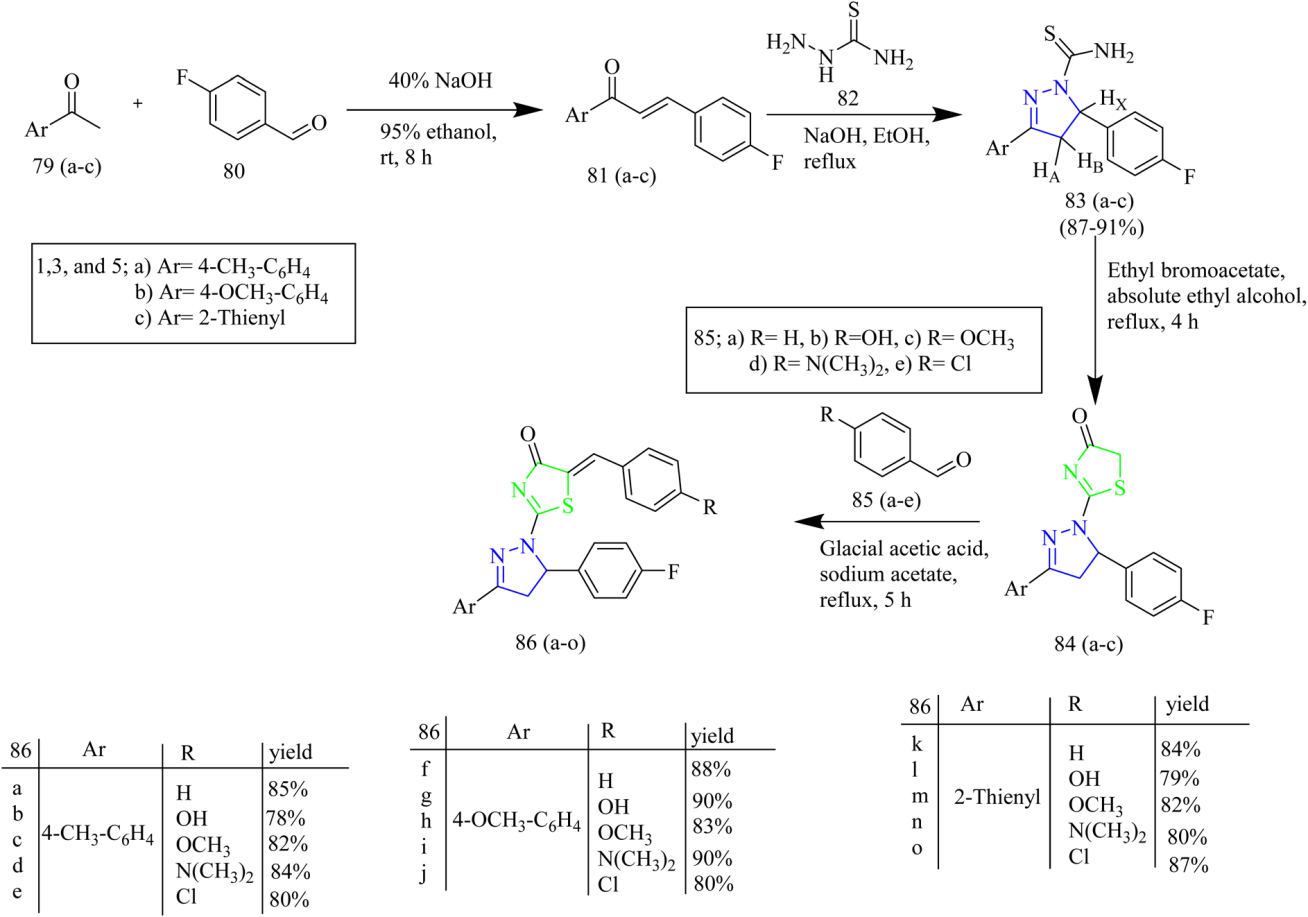
Synthesis of 86(a–o).

Al-Warhi *et al.* reported the synthesis of dihydropyrazol-1-yl thizol-4-one hybrids exhibiting EGFR inhibition. The thiazole–pyrazoline derivatives 86(a–o) were synthesized as follows (Scheme 15). Intermediates 81(a–c) were prepared by condensation of acetophenones 79(a–c) with 4-fluorobenzaldehyde 80. The prepared chalcones 81(a–c) were reacted with thiosemicarbazide in presence of base to yield pyrazoline derivatives 83(a–c). The previously prepared pyrazoline derivatives were cyclised using ethyl bromoacetate in ethanol solvent yielded cyclised products 84(a–c). Then Knoevenagel condensation reaction between cyclised products 84(a–c) with various aldehydes 85(a–e) resulted in the formation of thiazolyl-pyrazoline derivatives 86(a–o). The potential anticancer effect of newly synthesized thiazolyl-pyrazoline derivatives 86(a–o) were examined *in vitro* using the MTT test on A549 cell lines (lung cancer) and T-47D cell lines (breast cancer). It was observed that the lead compounds 86(a–o) were found to have stronger antiproliferative activity against T-47D than A549 lung cancer cell lines. Out of fifteen thiazolyl pyrazoline derivatives compounds 86b, 86g, 86l, and 86m seemed to be strong inhibitors of cell proliferation for both considered cell lines. Thiazolyl pyrazolines 86g and 86m exhibited significant activity against T-47D cells (IC_50_ = 0.88 and 0.75 µM, respectively) and A549 cells (IC_50_ = 3.92 and 6.53 µM, respectively). The most potent hits 86b, 86g, 86l, and 86m underwent additional *in vitro* biochemical analysis to test their EGFR kinase inhibitory action in relation to the authorised EGFR inhibitor erlotinib. Target compounds showed strong nanomolar inhibitory activity with IC_50_ value of 83, 262, 171, and 305 nM, compared to erlotinib (IC_50_ = 57 nM). Docking studies illustrated the ability of the target compound to interact with important EGFR residues by exhibiting a binding pattern resembling that of erlotinib.^50^

**Scheme 16:**
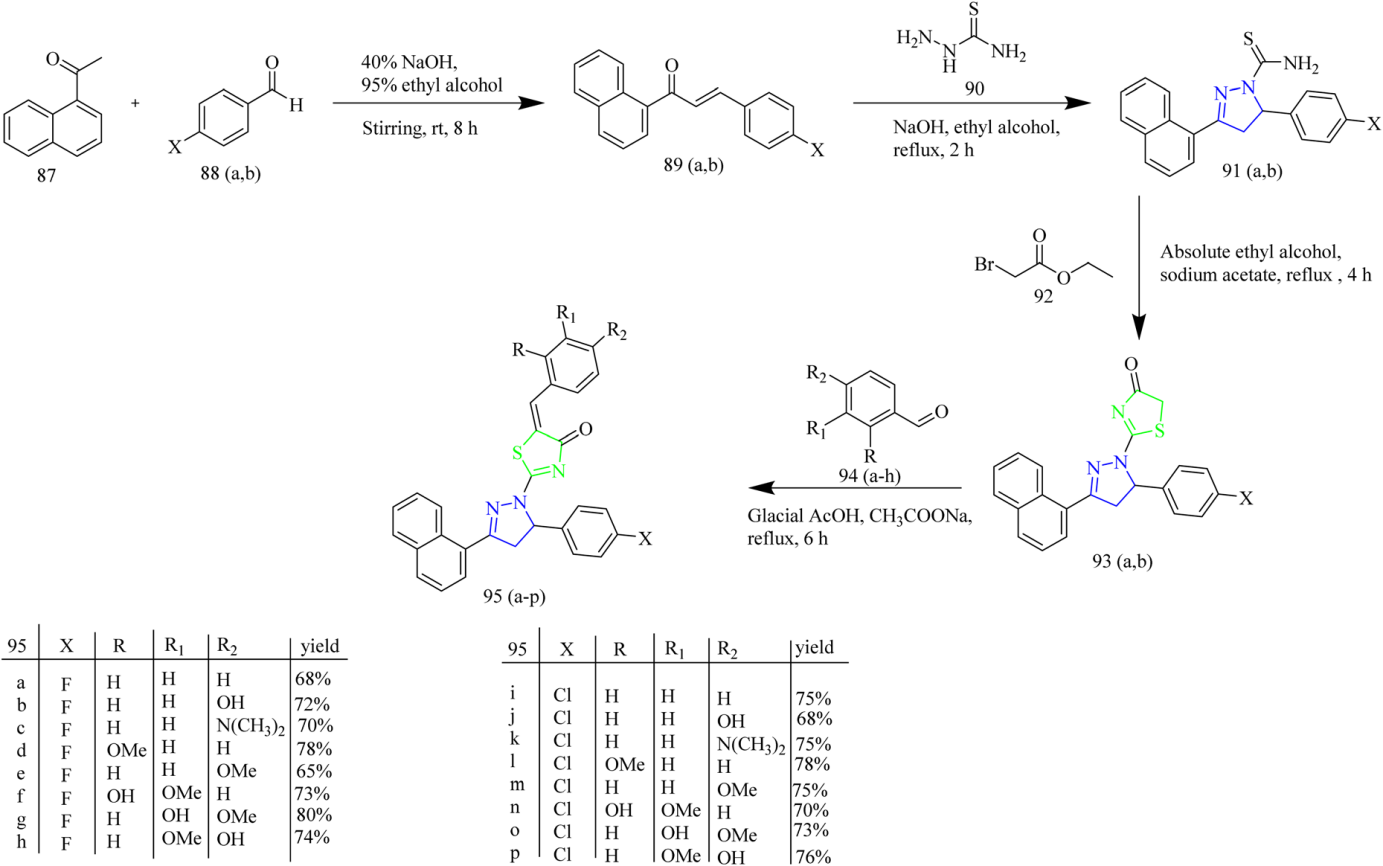
Synthesis of 95(a–p).

Naphthalene based thiazole–pyrazole hybrids were designed and synthesized by Eldehna and co-workers. The target molecules 95(a–p) were synthesized *via* multistep process involving synthesis of chalcone intermediate 89(a,b) formed by the reaction between ketone 87 with aromatic aldehydes 88(a,b) in basic medium which then underwent heterocyclization by reacting with thiosemicarbazide 90 yielded carbothioamide intermediates 91(a,b). The obtained intermediates were refluxed in ethanol with ethyl bromoacetate reagent to get intermediates 93(a,b) which upon undergoing Knoevenagel condensation with different aldehydes yielded desired thiazole–pyrazole hybrids 95(a–p) (Scheme 16). It was found that the 4-fluoro 95g and 4-chloro derivatives 95k were the most cytotoxic agents against two BC (breast cancer) cell lines with IC_50_ values of 0.62 ± 0.03 against MDA-MB-231 and 3.14 ± 0.11 µM against T-47D and 1.14 ± 0.06 against MDA-MB-231 and 4.92 ± 0.28 µM against T-47D cell lines. Consequently, the anti-EGFR activity of both compounds 95g and 95k were evaluated and they exhibited nanomolar inhibitory activity, with IC_50_ values of 267 ± 12 and 395 ± 17 nM for 95g and 95k, respectively. Remarkably, both compounds 95g and 95k docked to EGFR generated a favourable binding through a binding pattern that was exactly correlated with the reference erlotinib. Compound 95g had a docking score of −11.8 kcal mol^−1^, which was slightly better than that of compound 95k with a docking score of −11.4 kcal mol^−1^. Both the scores were in line with erlotinib's docking score of −12.0 kcal mol^−1^. The carbonyl moiety of the triazolone core in compound 95g functioned as a hydrogen bond acceptor for two H-bonds with crucial residues Met769 and Leu768. Additionally, the para methoxy group of the compound 95g interacted with Glu738 through a carbon hydrogen linkage. Similarly, compound 95k formed two hydrogen bonds with the crucial residues Leu 768 and Met 769 through its carbonyl group. The compound 95g outperformed other compounds in molecular modelling owing to the small size of the fluorine group, it fits well within the EGFR binding pocket.^51^

**Scheme 17:**
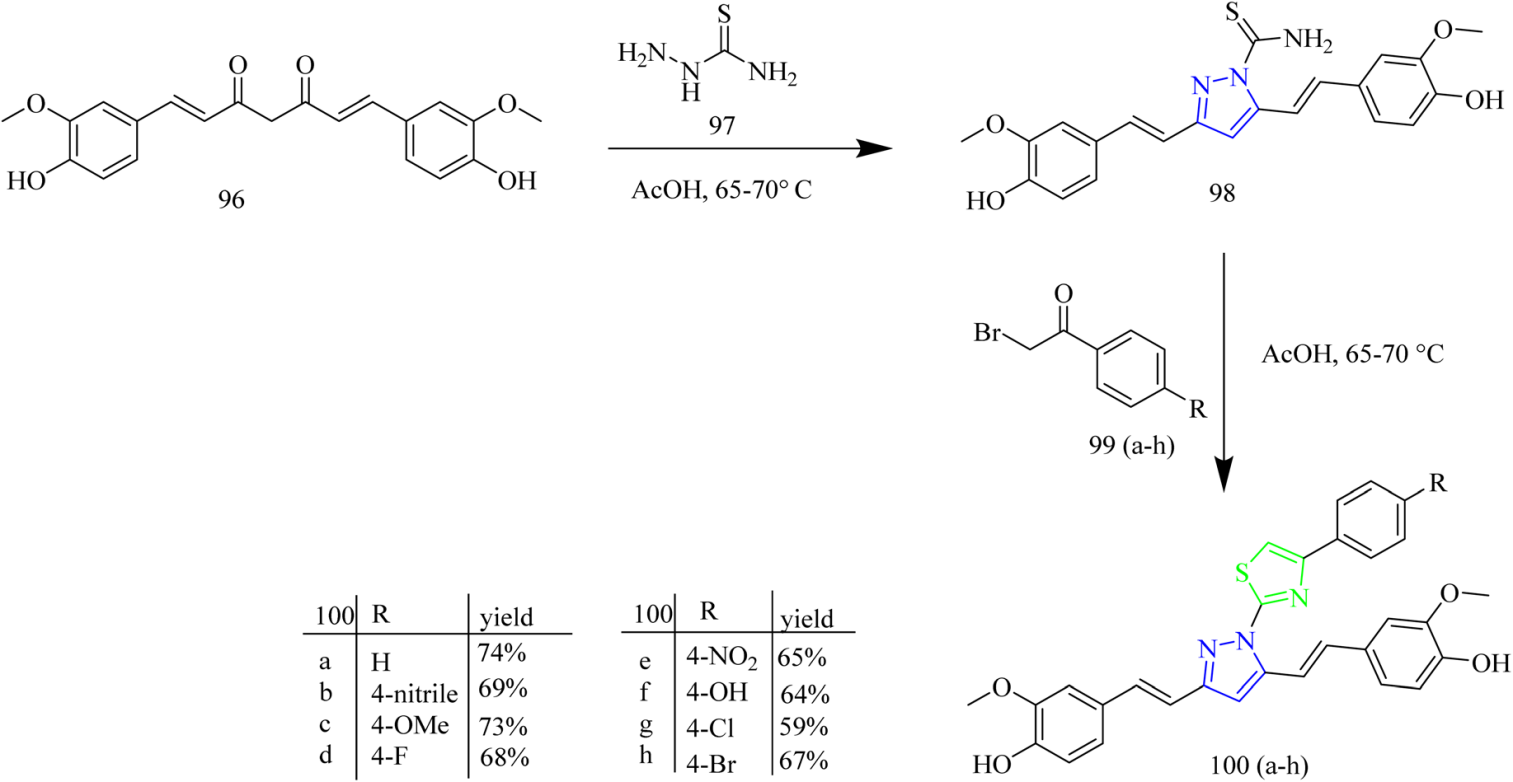
Synthesis of 100(a–h).

Palabindela *et al.* synthesized new class of thiazole–pyrazole hybrids and assessed their activity as anticancer agents targeting EGFR enzyme. The curcumin-based pyrazole containing carbothiamide 98 was prepared *via* reaction of curcumin 96 with thiosemicarbazide 97 in presence of glacial acetic acid under reflux condition. Finally, the carbothiamide intermediate 96 was reacted with different phenacyl bromides 99(a–h) to yield a thiazole pyrazole hybrid 100(a–h) (Scheme 17). The compounds 100b, 100e, and 100g were found to have highest antiproliferative effect among all examined cell lines. The compound 100g with a 4-chloro-phenyl ring on the thiazole pyrazole hybrid molecule showed excellent activity with IC_50_ values of 8.57 µM for COLO-205, 9.22 µM for MCF-7, 16.75 µM for HepG-2, 8.48 µM for A549, and 7.22 µM for HeLa cell lines. The three potent compounds 100b, 100e, and 100g were further tested for the inhibition of the EGFR tyrosine kinase. In comparison to the reference erlotinib (IC_50_ = 0.42 µM), the compound 100g has demonstrated stronger (almost twice) inhibitory activity (IC_50_ = 0.18 µM). Likewise, compound 98b was found to have better inhibitory activity than erlotinib (IC_50_ = 0.39 µM). However, compound 100e showed the inhibition activity (IC_50_ = 1.84 µM) slightly less than that of reference. From docking studies, it was evident that the potential ligands had a stronger binding affinity for both EGFR and HER2. The compound 100e was found to form three hydrogen bonds, one with Met769 and other two with Lys 721 residue of EGFR. Also, the compound 100b having a docking score of −10.62 Kcal mol^−1^ interacted with Ser 696 and Met 769 residues of EGFR.^52^

**Scheme 18:**
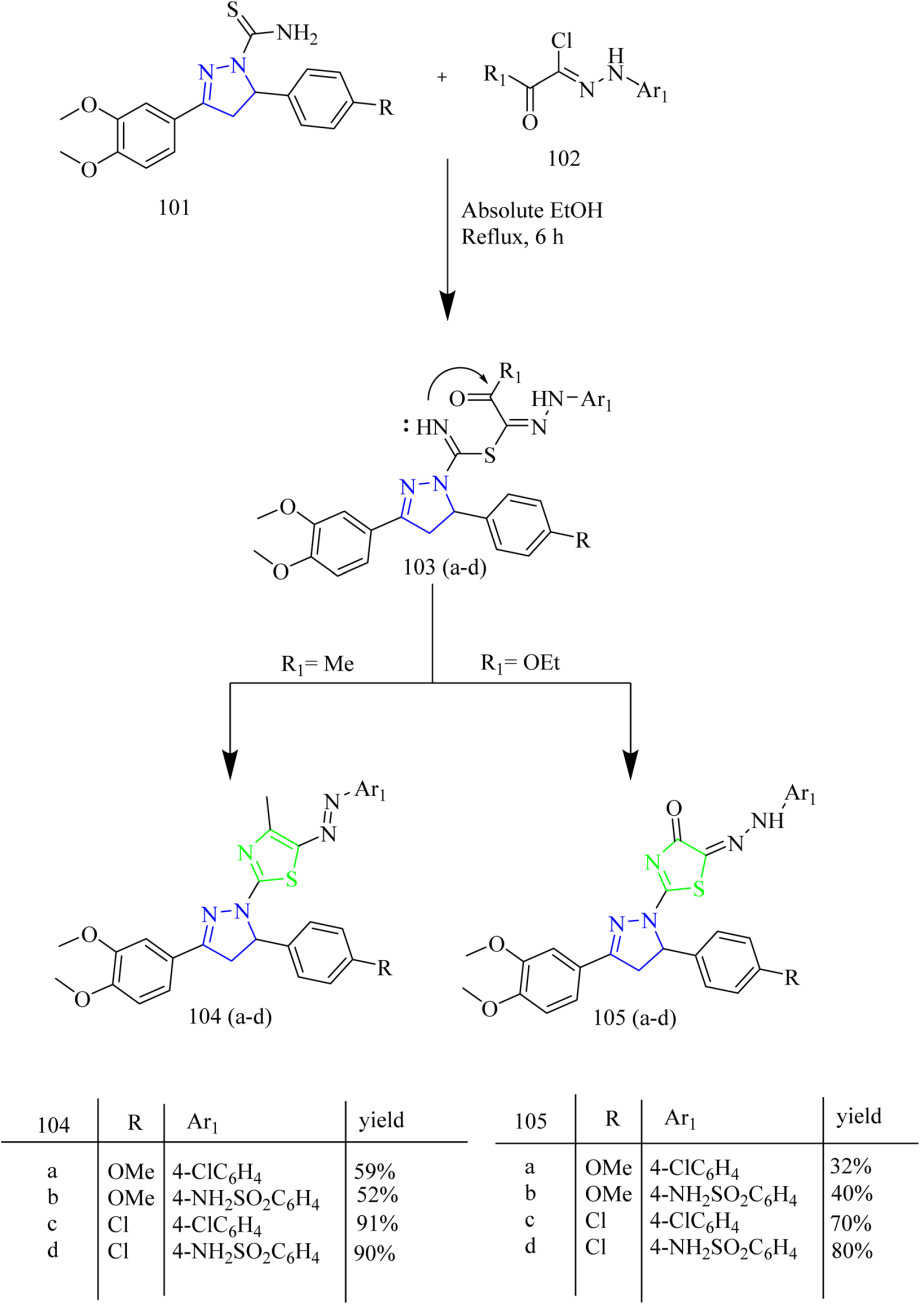
Synthesis of 104(a–d) and 105(a–d).

**Scheme 19:**
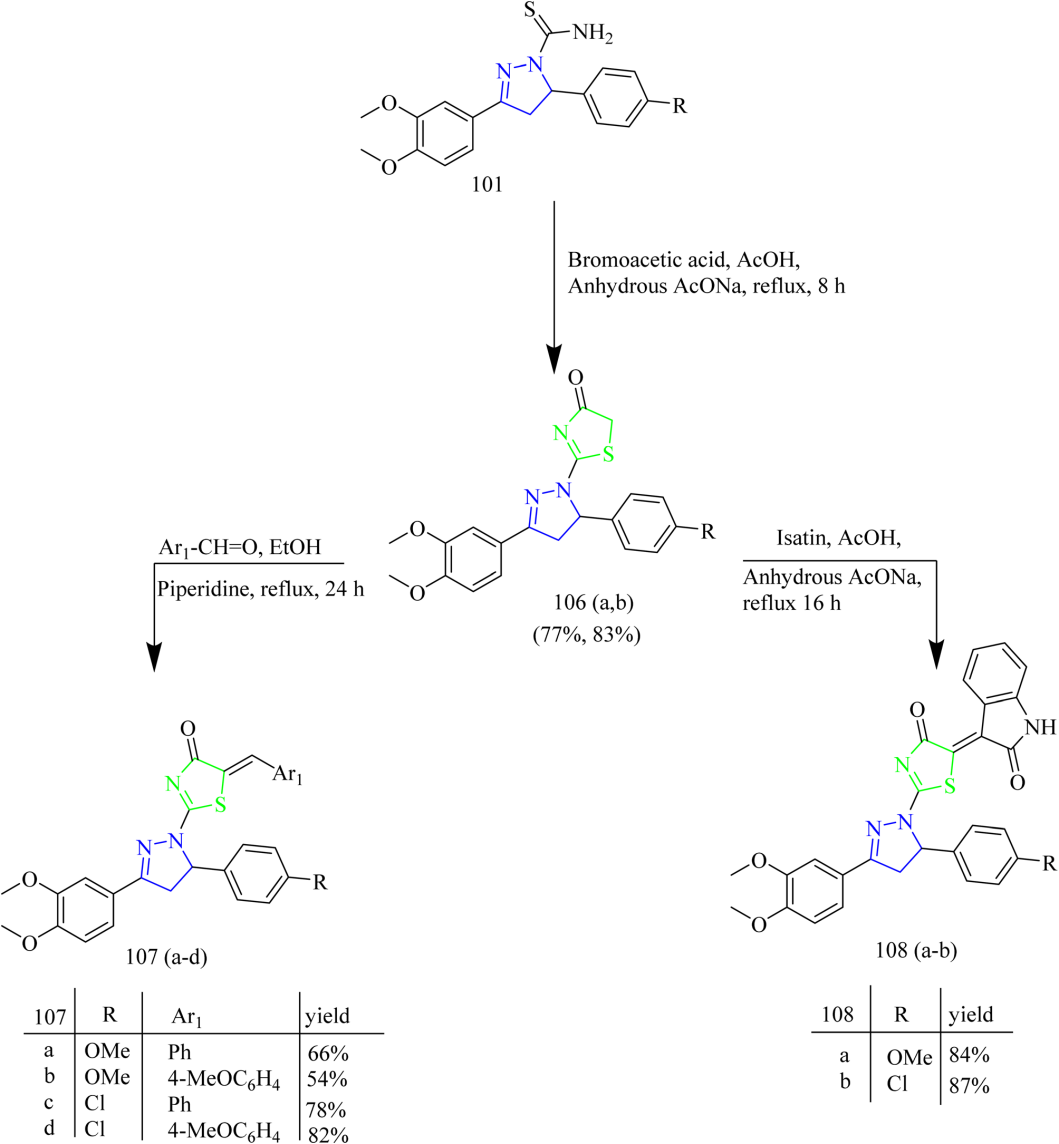
Synthesis of 107(a–d) and 108(a–d).

**Scheme 20:**
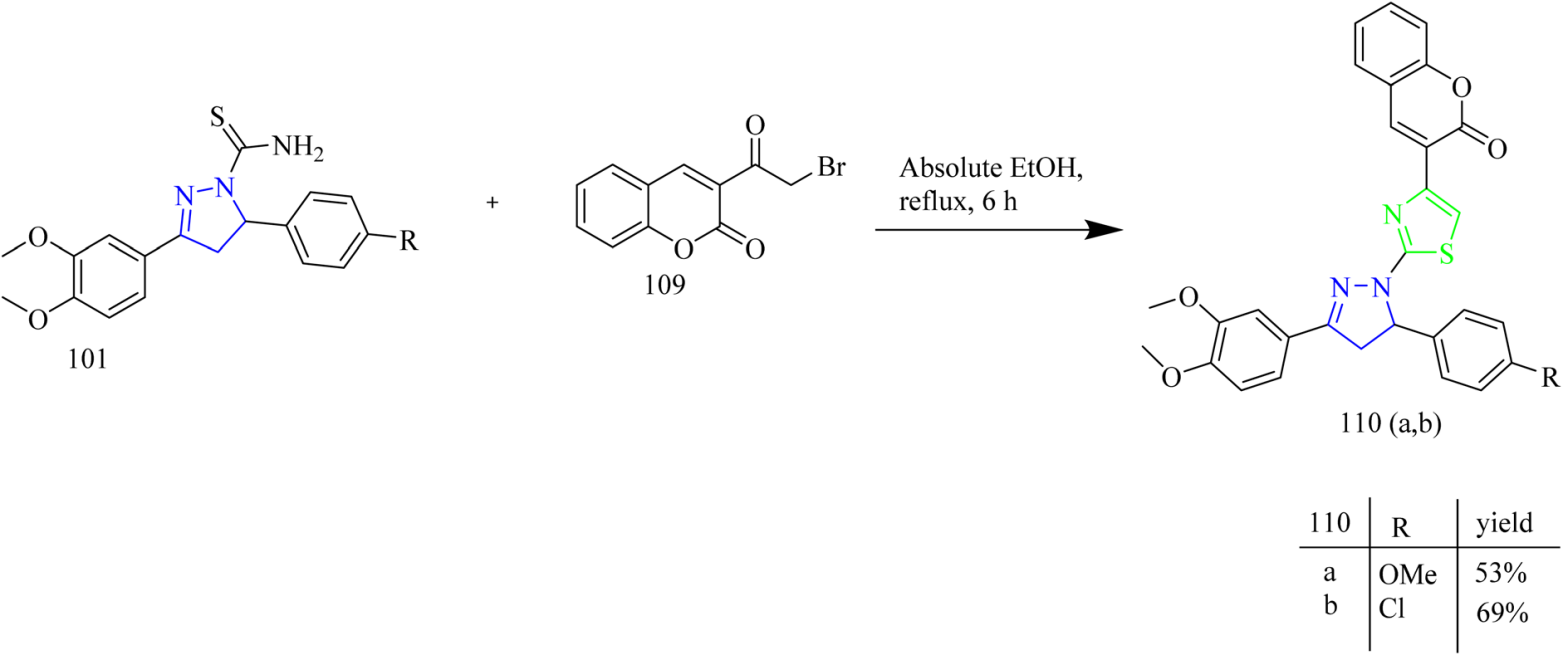
Synthesis of 110(a,b).

Fakhry *et al.*, reported the synthesis of thiazolyl-pyrazoline derivatives functioning as a dual inhibitor of EGFR and HER2. The carbothioamide derivatives 101 were refluxed with hydrazonoyl chloride derivatives 102 to form intermediates 103(a–d). Compounds 104(a–d) and 105(a–d) were synthesized from 103(a–d) through a cyclization reaction involving nucleophilic addition followed by dehydration with the elimination of water in case of 103(a,b) and loss of ethanol in case of 103(c,d) (Scheme 18). The carbothioamide derivatives were refluxed with bromoacetic acid to yield thiazolone intermediates 106(a,b) respectively, which were reacted with different aldehydes in presence of piperidine as a base, yielded compounds 107(a–d) (Scheme 19). Furthermore, thiazolone intermediates 106(a,b) were reacted with isatinin yielding compounds 108(a,b) (Scheme 19). Finally, the compounds 110(a,b) were synthesized *via* reaction of brominated acetyl coumarin 109 with carbothiamide intermediate 101 in EtOH under reflux condition (Scheme 20). When compared to standard lapatinib (IC_50_ = 5.88 µM), compounds 106a, 106b, 110a, and 110b showed strong anticancer activity against MCF-7 cell lines with IC_50_ values of 4.08, 5.64, 3.37 and 3.54 µM. Also, enzymatic tests were carried out to show the dual inhibitory activity of the most cytotoxic compounds 106a and 106b against EGFR and HER2. These compounds showed promising inhibitory potency against EGFR (IC_50_ = 0.024 and 0.026 µM, respectively) and HER2 (IC_50_ = 0.047 and 0.081 µM, respectively) compared to lapatinib (IC_50_ = 0.007 and 0.018 µM). Since the substitution on C5 reduces the potent activity of the thiazole, the compounds 106(a,b) and 110(a,b) were found to be more potent than the other intended compounds. Within the EGFR binding region, compound 110a formed two H-bonds with Cys797 amino acids in addition to two π–H bindings with Arg841 and hydrogen bonds with Met793, Lys745, and Val726. In case of HER2, compound 110a showed a π–H bond with the amino acid Val734, two H-bonds with the amino acids, Thr862, Asn850, and Asp863, and hydrogen bonds with Met801, Asn850, and Asp863. Compound 110a interacted within the EGFR binding site by forming π–H interactions with Val726, Thr854, and Arg841, along with two π–H bonds involving Asp855.^53^

**Scheme 21:**
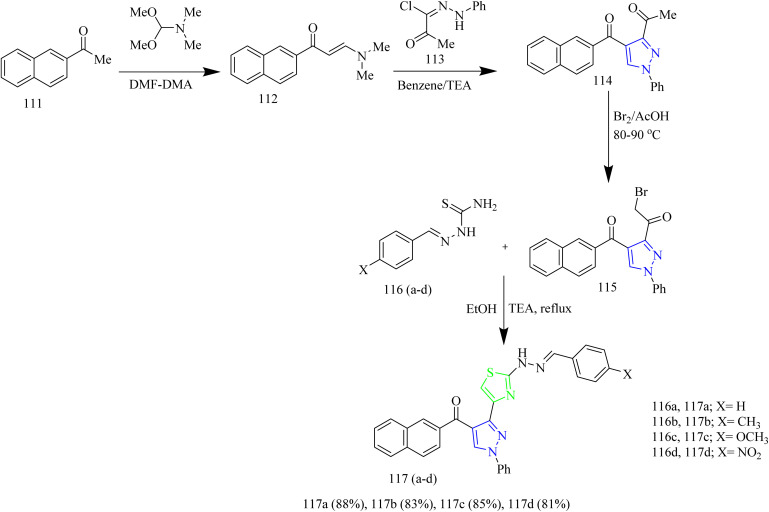
Synthesis of 117(a–d).

**Scheme 22:**
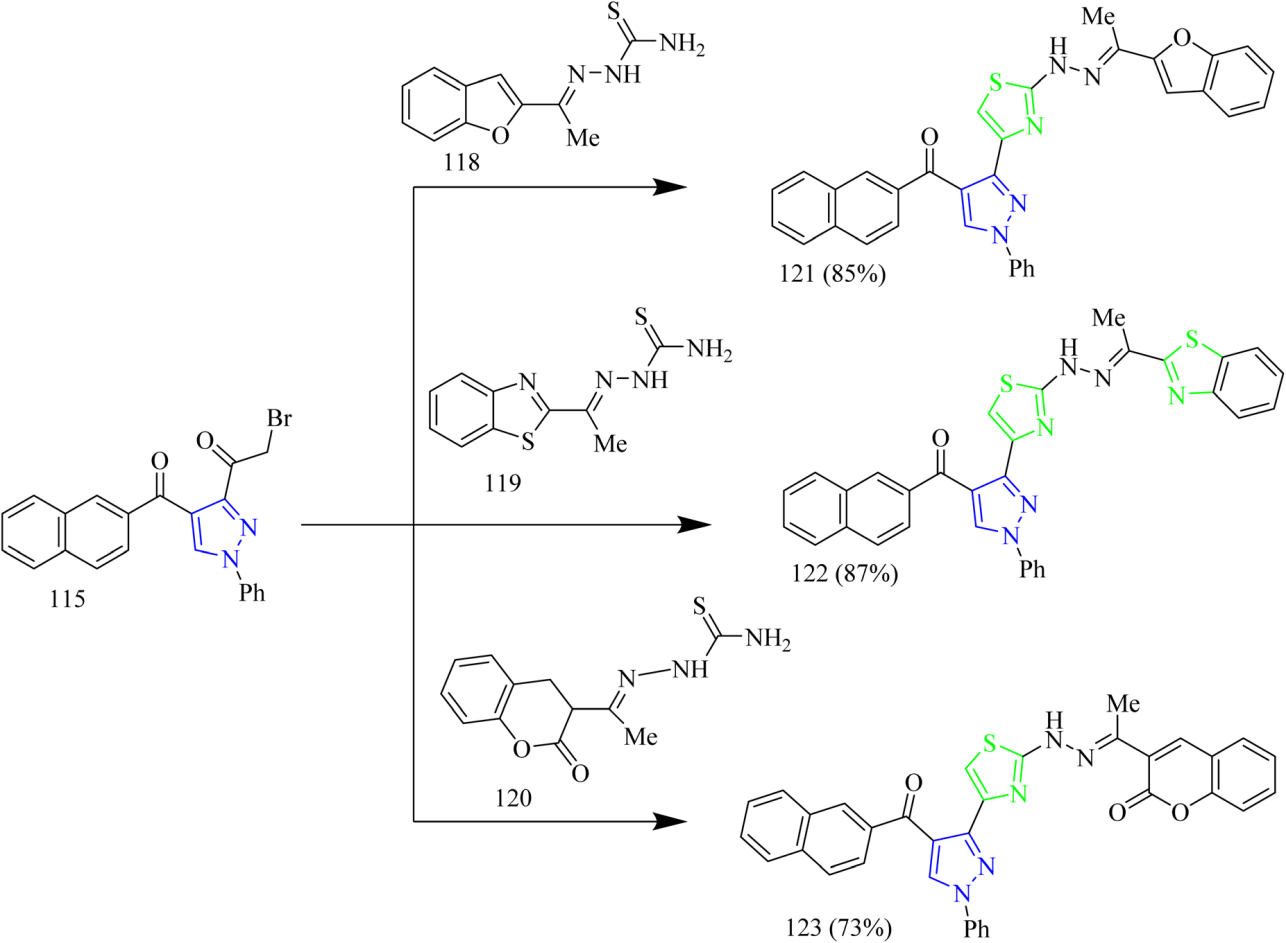
Synthesis of 121, 122, 123.

**Scheme 23:**
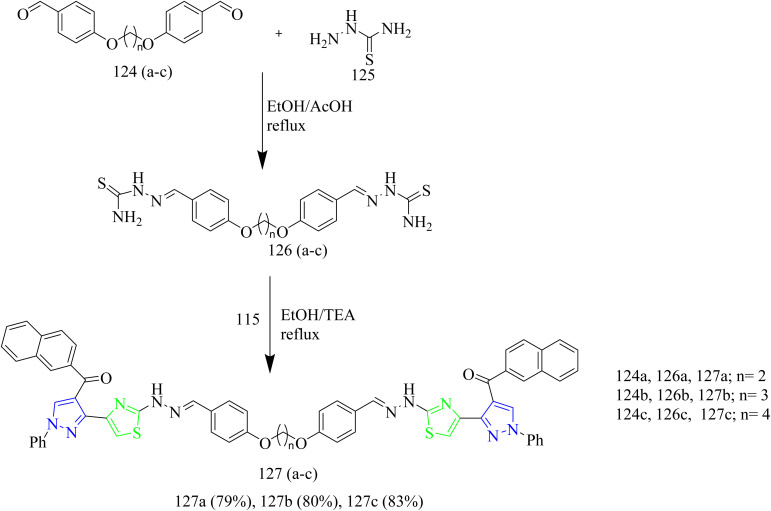
Synthesis of 127(a–c).

**Scheme 24:**
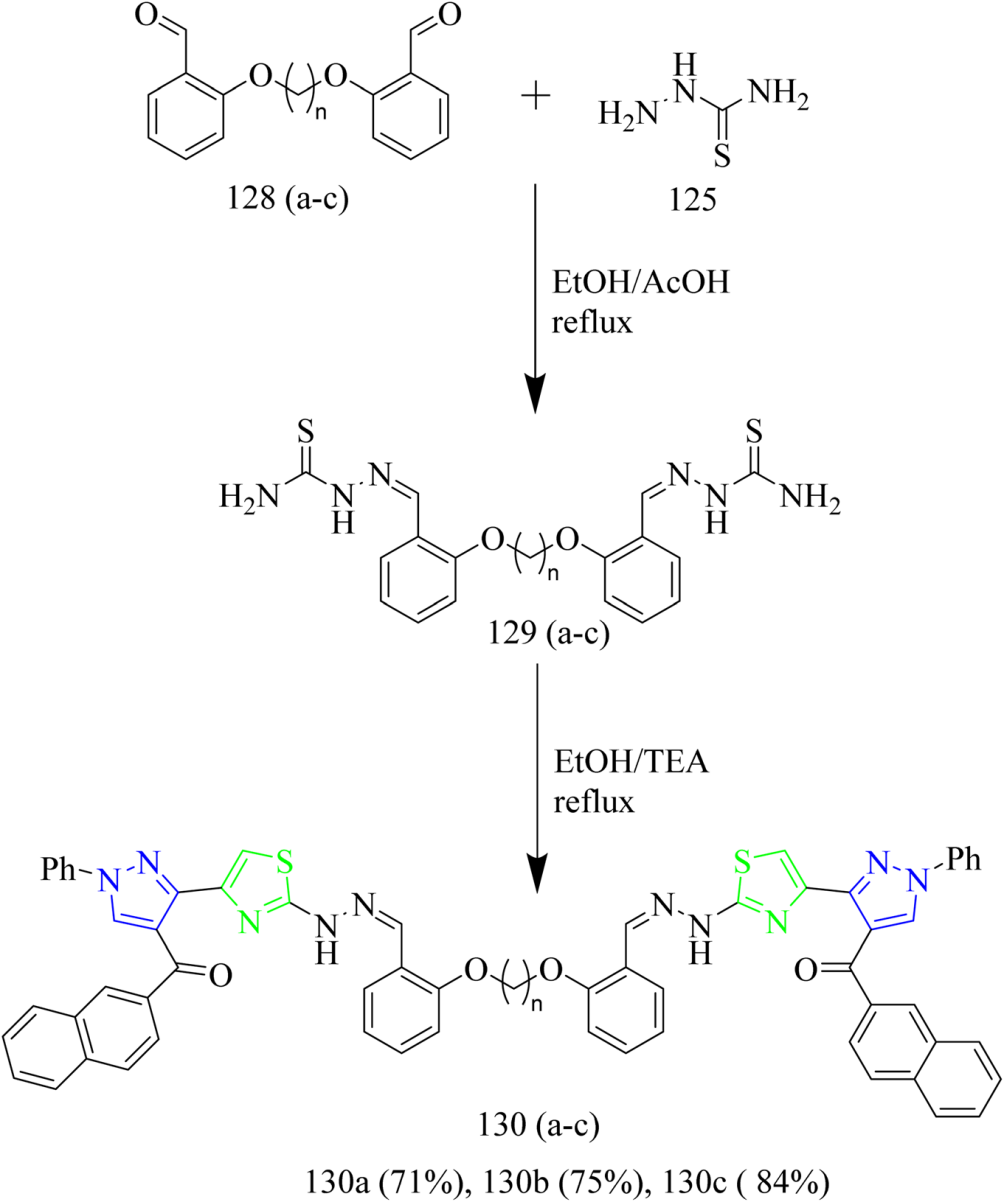
Synthesis of 130(a–c).

Salem and co-workers synthesized new mono and bis-thiazole pyrazolyl hybrid derivatives as anti-cancer agents. Using a series of procedures outlined below the potent molecule was synthesized. Intermediate 112 was synthesized by refluxing acetyl naphthalene 111 with DMF-DMA. 112 was then treated with 2-oxo-*N*′-phenylpropanehydrazonoyl chloride 113 in presence of TEA as a base under benzene refluxing yielded pyrazole intermediate 114 which was further subjected to bromination using bromine in acetic acid reagent at 80–90 °C to yield 3-bromocetylpyrazole intermediate 115. 115 was reacted with various aldehyde-thiosemicarbazones 116(a–d) under ethanol reflux in presence of triethylamine as a base afforded (pyrazol-3-yl)thiazole derivatives 117(a–d) (Scheme 21). Similarly, the reaction of 115 intermediate with various thiosemicarbazones 118, 119, 120 under same reaction conditions yielded thiazole–pyrazole hybrids 121, 122, 123 containing heterocyclic ring in their structures (Scheme 22). Next, bis-pyrazolylthiazoles containing alkyleneoxy-phenylene spacers were synthesized as follows. 124(a–c) and 128(a–c) were reacted with thiosemicarbazide 125 under ethanol reflux in presence of acetic acid to yield 126(a–c) and 129(a–c) (Scheme 23). The intermediates 126(a–c) and 129(a–c) were then reacted with 115 under ethanol reflux in presence of TEA yielded the final products 127(b,c) and 130a (Scheme 24). The compounds 127(a–c) and 130(a–c) were identified as the most potent compounds in the series with IC_50_ value of 0.97, 3.26 and 3.57 µM, respectively, in contrast to Lapatinib, the reference drug (IC_50_ = 7.45 µM). Similarly, when compared to lapatinib (IC_50_ = 6.1 and 17.2 nM), compound 127c showed promising EGFR and HER-1 inhibition activity with IC_50_ values of 4.98 and 9.85 nM, respectively. Remarkably, doubling the hybrid compounds into bisheterocycles led to an immense enhancement in the cytotoxic efficacy in comparison to simple molecular hybrids, where the cytotoxic potency increased by about eight times when the pharmacophoric scaffold was doubled. In case of 127c the IC_50_ was observed to be 0.97 µM and in case of 117c the IC_50_ value was 7.39 µM.^54^

**Scheme 25:**
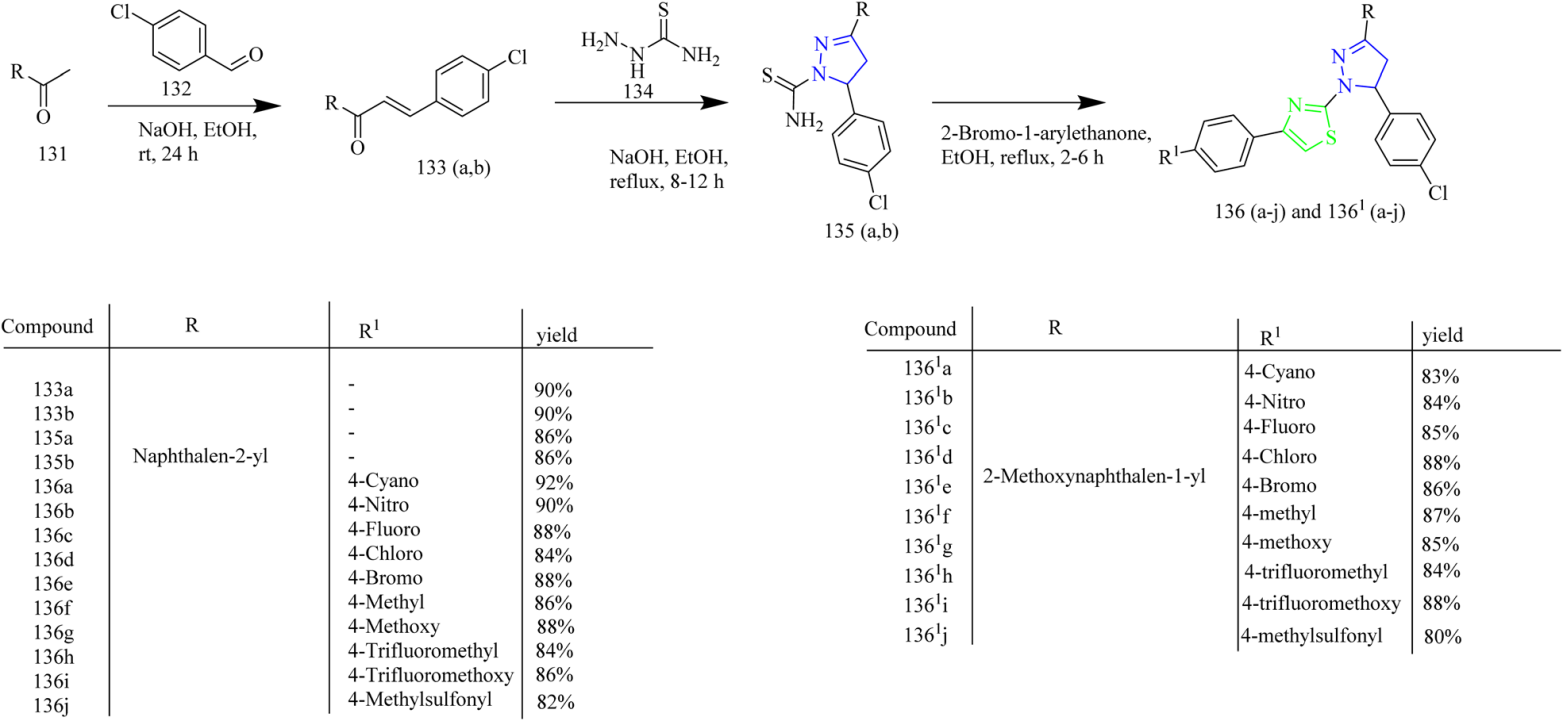
Synthesis of 136(a–j) and 136^1^(a–j).

Naphthalene linked thiazole–pyrazole hybrids as cancer inhibitors were designed and synthesized by CiFtci *et al.* The synthesis involved preparation of chalcone intermediate 133(a,b) by the reaction of 4-chlorobenzaldehyde 132 with ketone 131. The chalcone intermediate was then reacted with thiosemicarbazide 134 to yield a pyrazoline intermediate 135(a–b). The compounds 135a and 135b were heated with 2-bromo-1-arylethanone under reflux condition to yield compounds 136(a–j) and 136^1^(a–j) (Scheme 25). The antiproliferative activity of the synthesized compounds were tested on MCF-7 and A549 NSCLC cell lines. In contrast to lapatinib (IC_50_ = 16.44 ± 3.92 µM) the compound 132e caused significant toxicity in A549 c`ells, with an IC_50_ value of 9.51 ± 3.35 µM. At 10 µM concentration, the compound 136e demonstrated its selective kinase inhibition for EGFR at a rate of 58.32% and no discernible suppression of HER2. According to the molecular docking evaluation, the compound 136e exhibited high affinity in the ATP binding region of EGFR with a distinctive binding profile from lapatinib. The potent anticancer activity of the compound 136e was positively impacted by the 4-bromophenyl substitution at the pyrazoline thiazole core. The compounds 136(a–j) formed π-cation interactions with Lys721 *via* naphthyl moiety. Also 136^1^(a–j) showed no significant association with the EGFR active site. The compound 136e did not form an essential hydrogen bond with Met769 in the ATP binding region of EGFR TK, potentially indicating a decreased *in vitro* EGFR TK inhibitory activity compared to lapatinib.^55^

**Scheme 26:**
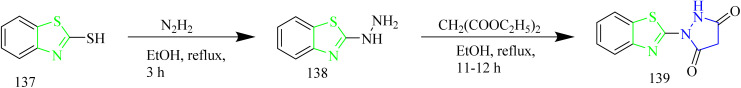
Synthesis of 139.

Mohammed *et al.* synthesized new benzothiazole derived pyrazolidine-dione and carried out *in vitro*, *in vivo*, and docking studies to evaluate the anticancer activity of the potent molecule. The synthetic scheme involved reaction between 2-mercaptobenzothiazole 137 with hydrazine under reflux condition using ethanol as solvent to yield a hydrazine-benzothiazole intermediate 138. The obtained intermediate 138 was then reacted with diethyl malonate to yield target compound benzothiazole–pyrazolidine-dione hybrid 139 (Scheme 26). The compound 139 demonstrated potent activity compared to compound 138. The cells lines used in *in vitro* study were SKG-T4 (oesophageal cancer), AMGM5 (glioblastoma), and REF (rat embryo fibroblasts). It was found that the compound 139 showed 97% inhibition at 6 µg mL^−1^ against SKG-T4 cells, 94% inhibition at 6 µg mL^−1^ against AMGM cells, and 45% inhibition at 6 µg mL^−1^ against REF cells. Docking studies were performed for the EGFR (PDB code: 8JFQ). The compound 139 was found to form hydrogen bonds with Arg836, Lys860, and Gly874 amino acid residues of EGFR. Further, the *in vivo* toxicity of the potent compound was studied. In mice the compound 139 showed LD_50_ value of 52.31 mg Kg^−1^ this indicates the acute toxicity of the compound.^56^

**Scheme 27:**
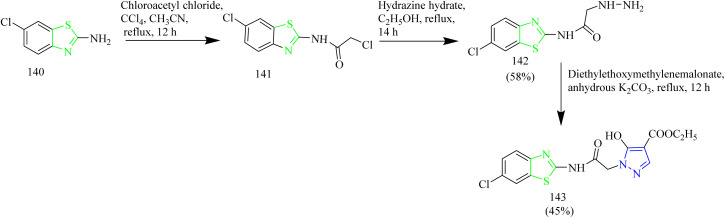
Synthesis of 143.

Gabr and co-workers synthesized acetamidobenzothiazole–pyrazole hybrids as EGFR kinase inhibitors. The synthesis involved acylation of amino-chloro-benzothiazole 140 with chloroacetyl chloride in presence of CCl_4_ to yield an acetamide intermediate 141, which was then reacted with hydrazine hydrate at reflux temperature to yield hydrazinyl acetamide intermediate 142. Finally, the intermediate 142 was reacted with diethyl ethoxymethylenemalonate using ACN as solvent under reflux condition yielded the final product 143 (Scheme 27). The synthesized compound 143 was tested for anticancer activity against breast, colon, and NSCLC. The compound 143 showed GI_50_ value of 0.317, 0.353, and 0.0573 µM against the above-mentioned cell lines. Further the compound 143 was subjected to additional testing against EGFR kinase. A 10-dose IC_50_ mode using threefold dilution steps from a concentration of 20 mM was used against staurosporine 24–26 as a control for EGFR kinase. The IC_50_ value of 0.239 µM was observed in case of compound 143 whereas, staurosporine exhibited IC_50_ value of 0.0533 µM which is a non-selective kinase inhibitor. The remarkable selectivity of the compound 143 for EGFR could be due to the variation in the geometry of the binding pocket of the enzyme which allows the proper fitting and interaction of the compound 143.^57^

Across different studies, thiazole–pyrazole hybrid inhibitors have shown several shared structural traits that explain their strong biological activity. The thiazole ring acts the core pharmacophore, forming π–π and hydrogen bond interactions with key hinge residues of EGFR, such as Met793 and Lys721. When linked to a pyrazole or pyrazoline ring, the system becomes more planar and electronically delocalized, allowing it to fit more effectively within the ATP-binding pocket. Substituents like methyl or methoxy groups in 8g, 50b, and 62e enhance hydrophobic interactions, while halogen atoms such as chlorine, fluorine or cyano groups in 18k, 23f, 95g, and 100g strengthen binding through dipole and halogen-bond interactions. Bulky aromatic or hydrophobic fragments like naphthalene 136e or adamantane 13e further stabilize the complex and improve membrane penetration. Moreover, linkers such as hydrazone and amide groups in 35a and 143 help establish additional hydrogen bonds and maintain an optimal molecular conformation, together defining the key structural features behind the potency of thiazole-based EGFR inhibitors.

### Thiazole–quinoline hybrids

**Scheme 28:**
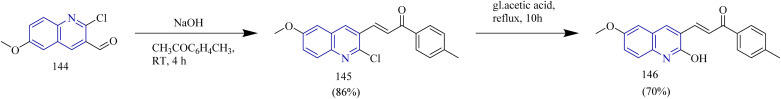
Synthesis of 146.

**Scheme 29:**
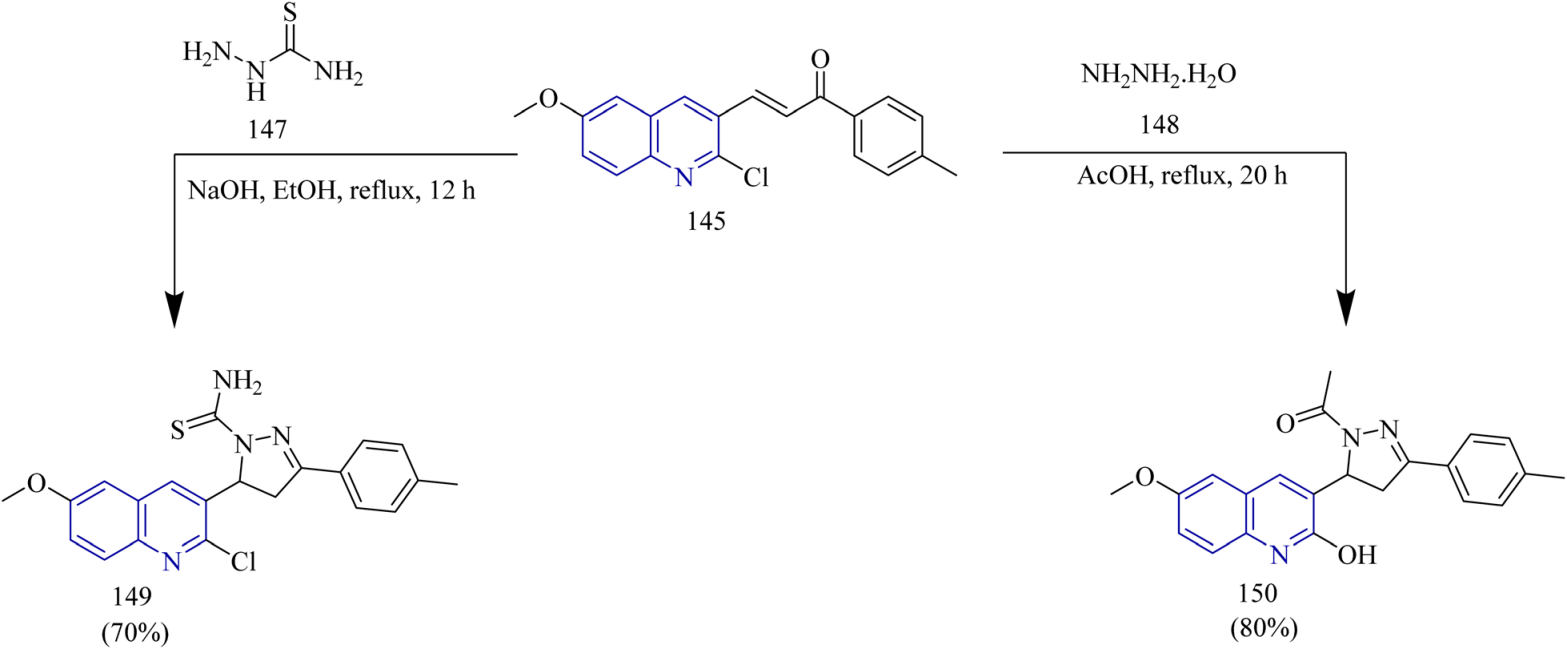
Synthesis of 149 and 150.

**Scheme 30:**
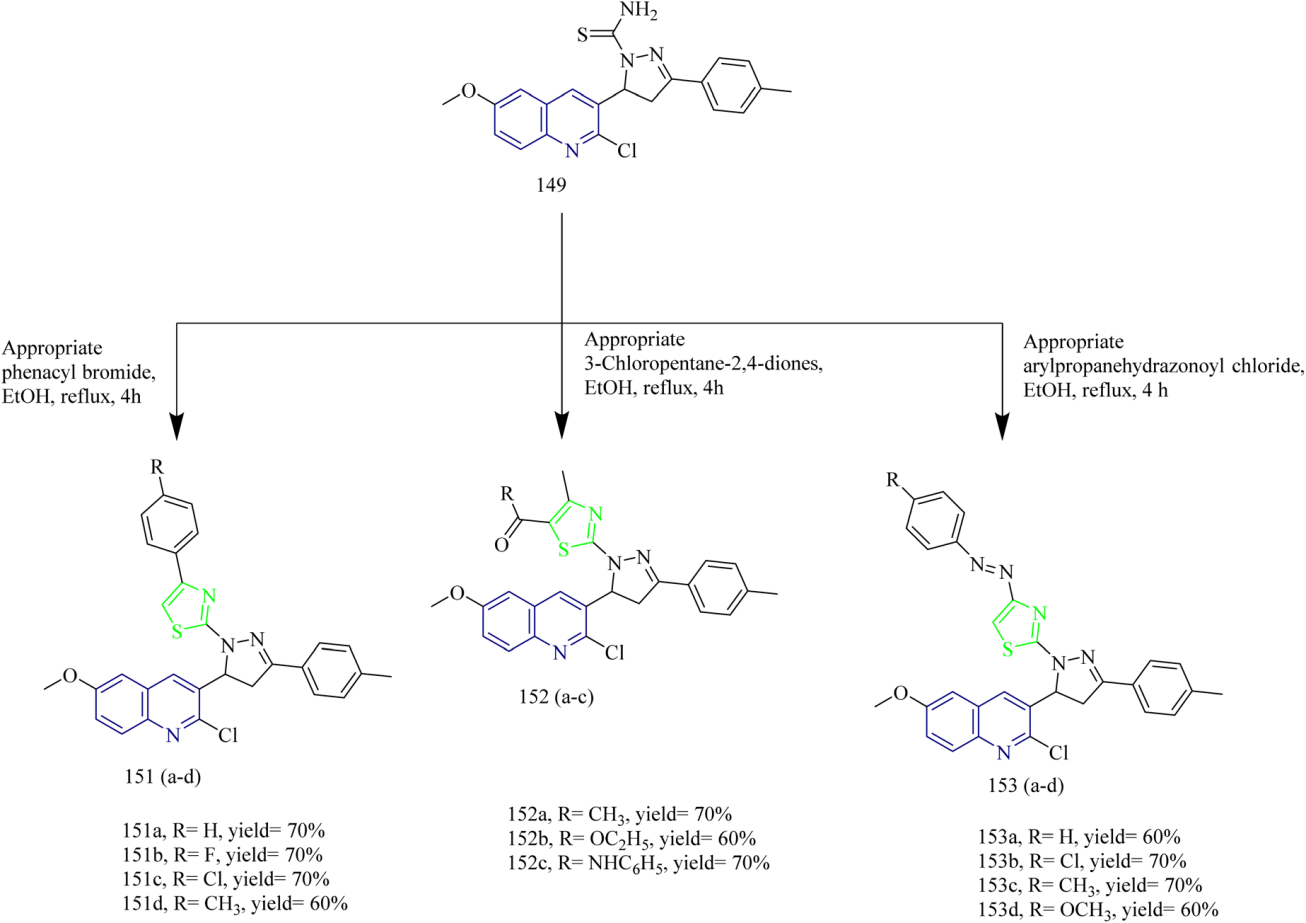
Synthesis of 151(a–d), 152(a–d), and 153(a–d).

Quinoline based thiazole–pyrazole hybrids (Table 2) were synthesized by George *et al.* The synthetic pathway involves condensation of aldehyde 144 with methyl acetophenone yielded a chalcone 145. The intermediate 145 was then subjected to conversion into hydroxy analogue 146*via* refluxing in glacial acetic acid (Scheme 28). In addition to this, the chalcone intermediate 145 was subjected to cyclisation using thiosemicarbazide 147 to yield pyrazoline intermediate 149. Cyclocondensation of chalcone intermediate with hydrazine hydrate 148 yielded an acetyl pyrazoline compound 150 (Scheme 29). Moreover, the pyrazoline intermediate were reacted with appropriate 1-aryl-2- bromoethanones to afford thiazole pyrazole hybrids 151(a–d) (Scheme 30). Similarly, reaction of pyrazoline intermediate with 3-chloropentane-2,4-diones gave methyl substituted thizole-pyrazole-quinolines 152(a–c) (Scheme 30). Finally, dihydropyrazole-thiazole-quinoline 153(a–d) were synthesized by refluxing the pyrazoline derivative with 2-oxo-*N*-arylpropanehydrazonoyl chlorides (Scheme 30). In MCF-7 cell lines the compound 150b showed the most significant activity, with an IC_50_ value of 0.038 µM. In case of HeLa cell lines, the compounds 153(a–d) exhibited promising cytotoxic activity in the IC_50_ value range of 0.120–1.166 µM. Among these the 4-chlorophenyldiazene derivative 153b had the highest activity with IC_50_ value of 0.12 µM. Lastly, when compared to the reference CHS 828 (IC_50_ = 2.315 µM) the compound 149c demonstrated greater anticancer activity (IC_50_ = 1.652 and 0.064 µM) when tested against DLD1 colon cell lines. Compounds 145, 146, 150, 151a, 151b, 152b, 152c, and 153a exhibited remarkable cytotoxicity against DLD1 cells while being safe in case of normal fibroblasts and were found to inhibit EGFR with IC_50_ range of 0.064–1.277 µM. The compounds 145, 151b, and 152c displayed high to good effectiveness as EGFR inhibitor at nanomolar concentrations (IC_50_ = 37.07, 31.8, and 42.52 nM, respectively), in contrast to gefitinib (IC_50_ = 29.16 nM). Furthermore, these compounds were docked against EGFR proteins and the docking scores were found to be −11.32, −12.45, −12.94 kcal mol^−1^ which was close to the docking score of Gefitinib (−11.26 kcal mol^−1^).^58^

**Table 2:** Overview of EGFR inhibition activity of thiazole–quinoline hybrids

Compound	Key substituent	Major interactions	EGFR IC_50_ (µM)	SAR observation
151b	4-Fluorophenyl moiety	H-bond with Met769 and π-cation interaction with Lys721	31.8 nM	Electron withdrawing groups enhanced lipophilicity
158b	Carbothioamide moiety with 2-chloro-6-methoxy-quinoline	H-bond with Met769, π-cation interaction with Lys721, hydrophobic bond with Leu694, Val702, and Ala719	28.77 nM	Hydrophobic substituent enhanced cell permeability
166c	Pyrimidine thiazole fused system	π–π stacking with Phe699, Leu694	—	Aryl substituent on the thiazole ring enhanced binding strength
170f, 170i	4-Fluoro phenyl, 4-trifluoromethyl substitution	H-bond with Cys781, Gly857, and Thr903	2.17 nM	Trifluoromethyl substitution improves hydrophobic filling increasing electron-deficient character of phenyl ring
173e	Methoxy substitution on quinoline ring	H bond with Met769 and Asp831	0.249	Electron donating substituents increased electron density and improved binding affinity

**Scheme 31:**
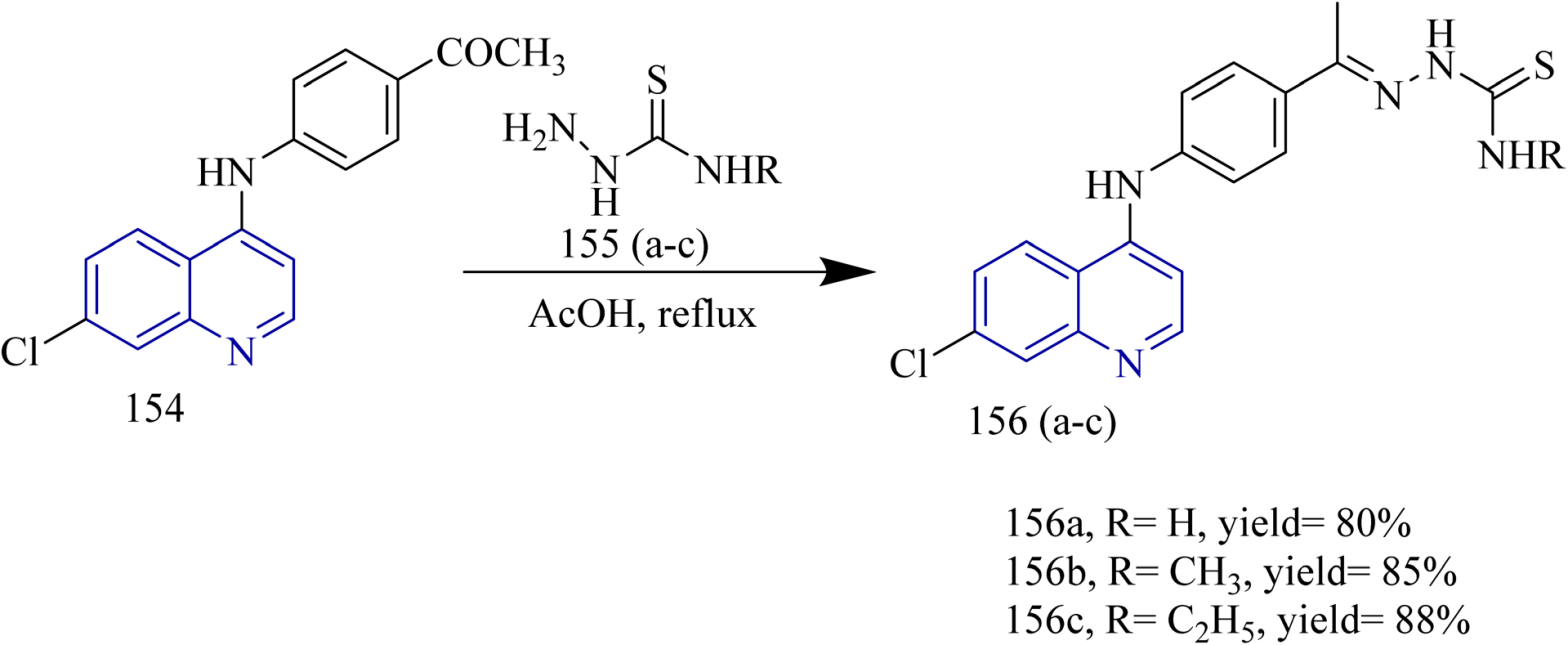
Synthesis of 152(a–c).

**Scheme 32:**
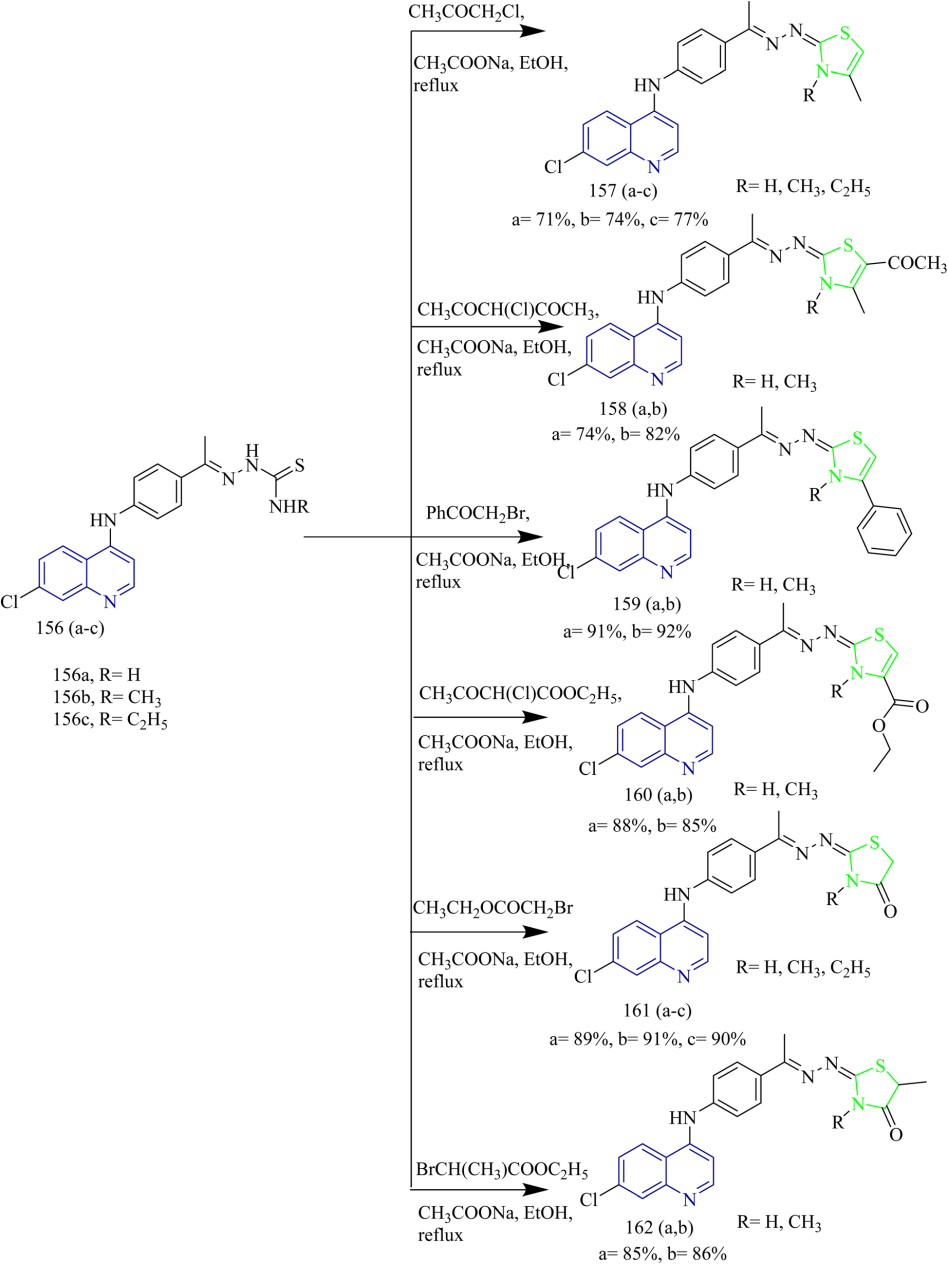
Synthesis of 157(a,c), 158(a,b), 159(a,b), 160(a,b), 161(a–c), 162(a,b).

Quinoline based derivatives containing thiazoles targeting the EGFR signalling pathway was designed and synthesized by Batran and co-workers. The synthesis involved the reaction of quinoline ketone 154 with various thiosemicarbazide derivatives 155(a–c) in presence of ethanol as solvent under reflux condition to yield respective thiosemicarbazones 156(a–c) (Scheme 31). To these thiosemicarbazones suitable α-halocarbonyl compounds were added to yield thiazoline derivatives 157(a–c), 158(a,b), 159(a,b), 160 (a,b) (Scheme 32). Also, the thiosemicarbazones were 156(a–c) were reacted with ethylbromoacetate or ethylbrormopropionate leading to the formation of thiazolidinone derivatives 161(a–c), 162(a,b) (Scheme 32). Compared to lapatinib (IC_50_ = 4.69 µM), compounds 158b and 160b had the highest antiproliferative activity, with IC_50_ values of 33.19 and 5.35 µM, respectively. Even though 160b had higher cytotoxicity, the compound 158b was found to be better at inhibiting EGFR pathway which led to significant reduction in EGFR activity with IC_50_ values of 76.43 and 28.77 nM for 160b and 158b. Compounds 158b and 160b demonstrated significant binding interactions with the amino acids in the EGFR active sites with docking scores of −13.6959 and −14.4384 kcal mol^−1^, respectively, whose value was close to that of standard drug lapatinib (−15.5599 kcal mol^−1^).^59^

**Scheme 33:**
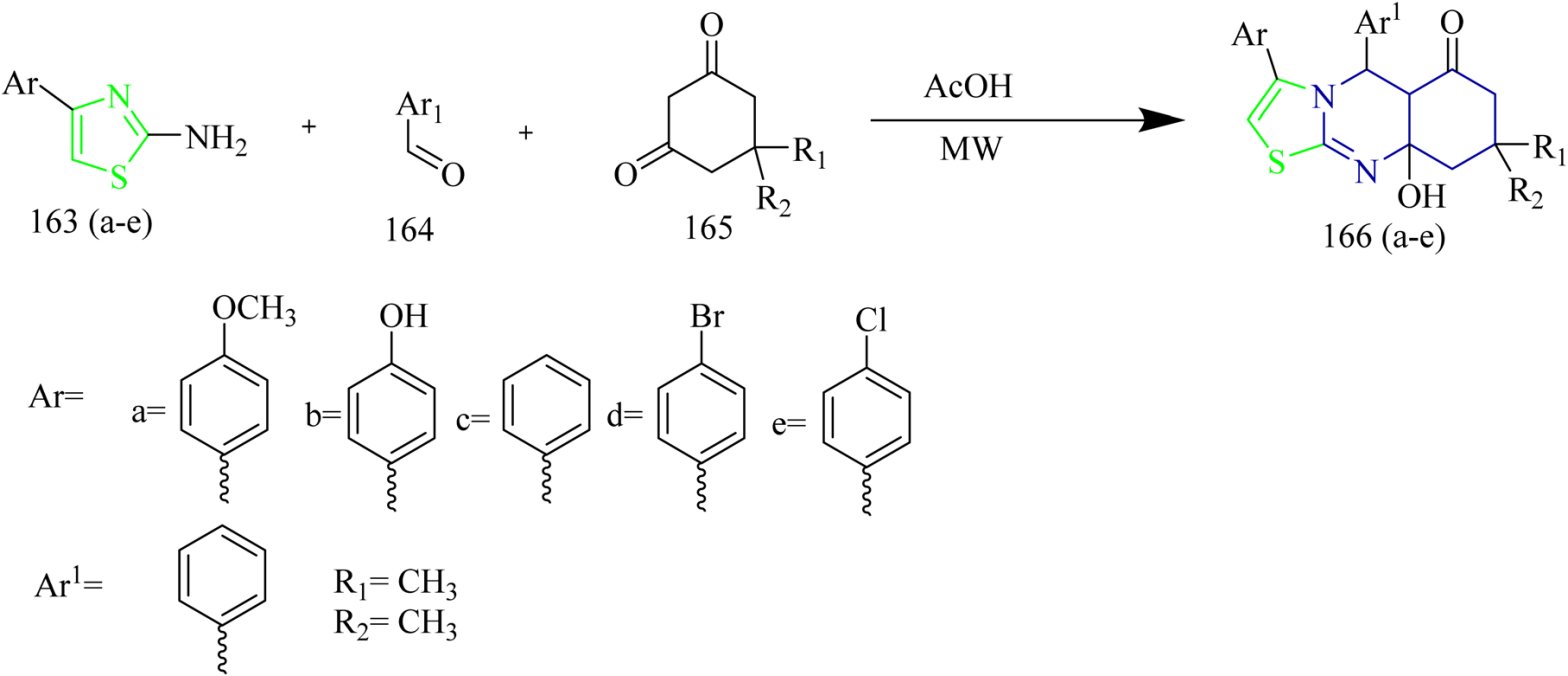
Synthesis of 166(a–e).

Ahmad Mir and colleagues calculated the free BE of thiazole–quinoline hybrids against EGFR-TKD and evaluated their anticancer efficacy using molecular dynamic modelling. Thiazolo–quinazolinones hybrids 166(a–e) were synthesized using multi-domino reactions (MDR) between substituted amino thiazole 163(a–e), a ketone 164, and a dione 165 with the help of a microwave synthesizer (Scheme 33). The compound 166b exhibited stronger inhibitory activity against MCF-7 cell lines with an IC_50_ value of 21.1 ± 0.7623 µM, while compound 166c displayed greater potency against Hep-G2 with an IC_50_ value of 13.8 ± 0.06152 µM. Molecular docking was used to assess the BE of thiazole–quniloline hybrids against EGFR-TKD, and the results were compared with erlotinib, a positive control. According to molecular docking the synthesized compounds 166(a–e) showed greater binding score ranging from 8.13 ± 0.0115 to 8.89 ± 0.0173 kcal mol^−1^. This is higher than that of noscapine (7.31 ± 0.5211 kcal mol^−1^) and erlotinib (7.54 ± 0.1411).^60^

**Scheme 34:**
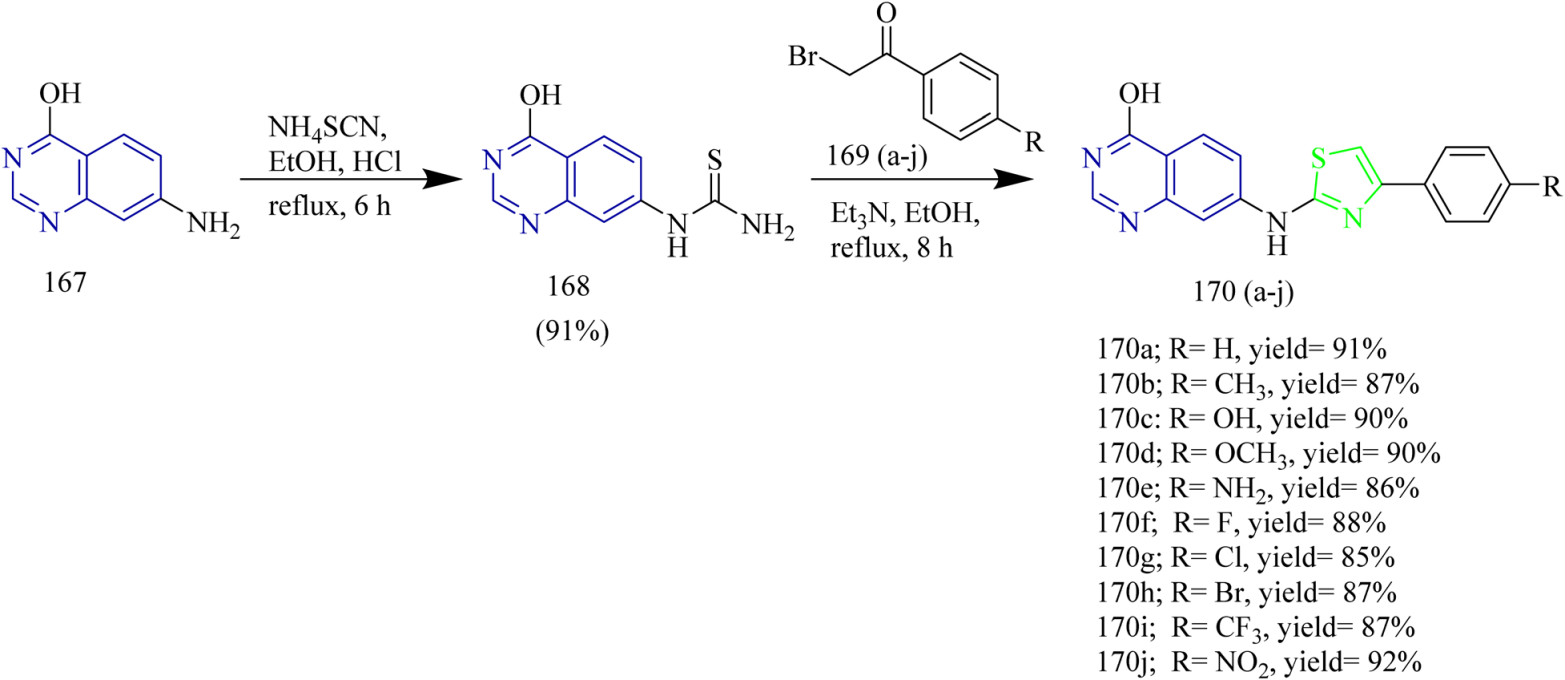
Synthesis of 170(a–j).

New range of quinazoline based thiazole hybrids as EGFR kinase inhibitors were designed and synthesized by Raghu and co-workers. The synthesis involved dropwise addition of HCl to a solution of 7-aminoquinazolin-4-ol 167 followed by addition of ammonium thiocyanate using ethanol as solvent to afford an intermediate 168. The obtained intermediate 168 was reacted with various phenacyl bromides 169(a–j) under reflux condition using ethanol as a solvent yielded the target quinazoline based thiazole hybrids 170(a–j) (Scheme 34). The compound 170i showed IC_50_ value of 2.86 ± 0.31, 5.91 ± 0.45, 14.79 ± 1.03 µM against MCF-7, HepG2, and A549 cell lines. Compound 170f demonstrated potent inhibitory activity, showing IC_50_ values of 2.17, 2.81, and 3.62 nM against wild type EGFR, the L858R/T790M double mutant, and the L858R/T790M/C797S triple mutant, respectively. Docking studies revealed that molecules 170i and 170j exhibited higher docking scores (−8.49 and −8.46 kcal mol^−1^) than the commonly prescribed drug Osimertinib, which has a docking score of −8.21 kcal mol^−1^.^61^

**Scheme 35:**
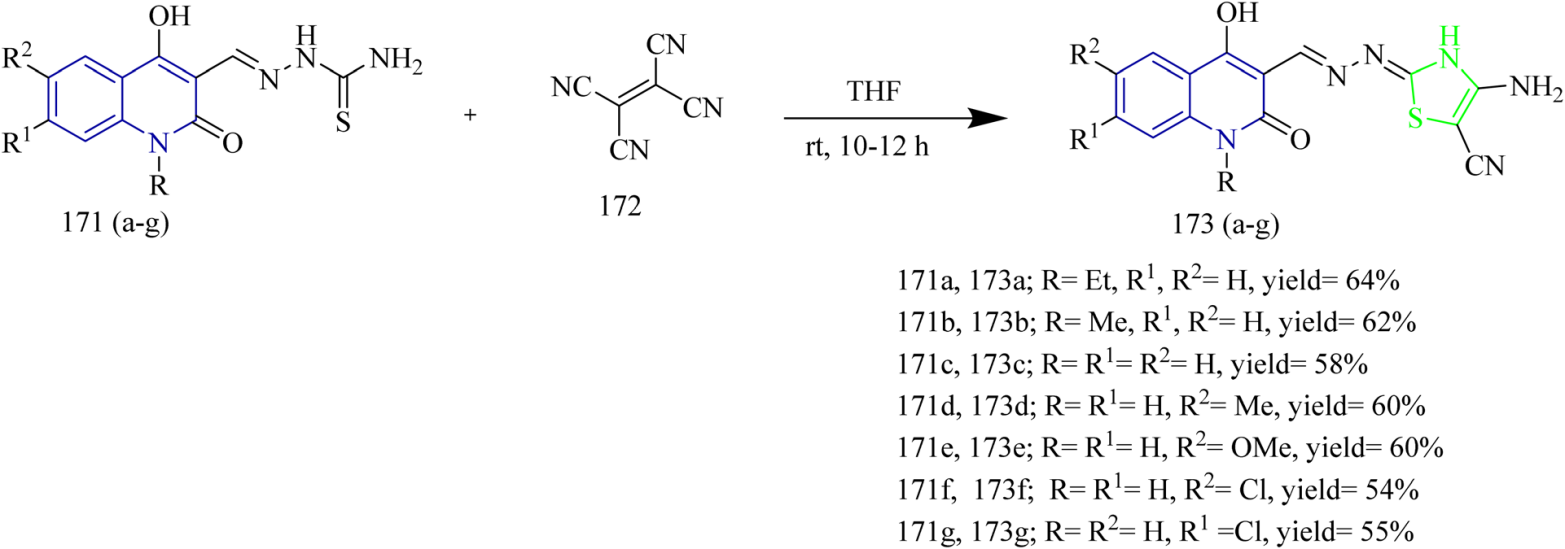
Synthesis of 173(a–g).

Mohamed *et al.* synthesized new thiazole–quinolone hybrids as EGFR inhibitors. The final product 173(a–g) was formed through a single step reaction between carbothioamide derivatives 171(a–g) and ethene-1,2,3,4-tetracarbonitrile 172 using THF as a solvent (Scheme 35). It was found that the lipophilic substitution of a chlorine atom at the 6-position of the quinoline moiety in 168f demonstrated enhanced anticancer activity against the A549 cell line, with an IC_50_ value of 7 ± 0.3 µM. This is 3.4 times more active than sorafenib (IC_50_ = 24 ± 4.52 µM). In Caco-2 cells, compounds 173b and 173d demonstrated strong anticancer effects, with IC_50_ values of 8 ± 6.49 µM and 9 ± 1.32 µM, respectively, in comparison to the reference drug sorafenib, which had an IC_50_ value of 6 ± 0.75 µM. Furthermore, the compounds 173a, 173b, and 173(e–g) were tested for their activity against EGFR pathway. It was found that the compound 173e (0.249 ± 0.008 µM) was more potent inhibitor, followed by compound 168a (0.440 ± 0.169 µM) and they exhibited comparable activity to gefitinib (0.087 ± 0.004 µM). Docking results illustrated that the compound 173e was found to fit into the pocket of EGFR protein with a docking energy of −31.35 kJ mol^−1^. Hence the synthesized compounds may be considered as viable options for the treatment of cancer.^62^

Thiazole–quinoline hybrids show potent EGFR inhibition by forming key interactions, such as hydrogen bonds with Met769 and π–π stacking with Phe699. The thiazole moiety enhances hydrophobic interactions, while the quinoline ring stabilizes binding in the ATP pocket. Electron-withdrawing or donating substituents improve lipophilicity and electron density, optimizing binding affinity. As a result, these hybrids demonstrate strong inhibitory activity, with sub-micromolar IC_50_ values. Compounds with electron-withdrawing groups, such as the 4-fluorophenyl unit in 147b, display better lipophilicity and stronger hinge binding with Met769 and Lys721. Compound 158b benefits from both hydrogen bonding and hydrophobic contacts due to its carbothioamide and 2-chloro-6-methoxy quinoline structure. The fused pyrimidine–thiazole system in 166c gains stronger binding through enhanced π–π stacking with Phe699 and Leu694. Similarly, 170f and 170i show that fluoro and trifluoromethyl groups boost potency by increasing electron deficiency and hydrophobic pocket fit. In contrast, 173e highlights how an electron-donating methoxy group can raise electron density and reinforce hydrogen bonding with Met769 and Asp831.

### Thiazole–imidazole hybrids

**Scheme 36:**
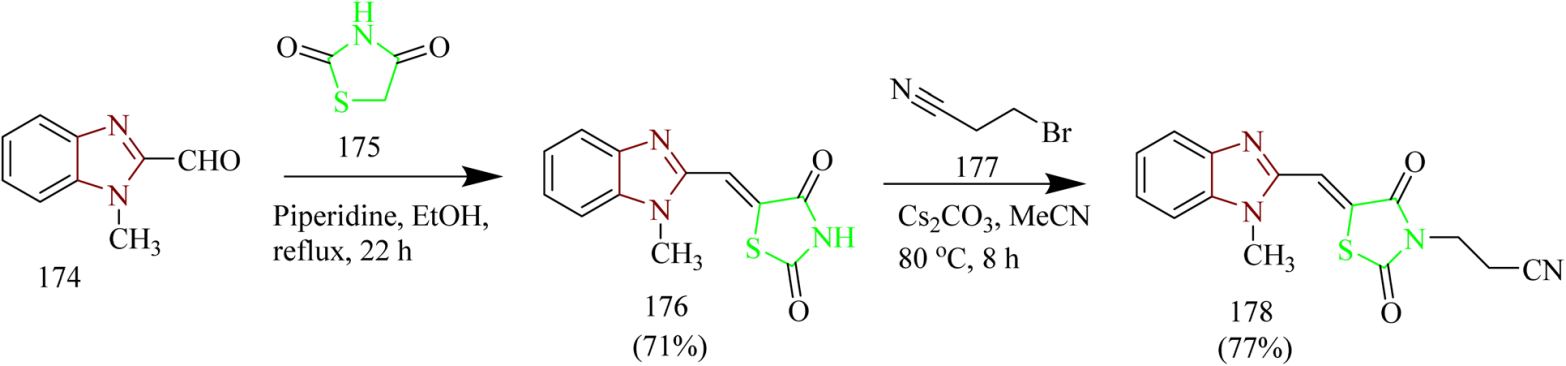
Synthesis of 178.

**Scheme 37:**
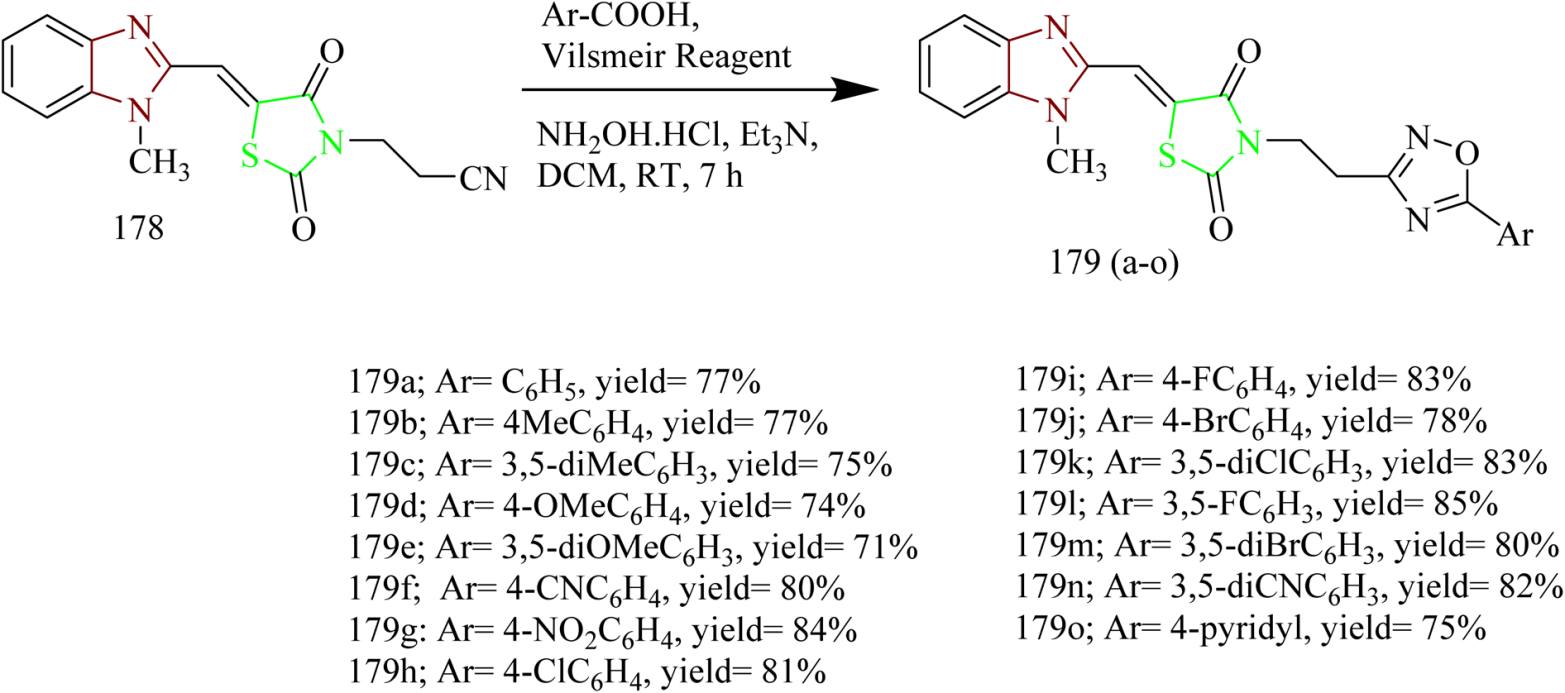
Synthesis of 179(a–o).

Venu and coworkers synthesized thiazolidine-2,4 dione, benzimidazole, and 1,2,4-oxadiazole hybrids functioning as EGFR inhibitors. The synthesis involved Knoevenagel condensation between benzimidazole-2-carbaldehyde 174 and thiazolidine-2,4-dione 175 to yield an imidazole-2-yl-thiazole hybrid intermediate 176. To this intermediate 176, 3-bromopropionitrile 177 was added to yield a nitrile intermediate 178 (Scheme 36). Finally, the nitrile intermediate 178 was subjected to reaction with various aromatic acids in presence of NH_2_OH.HCl and triethylamine in DCM solvent to afford series of hybrid molecules containing thiazolidine-2,4 dione, benzimidazole, and 1,2,4-oxadiazole 179(a–o) (Scheme 37). *In vitro* cytotoxic studies showed that the compounds 179d, 179e, 179f, and 179n are found to be effective against all cell lines. Compounds 179d with a 4-methoxy substituent and 179e with a 3,5-dimethoxy substituent showed greater efficacy against MCF-7 with an IC_50_ value of 1.56 ± 0.04 and 1.02 ± 0.009 µM which is greater than Erlotinib (IC_50_ = 4.15 ± 0.12 µM). On the other hand, compounds with different mono-electron withdrawing groups on the phenyl ring were less potent than compound 179f (IC_50_ = 2.17 ± 0.1 µM against MCF-7, which had a 4-CN substituent on the phenyl ring. Compound 179n with a 3,5-di-CN group on the phenyl ring outperformed standard drug erlotinib against all cell lines with IC_50_ values of 1.32 ± 0.05, 19.72 ± 0.67, and 11.27 ± 0.54 µM. The potent compounds 179d, 179e, 179f, and 179n were further checked for their EGFR inhibitory activity. The results showed that compound 179e had the highest BE -10.17 kcal mol^−1^ and inhibition constant (0.26 µM). It had formed two hydrogen bonds with the target protein, specifically involving Cys773 and Asp831, both exhibiting a bond length of 2.09 A°.^63^

**Scheme 38:**
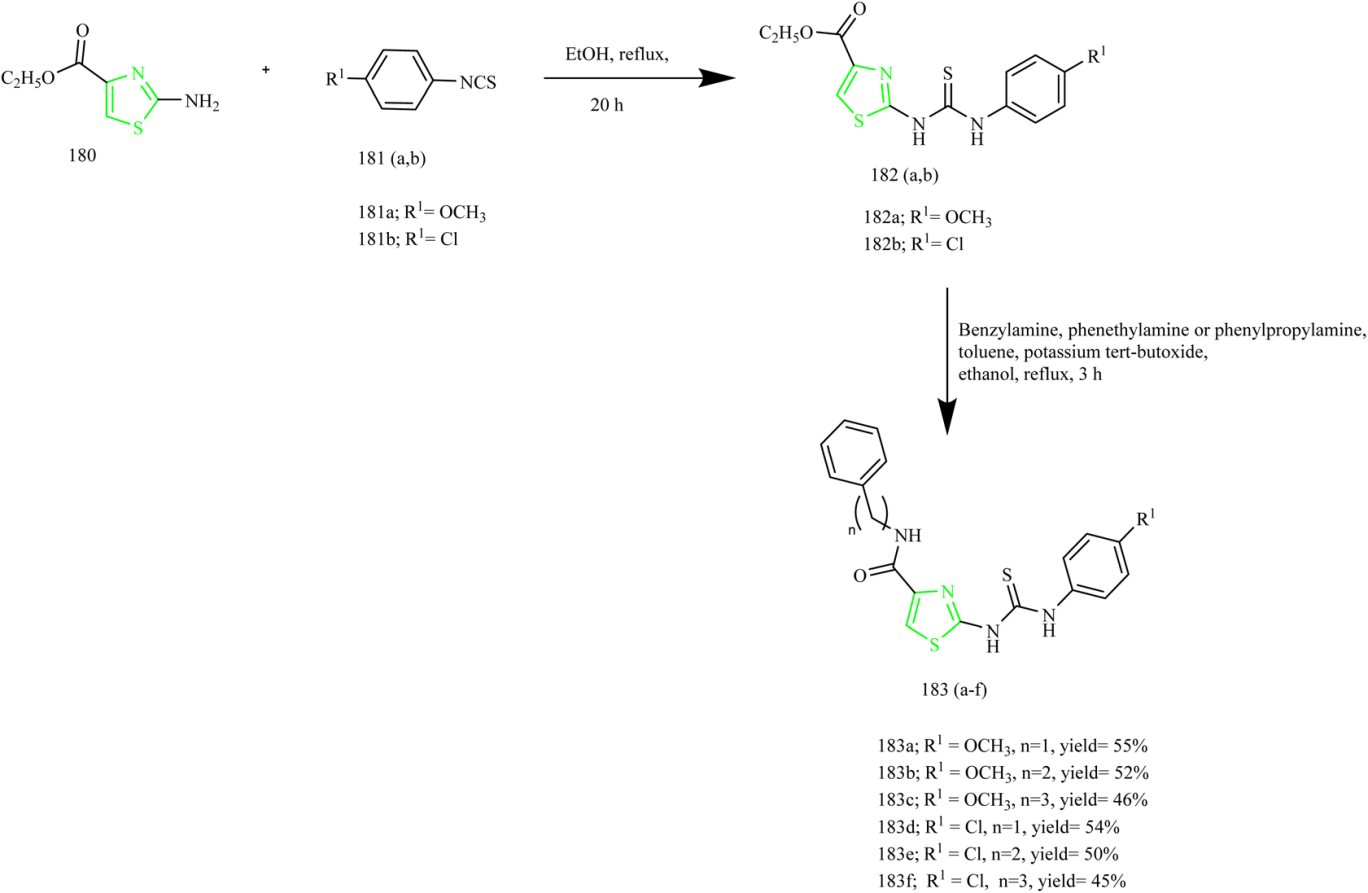
Synthesis of 183(a–f).

**Scheme 39:**
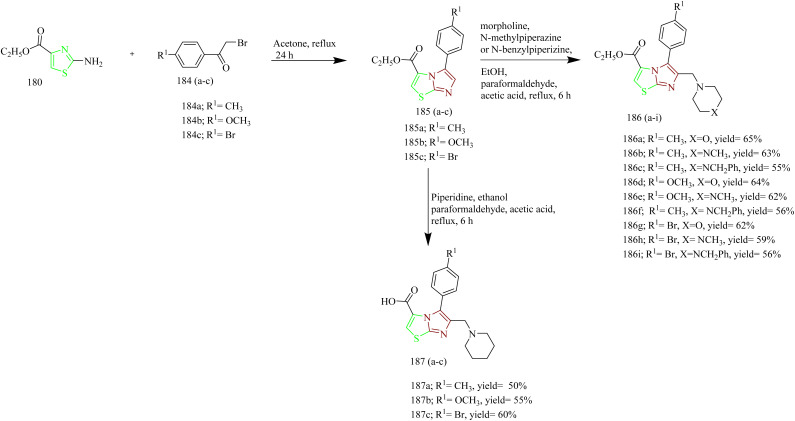
Synthesis of 187(a–c).

**Scheme 40:**
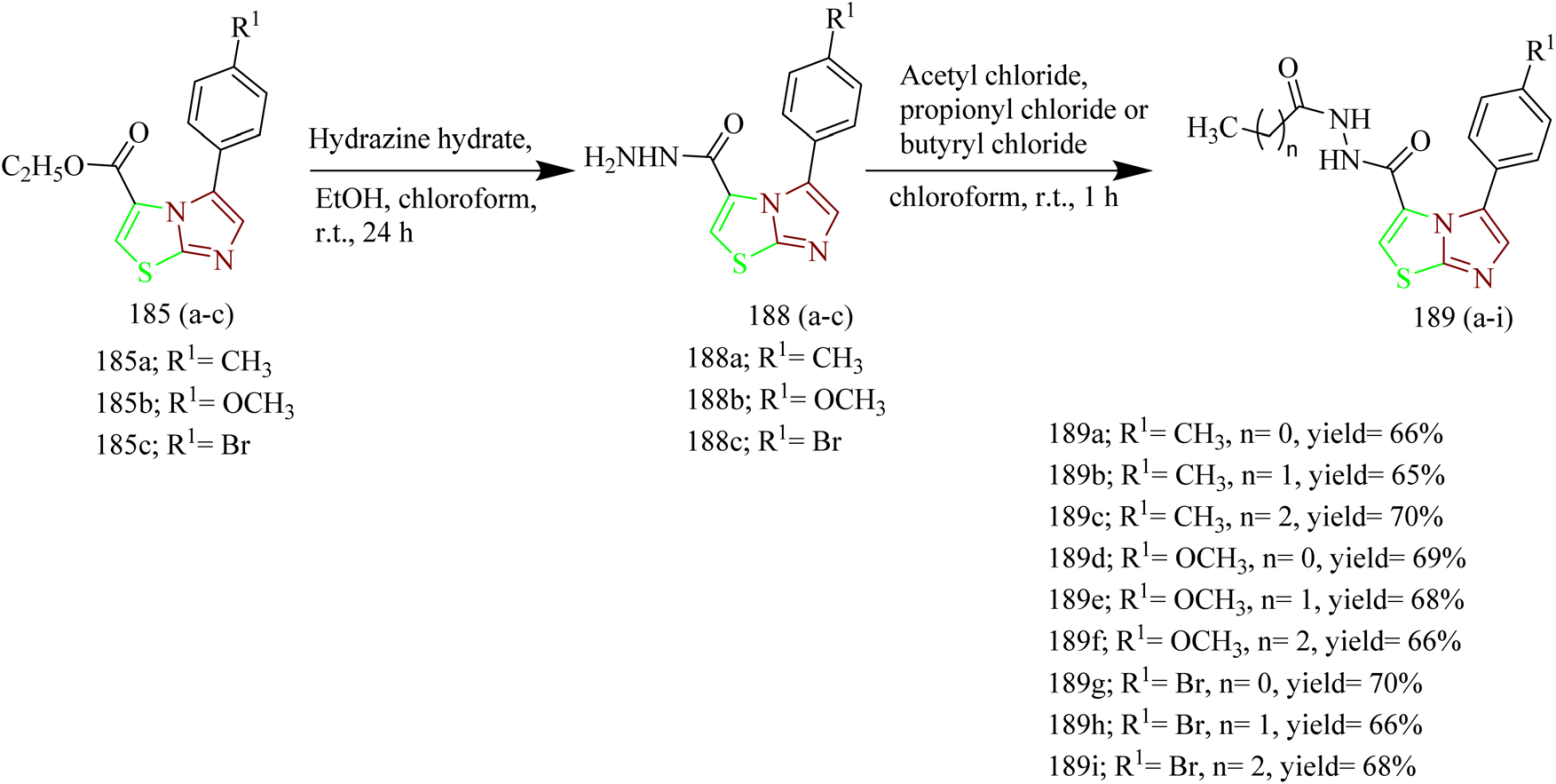
Synthesis of 189(a–i).

**Scheme 41:**
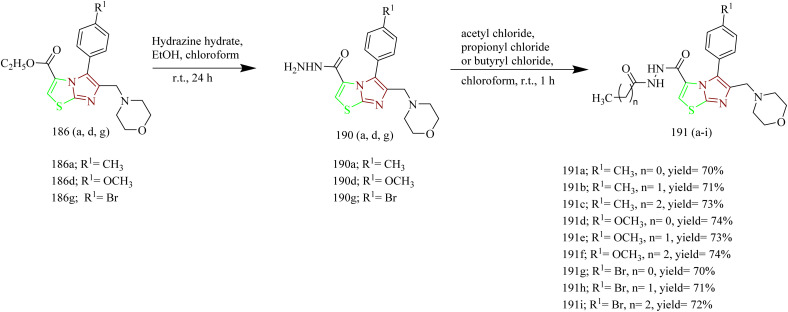
Synthesis of 191(a–i).

Sabry and colleagues synthesized thiazole and imidazole hybrids (Table 3) and evaluated their anticancer activity. The compounds from the series were prepared using synthetic pathways depicted in Schemes 38–41. First, ethyl 2-aminothiazole-4-carboxylate 180 was reacted with substituted phenyl isothiocyanate using ethanol as a solvent under reflux condition to yield intermediates 182(a,b). Then intermediates 182(a,b) were refluxed with suitable primary amine to afford compounds 183(a–f) (Scheme 38). The compounds 185(a–c) were obtained by reacting ethyl 2-aminothiazole-4- carboxylate with various phenacyl bromides 184(a–c) using acetone as a solvent under reflux condition. Next the compounds 185(a–c) were refluxed with heterocyclic secondary amine and paraformaldehyde to yield target compounds 186(a–i). Upon refluxing 185(a–c) with piperidine in presence of paraformaldehyde and acetic acid using ethanol as a solvent yielded imidazo-thiazole carboxylic acid derivative 187(a–c) (Scheme 39). Compounds 185(a–c) were further utilised to get corresponding hydrazide 188(a–c)*via* reaction with hydrazine hydrate. Compounds 185(a–c) were further reacted with acid chlorides to yield their corresponding acylated hydrazides 189(a–i) (Scheme 40). Similarly, the compounds 186a, 186d, and 186g were treated with hydrazine hydrate under same conditions to yield corresponding hydrazides 190a, 190d, and 190g which were further treated with acid chlorides to yield corresponding acylated hydrazides 191(a–i) (Scheme 41). The most potent compounds 187a, 189b, 189g, 190a, 190d, and 190g demonstrated the strongest cytotoxic effects against the MCF-7, with IC_50_ values of 8.29, 5.19, 1.83, 8.46, 2.37, and 6.18 µM, respectively. These results were more effective compared to sorafenib, which showed IC_50_ value of 7.26 µM. In terms of EGFR inhibition, compounds 187a, 189g, and 190d exhibited significant activity compared to sorafenib (IC_50_ 0.051 µM) and gefitinib (IC_50_ 0.062 µM), with IC_50_ values of 0.180, 0.153, and 0.122 µM, respectively. Of the six compounds evaluated, the most effective EGFR kinase inhibitor compounds 189g and 190d exhibited strong binding affinities with binding scores of −8.5 and −9.4 kcal mol^−1^ and demonstrated the most favourable interactions within the EGFR active site. Compounds 189g and 190d, like gefitinib, formed a hydrogen bond with Lys745, in case of compound 189g it was through the nitrogen atom of its imidazo[2,1-*b*]thiazole ring and in compound 190d interaction was *via* its methoxy group. Both compounds also engaged in strong hydrophobic interactions with Ala722 and Phe723. These compounds were observed to interact with Arg841 through arene–cation interactions involving their phenyl substituents, whereas gefitinib engaged Arg841 primarily through strong hydrophobic interactions. Compound 190d, on the other hand, formed an additional hydrogen bond with Asp837 through its terminal hydrazide group, which may account for its enhanced EGFR kinase inhibitory activity.^64^

**Table 3:** Overview of EGFR inhibition activity of thiazole–imidazole hybrids

Compound	Key substituent	Major interactions	EGFR IC_50_ (µM)	SAR observation
179n	3,5-Dimethoxyphenyl substitution	H-bond with Cys773 and Asp831	0.23	Electron donating group improves the better fitting of compound into EGFR pocket.
190d	Methoxyphenyl group and carbohydrazide	H-bond with Lys745 and carbohydrazide moiety forms an additional bond with Asp837	0.122	Electron donating groups were found to increase the conformational preference and planarity
197c	4-bromo substitution	H-bond with Met769 and hydrophobic interaction with Leu820 and Leu768	18.35	Electron withdrawing group like bromide was found to increase the lipophilicity and pocket compatibility
203m	4-Methoxyphenyl group on thiazole ring and 4-chlorophenyl group on the hydrazine-1-carbothiamide	Hydrogen bond with Lys745 and hydrophobic interaction with Leu844, Ala743, Lys797, Leu718, Val726, Leu777, and Leu788	0.087	Electron donating groups facilitated deep pocket penetration while electron withdrawing chloro group boosted hydrophobic binding
210(10)	4-Hydroxyphenyl group	Hydrogen bond with Asp831	9.11	p-hydroxy group increases electron density on the aromatic ring providing better hydrogen bonding capability

**Scheme 42:**
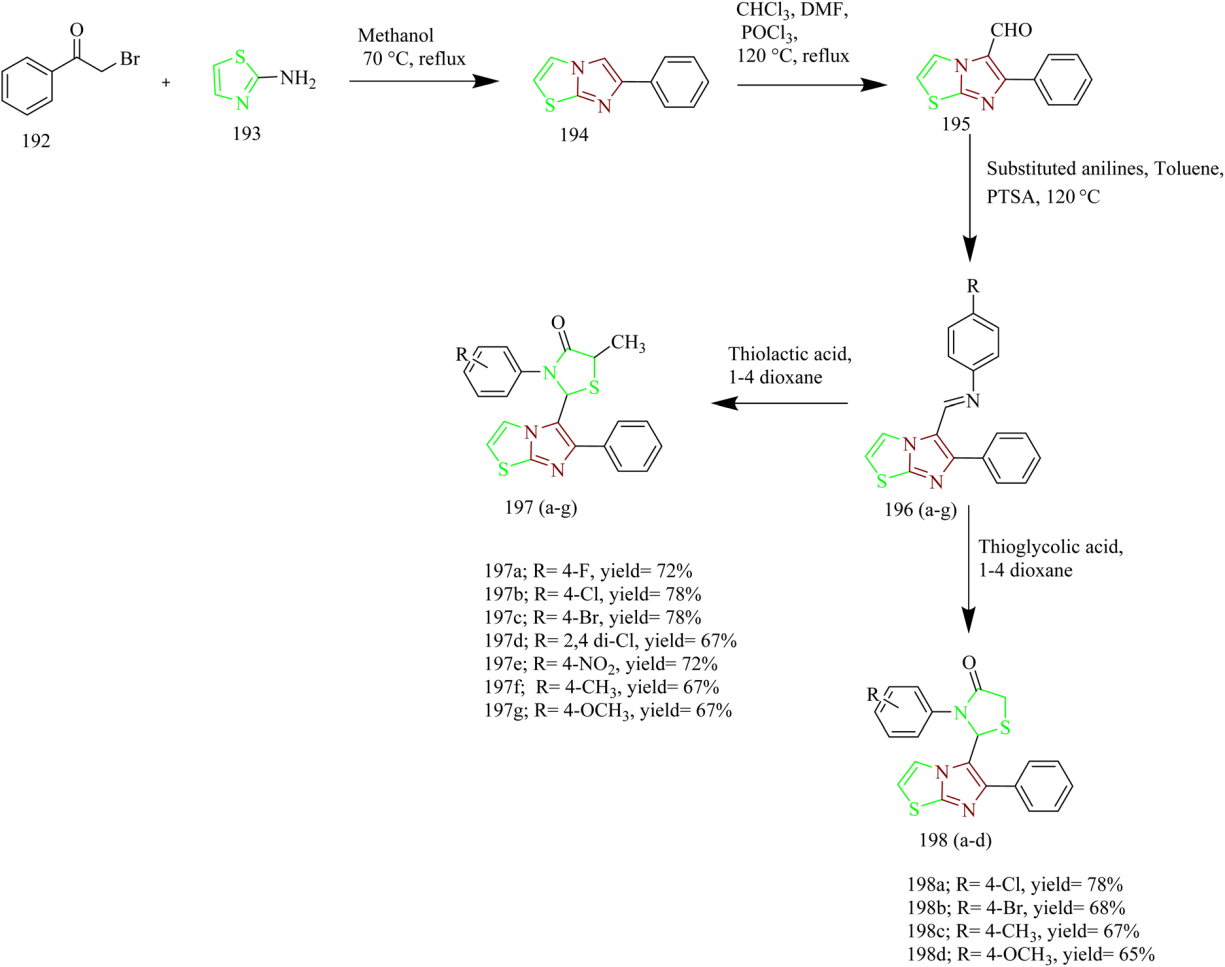
Synthesis of 197 and 198(a–d).

Kamboj *et al.* carried out the synthesis of new imidazothiazole-thiazolidinone compounds as EGFR inhibitors. Imidazothiazole hybrid intermediate 194 was synthesized by reacting phenacyl bromide 192 with aminothiazole 193 using methanol as a solvent under reflux condition. Next the intermediate was subjected to Vilsmeier–Haack reaction in presence of POCl_3_ and chloroform using DMF solvent to yield imidazothiazole carbaldehyde 195. The intermediate 195 was then treated with various anilines in presence of PTSA as a catalyst in toluene solvent to give various Schiff bases 196(a–g). The target compounds 197(a–g) and 198(a–d) were synthesized by the reaction of 196(a–g) with thiolactic acid as well as thioglycolic acid at room temperature in presence of anhydrous zinc chloride as a catalyst in 1–4 dioxane solvent (Scheme 42). The compound 197c displayed IC_50_ value of 10.74 ± 0.40, 18.73 ± 0.88, and 23.22 ± 1.89 µM against the tested cell lines. Further the EGFR inhibitory activity of the compound 197c was studied and it demonstrated potent activity with IC_50_ value of 18.35 µM. SAR analysis revealed that incorporating electron-withdrawing substituents at the para site in phenyl ring of thiazolidinone scaffold led to markedly improved cytotoxicity compared to electron-donating groups such as OCH_3_. Additionally, the compounds in series 197(a–g) showed higher potency compared to series 198(a–d), which lack a methyl group at the 5th position of the thiazolidinone moiety. This enhanced activity is attributed to the ability of methyl group to increase lipophilicity, thereby improving membrane permeability. Molecular docking with EGFR receptor (1M17) demonstrated that compound 197c exhibits strong binding affinity to the active site of the receptor, primarily through a robust hydrogen bond with Met769 which is an essential amino acid residue critical for receptor inhibition.^65^

**Scheme 43:**
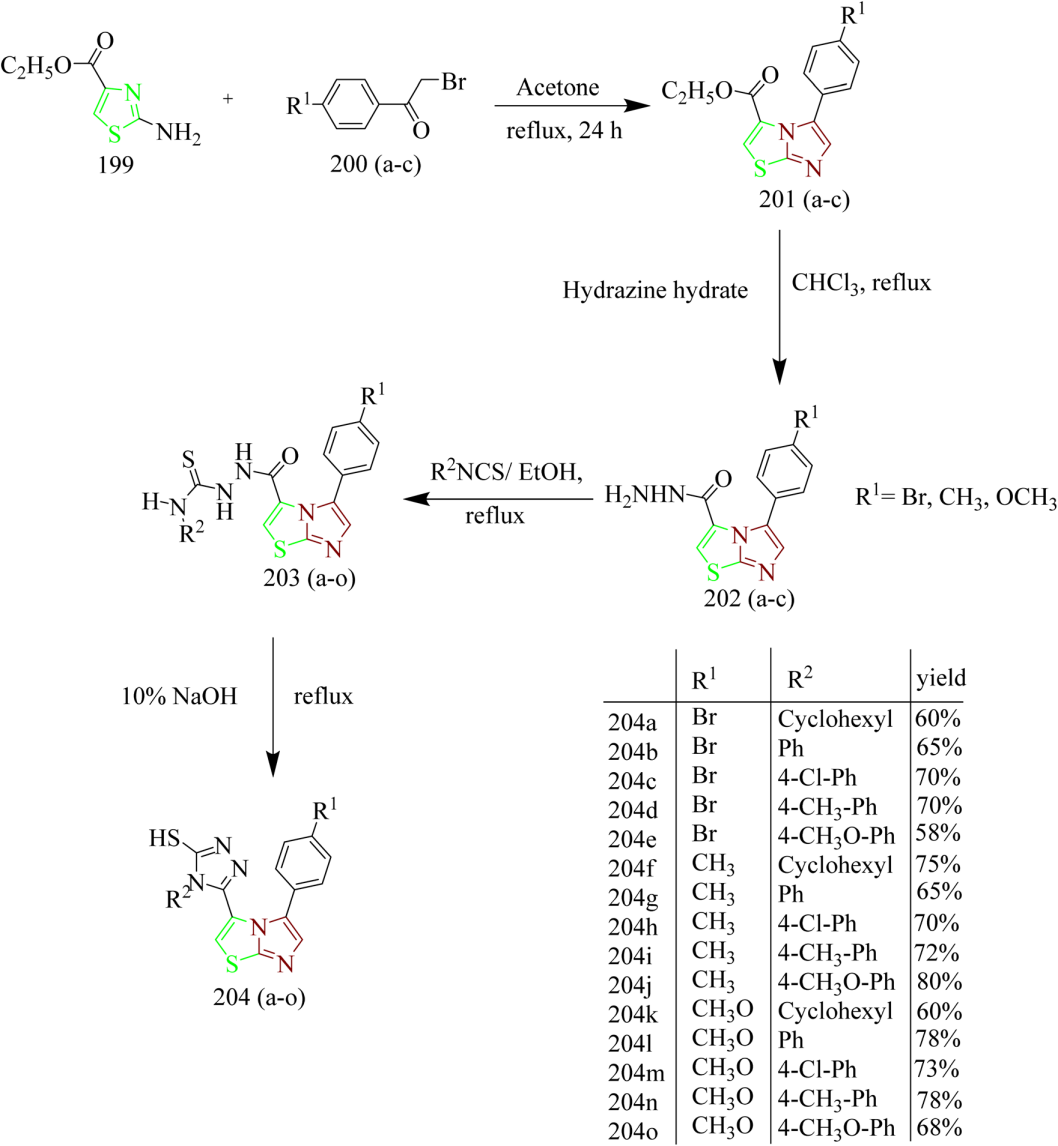
Synthesis of 204(a–o).

Moharram *et al.* designed a series of novel imidazo[2,1-*b*]thiazole analogues targeting EGFR. The hybrid intermediates 201(a–c) were obtained by reacting ethyl 2-aminothiazole-4-carboxylate 199 with various phenacyl bromides 200(a–c) under reflux conditions in acetone. 201(a–c) were then refluxed with hydrazine hydrate in chloroform solvent to yield substituted phenyl-imidazo-thiazole carbohydrazides 202(a–c). The compounds 202(a–c) were then reacted with different phenyl isothiocyanate derivatives in refluxing ethanol to yield corresponding carbothioamides 203(a–o). Further cyclisation of intermediates 203(a–o) using NaOH afforded the final product 204(a–o) (Scheme 43). It was found that compounds 203m and 204n displayed strong activity against MCF-7, with IC_50_ values of 1.81 and 4.95 µM, respectively. These results indicate greater potency compared to the reference drugs doxorubicin and sorafenib, which showed IC_50_ values of 4.17 and 7.26 µM, respectively. Compounds 203m and 204n were recognized as potent inhibitors of EGFR with IC_50_ value of 0.087 and 0.099 µM, respectively when compared to standard sorafenib (IC_50_ = 0.110 µM). With RMSD values of 1.0576 and 1.0664 Å, compounds 203m and 204n were found to bind to the active site of EGFR enzyme. Both the compounds were predicted to form hydrogen bonds with the Lys745. Additionally, they were anticipated to engage in hydrophobic interactions such as π-sigma, π-alkyl, and carbon-hydrogen bonds with several amino acid residues in the active site of enzyme including Leu844, Ala743, Lys797, Val777, and Leu788. Hence compounds 203m and 204n can be regarded as promising new anticancer candidates for further development and optimisation.^66^

**Scheme 44:**
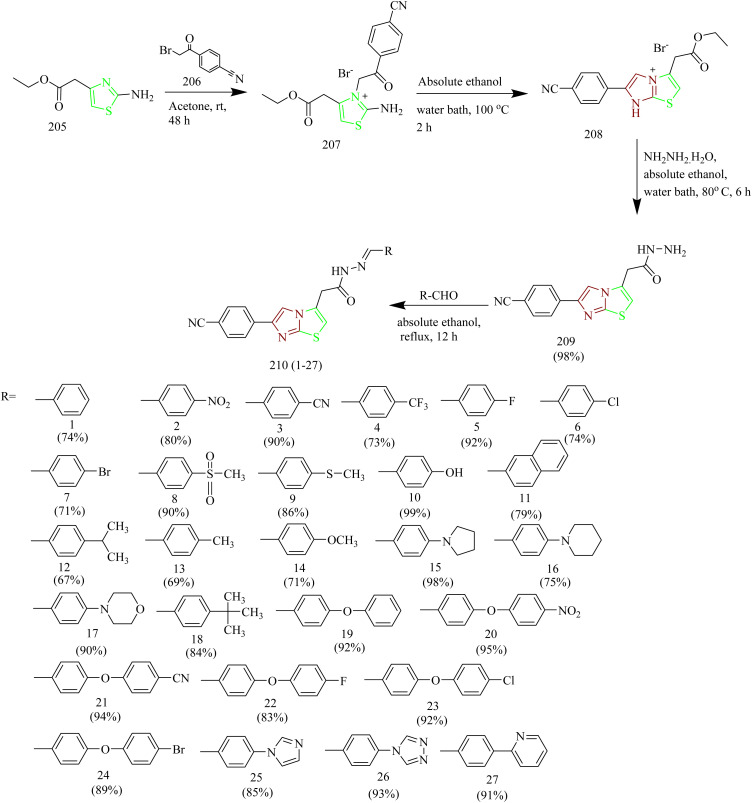
Synthesis of 210(1–27).

Imidazothiazole-hydrazone conjugates as potential EGFR inhibitors for NSCLC therapy was synthesized by Altintop and co-workers. The thiazolium bromide intermediate 207 was obtained by the reaction between ethyl 2-(2-aminothiazol-4-yl)acetate 205 with 2 bromo-4-cyanoacetophenone 206 in acetone solvent. The resulting intermediate 207 was subsequently subjected to cyclisation under reflux conditions in ethanol to yield imidazo-thiazole-acetohydrazide intermediate 208. Compound 208 was treated with hydrazine hydrate leading to the formation of 2-[6-(4-cyanophenyl)imidazo[2,1-*b*]thiazol-3yl]acetohydrazide 209. Finally, compound 209 was treated with various aromatic aldehydes to afford number of N′-arylidene-2-[6-(4-cyanophenyl)imidazo[2,1*b*]thiazol-3-yl]acetohydrazides 210(1–27) (Scheme 44). The compounds 210 (specifically 1, 3, 10, 13, 14, and 18) exhibited significant cytotoxic activity against A549 cell lines with IC_50_ values of 51.28 ± 13.56, 43.67 ± 1.53, 23.75 ± 3.99, 17.70 ± 2.66, 14.35 ± 3.23, and 22.47 ± 4.62 µM. These compounds were further evaluated for their EGFR inhibitory activity in A549 cell lines using *in vitro* colorimetric assays. Compounds 210 (1, 3, 10, 13, 14, and 18) demonstrated EGFR inhibitory activity in A549 cells, exhibiting IC_50_ values of 49.01, 23.13, 9.11, 13.86, 12.89, and 16.50 µM, respectively. When compared to the reference EGFR tyrosine kinase inhibitor erlotinib (IC_50_ = 4.61 µM), compound 210(10) emerged as the most effective inhibitor among the tested series, followed by compounds 210(14 and 13). Molecular docking studies of compounds 210(10,13 and 14) indicate that these molecules are well accommodated within the binding site, where they established a hydrogen bond with an essential part of the Asp-Phe-Gly (DFG) motif.^67^

Across thiazole-imidazole hybrids, the electronic nature of the substituents strongly influences EGFR inhibition. Electron donating groups such as 3,5-dimethoxy and methoxyphenyl (179n and 190d) improved potency by supporting better pocket fit and stronger hydrogen bonding. The 4-bromo analogue 197c gained lipophilicity but showed only moderate activity. In contrast, compound 203m was the most potent, benefiting from deeper pocket penetration from the methoxy group and enhanced hydrophobic contacts from the chloro substituent. The para-hydroxy derivative 210(10) displayed moderate inhibition, aided by increased electron density and hydrogen bonding ability.

### Thiazole–coumarin hybrids

**Scheme 45:**
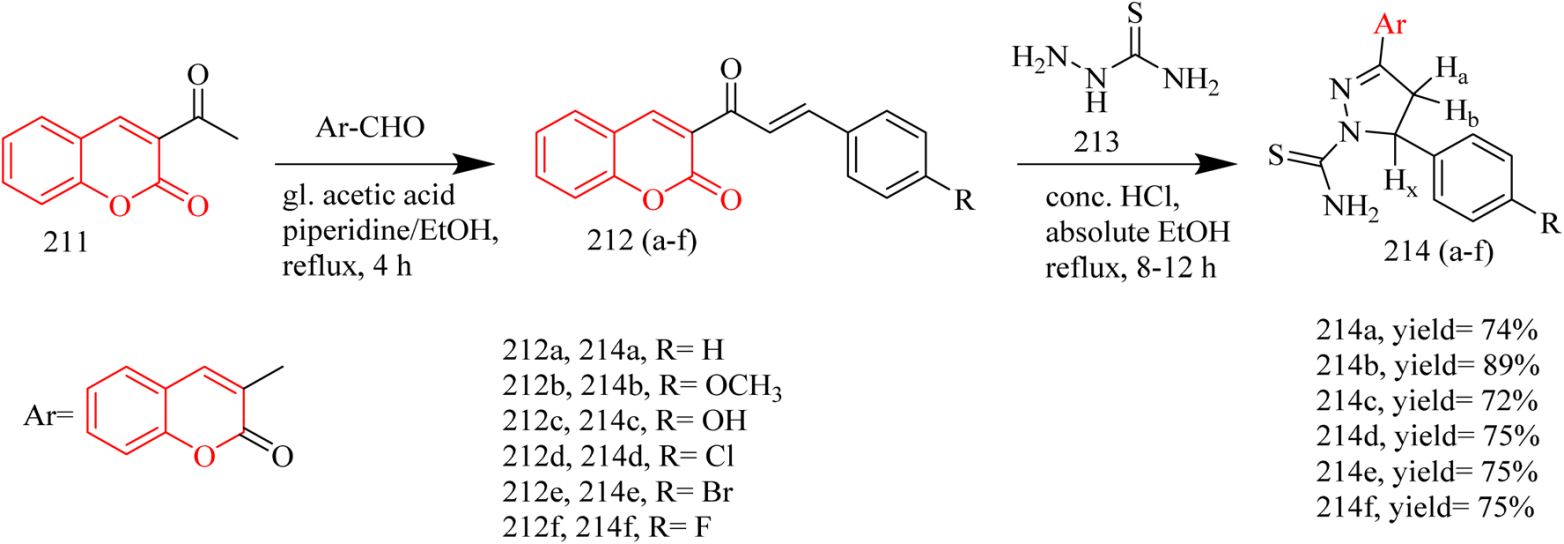
Synthesis of 214(a–f).

**Scheme 46:**
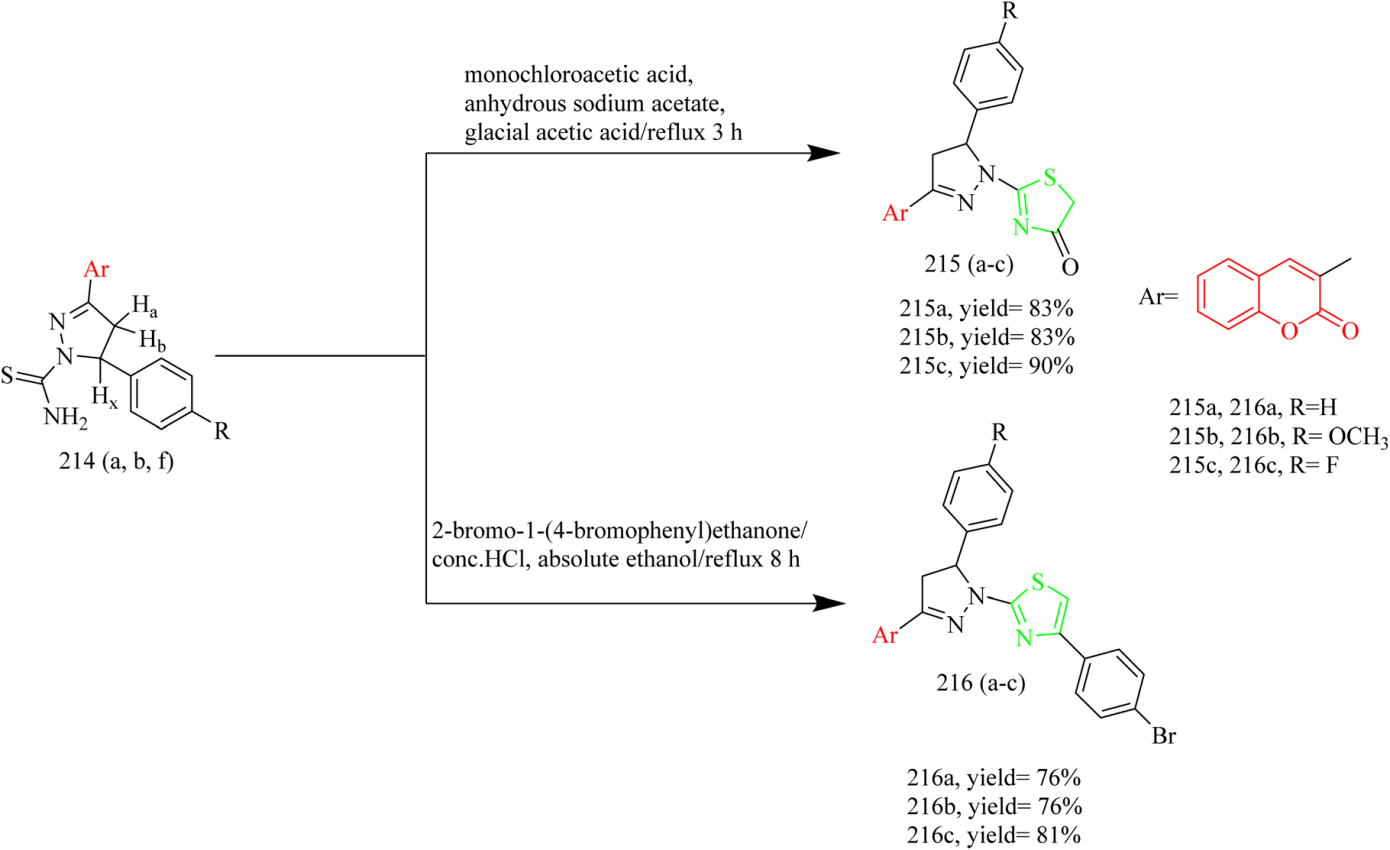
Synthesis of 215(a–c) and 216(a–c).

The synthesis and design of novel coumarin–pyrazoline–thiazole hybrids (Table 4) with cytotoxic potential were carried out by Ragab and coworkers. Precursor 211 was synthesized through a Knoevenagel condensation between salicylaldehyde and ethyl acetoacetate using piperidine as a catalyst. This acetyl precursor 211 was then reacted with a series of aromatic aldehydes under Claisen–Schmidt conditions using piperidine and acetic acid to afford chalcones 212(a–f). The reaction of thiosemicarbazide 213 with chalcone intermediates 212(a–f) under acidic conditions afforded novel coumarin–pyrazoline hybrids bearing a thiourea moiety, designated as compounds 214(a–f) (Scheme 45). Refluxing thioamide derivatives 214(a,b and f) with monochloroacetic acid and sodium acetate in glacial acetic acid, or with 2-bromo-1-(4-bromophenyl)ethenone in ethanol containing HCl, led to the formation of thiazolone derivatives 215(a–c) and thiazole derivatives 216(a–c), respectively (Scheme 46). Compound 214d, which contains a *p*-chlorophenyl group, demonstrated an IC_50_ value of 5 nM against MCF-7. In comparison to compound 216a, featuring a phenyl thiazole moiety, demonstrated an IC_50_ of 5 nM against HCT-116 colon cancer cell lines and 20 nM against MCF-7 cells. Notably, both compounds showed good selectivity, indicating significantly higher IC_50_ values of 35.78 µM for 214d and 22.77 µM for 216a against the normal breast cell line MCF-10A. Compounds 214d, 214e, 214f, 216a, and 216c demonstrated strong EGFR inhibitory activity, ranging from 80.9% to 88%, in comparison to erlotinib, which showed 95.68% inhibition. Among them, compound 214d exhibited highest EGFR inhibition, achieving 88.0% inhibition. Docking studies revealed that compound 214d established two key interactions within the EGFR binding site which is a hydrogen bond with the side chains of Asp831 and Asn818, and an aromatic–aromatic interaction with the adjacent Phe989 residue.^68^

**Table 4:** Overview of EGFR inhibition activity of thiazole–coumarin hybrids

Compound	Key substituent	Major interactions	EGFR IC_50_ (µM)	SAR observation
214d	Chlorophenyl substitution	Hydrogen bond with Asp831 and Asn818, and aromatic interaction with Phe989	—	Chlorine atom enhanced the molecule's lipophilic character allowing it to fit more efficiently within the EGFR active site
227b	7-Isobutoxy group	Hydrogen bond with Met793 and hydrophobic interactions with Ala743, Lys745, Leu844, Asn842, Arg841, Phe723, Val726, Lys728, Leu718, and Val717	0.15	The bulky 7-isobutoxy group enhances hydrophobicity, allowing the molecule to anchor more effectively within the hydrophobic pocket of EGFR
234a	4-Methylthiazole-5-carboxylate	Hydrogen bond with Met769 Leu694, Lys721, Thr830, and Asp831	0.184	The 4-methylthiazole-5-carboxylate group enhances activity by providing good hydrogen-bonding potential with improved hydrophobic complementarity resulting in more stable binding within the EGFR pocket

**Scheme 47:**
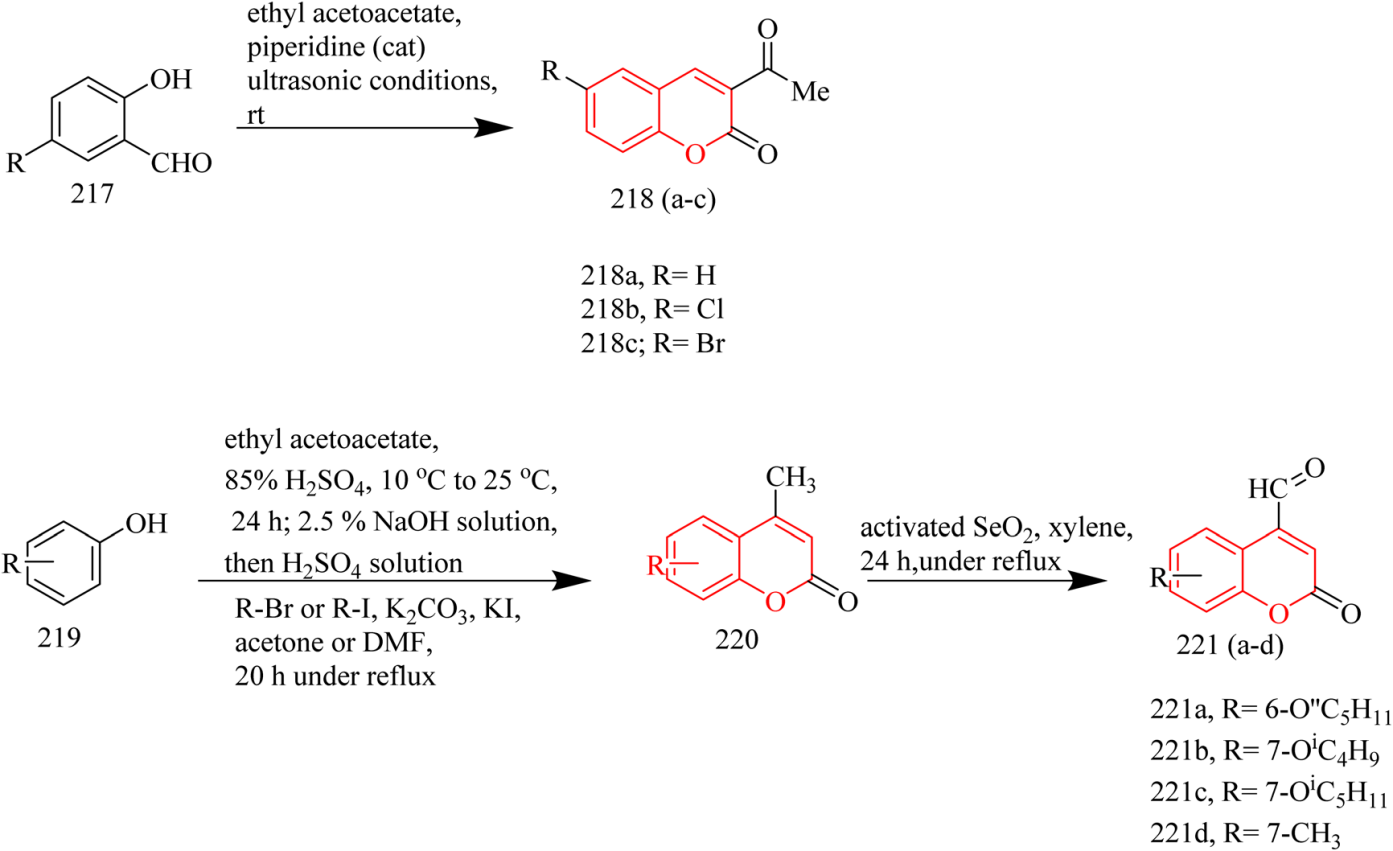
Synthesis of 221(a–d).

**Scheme 48:**
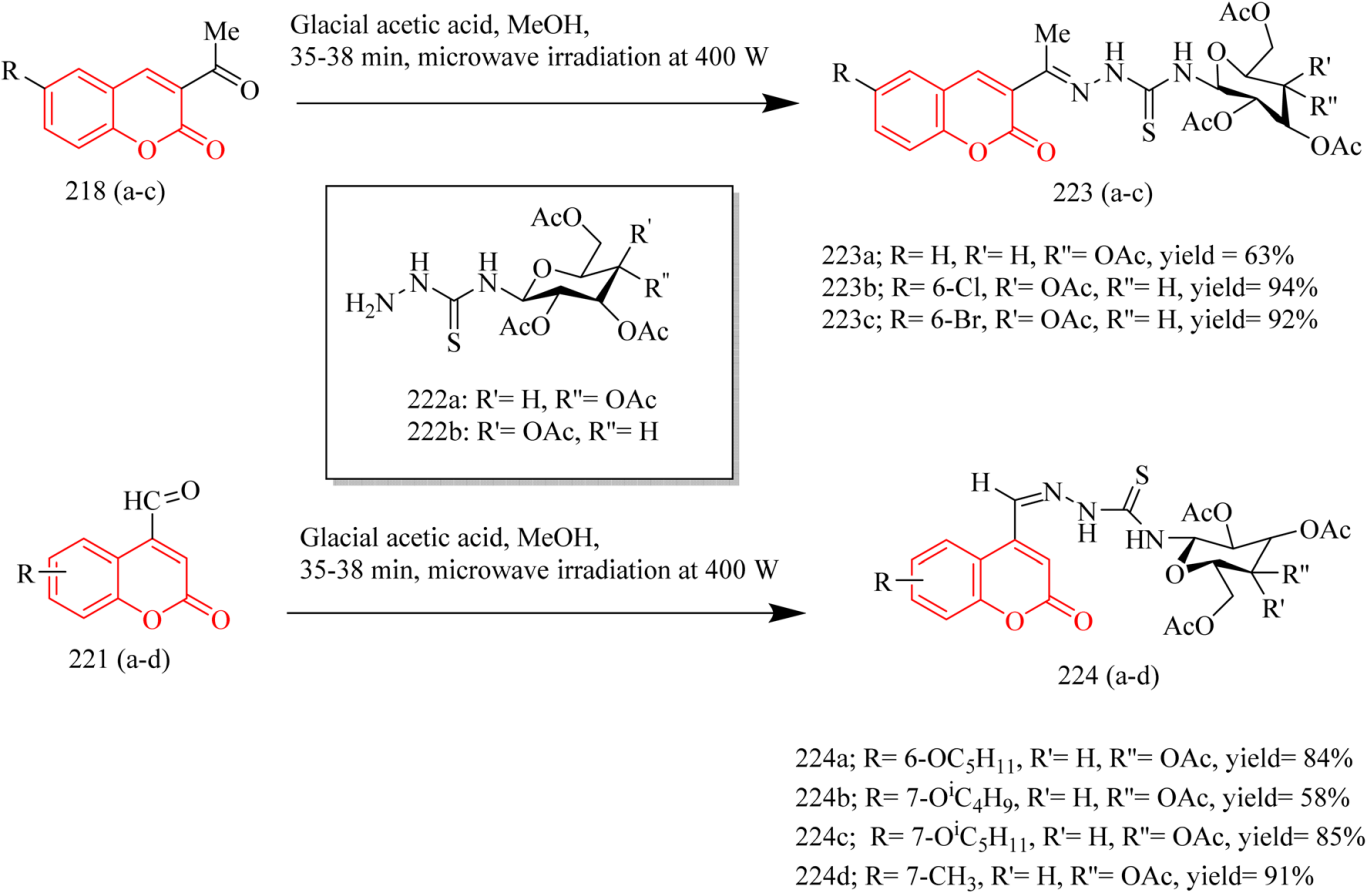
Synthesis of 224(a–d).

**Scheme 49:**
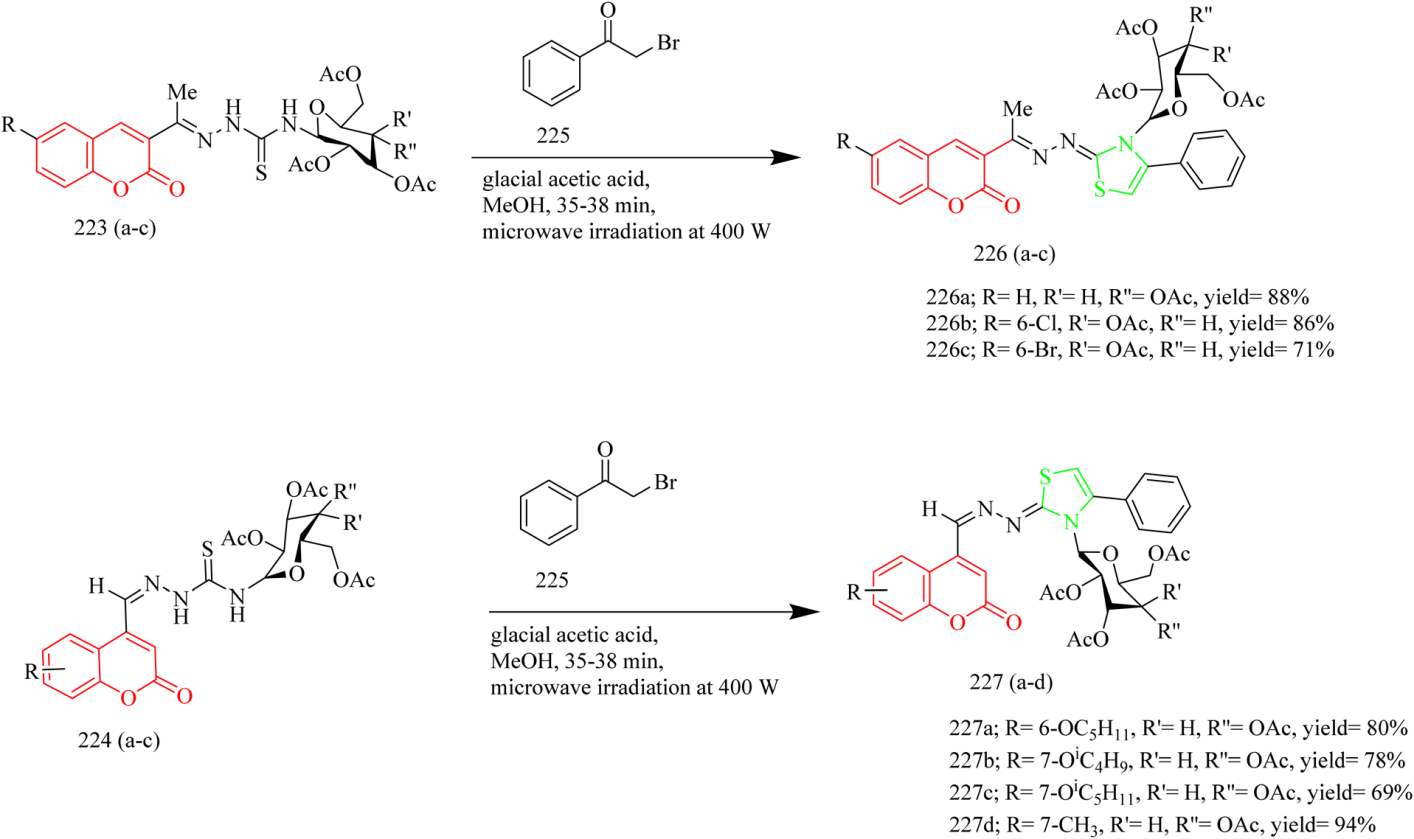
Synthesis of 226(a–d) and 227(a–d).

In a recent investigation, Ngoc Toan and coworkers developed a range of thiazoline–coumarin hybrid molecules linked with sugar units, with the aim to improve their solubility and therapeutic potential. The synthesis involved the condensation of appropriate unsubstituted salicylaldehyde 217 with ethyl acetoacetate resulting in the formation of unsubstituted acetylcoumarins 218(a–c). In a similar manner, additional compounds were synthesized starting from substituted phenols 219, which reacted with ethyl acetoacetate using 85% sulfuric acid as a catalyst to produce coumarin derivatives. These derivatives were then converted into the corresponding alkoxy-4-methylcoumarins 220. Finally, oxidation of the 4-methyl group using selenium dioxide afforded the desired formylcoumarins 221(a–d) (Scheme 47). Then the appropriate thiosemicarbazide derivatives 222a and 222b, derived from d-glucose and d-galactose respectively, were reacted with 3-acetyl- and 4-formyl-coumarins 218(a–c) and 221(a–d). The reaction was carried out under microwave-assisted heating at 400 W, and this afforded thiosemicarbazone compounds 223(a–c) and 224(a–d) (Scheme 48). Subsequently, the previously synthesized thiosemicarbazones 223(a–c) and 224(a–d) were transformed into 2,3-dihydro-2(3H)-thiazole derivatives 226(a–c) and 227(a–d) through a reaction with ω-bromoacetophenone in the presence of anhydrous sodium acetate, which acted as a base (Scheme 49). The compound 227a exhibited strongest activity against HepG2, HeLa, SK-MeI-2, Lu-1 with IC_50_ values of 1.35–3.12 µM. Whereas the compound 227b showed good activity against MCF-7 cell lines with IC_50_ value of 2.98 µM. The compounds 226b, 226c, 227a, 227b, and 227c showed notable EGFR inhibition. Among these the compound 227b was found to be most potent compound as an EGFR inhibitor with IC_50_ value of 0.15 µM, when compared to Sorafenib which had IC_50_ value of 0.11 µM. It was found that the position of substituent on the coumarin ring significantly influenced the activity. In case of 227b presence of branched alkoxy chains at position 7 enhanced the EGFR inhibitory activity. Also, this compound was found to form effective hydrogen bonding with Met793 residue of the EGFR enzyme with a good BE of −6.63 kcal mol^−1^. Hence the compound 227b was marked as the best anticancer agent in the series, showing balanced activity across multiple cancer cell lines and strong kinase inhibition.^69^

**Scheme 50:**
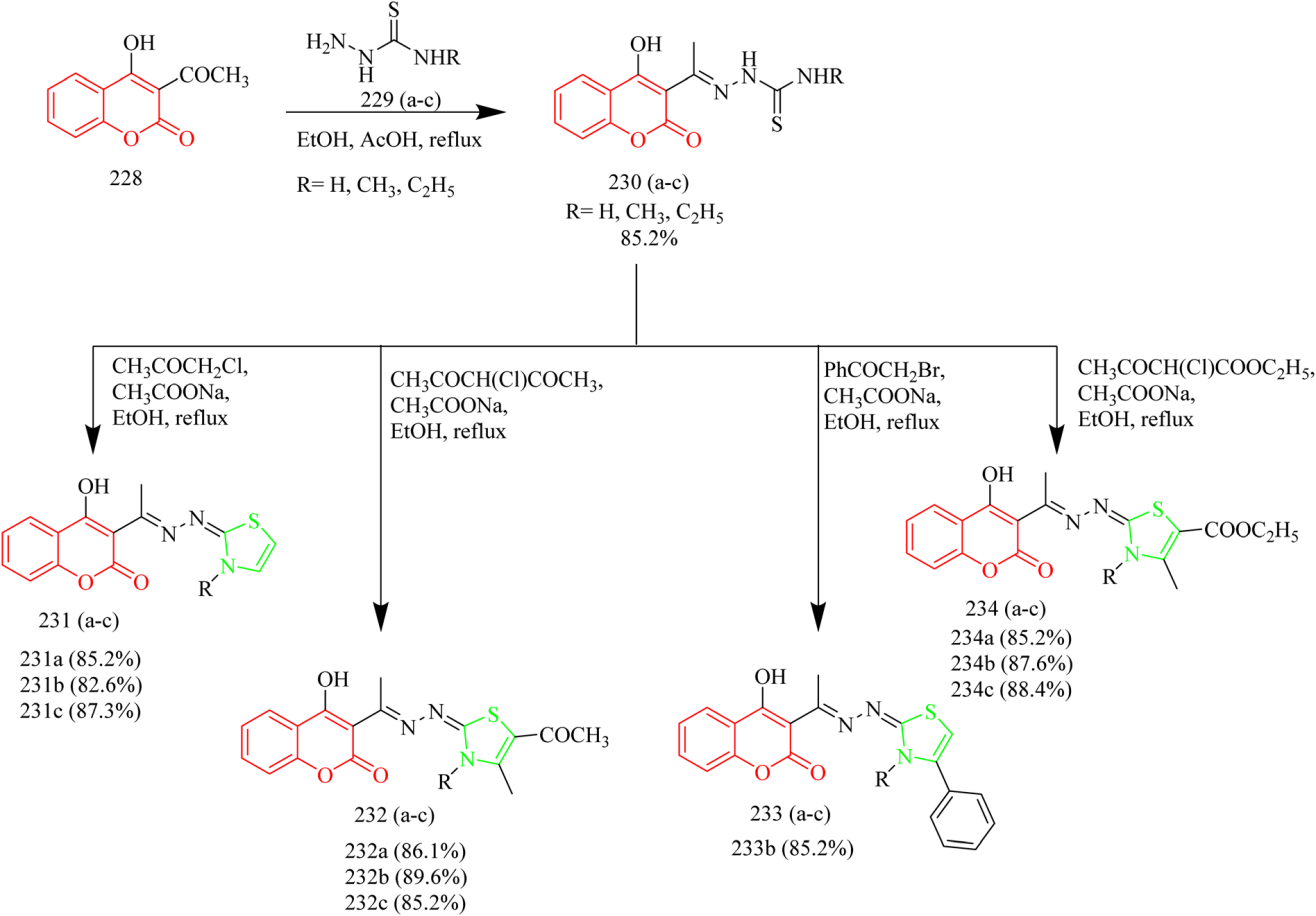
Synthesis of 231(a–c), 232(a–c), 233(a–c), and 234(a–c).

**Scheme 51:**
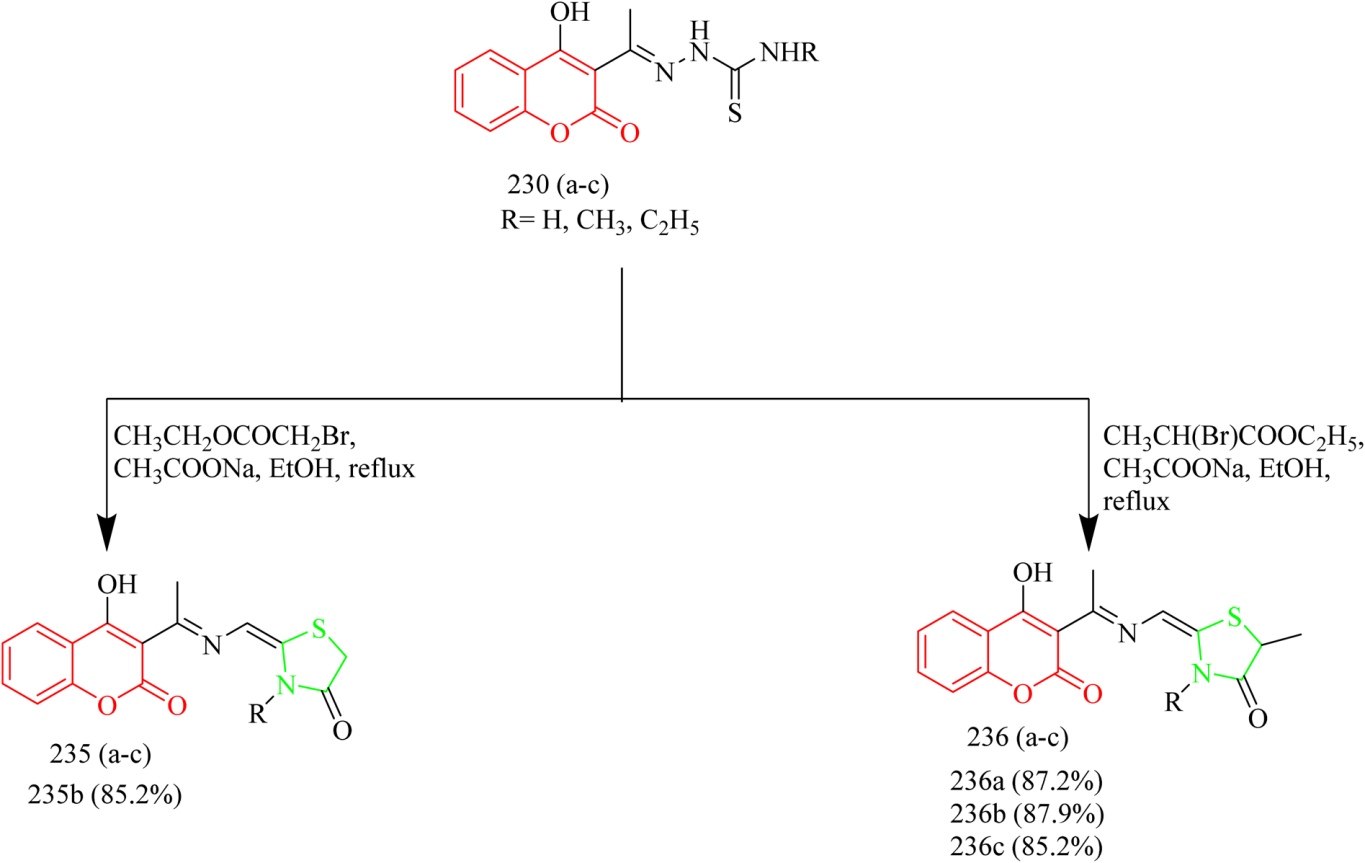
Synthesis of 235(a–c) and 236(a–c).

**Scheme 52:**
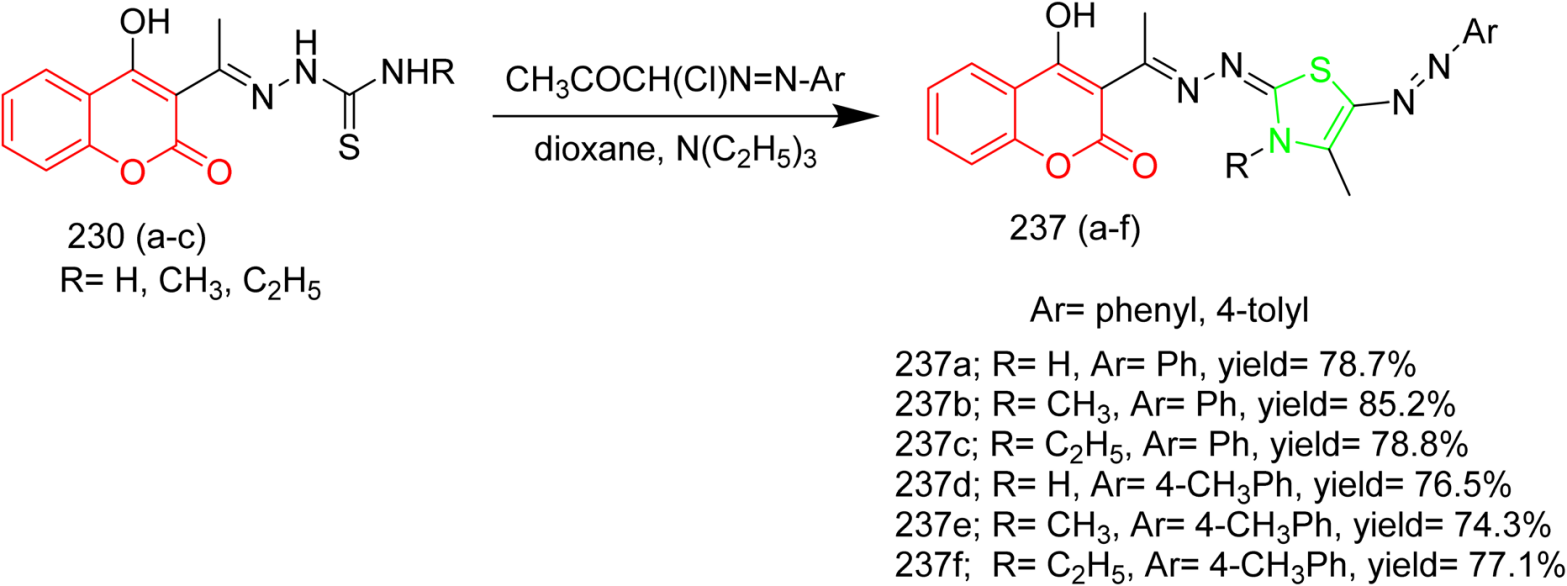
Synthesis of 237(a–f).

Batran *et al.* synthesized thiazole–coumarin hybrid compounds, aiming to evaluate their potential as anticancer agents and inhibitors of EGFR. The synthesis involved the reaction of chromen-2-one precursor 228 with various thiosemicarbazides 229(a–c), resulting in the formation of the corresponding thiosemicarbazone derivatives 230(a–c). Thiosemicarbazones 230(a–c) were treated with various α-haloketones in ethanol, to afford the corresponding methylthiazolines 231(a–c) and acetyl-4-methylthiazolines 232(a–c), respectively (Scheme 50). Similarly, the reaction of 230(a–c) with phenacyl bromide yielded the corresponding 4-phenylthiazoline derivatives 233(a–c), while treatment with ethyl-2-chloroacetoacetate afforded the 4-methylthiazole-5-carboxylates 234(a–c) (Scheme 50). Additionally, cyclization of thiosemicarbazones 230(a–c) with ethyl bromoacetate and ethyl 2-bromopropionate under identical conditions furnished thiazolidinones 235(a–c) and 5-methylthiazolidinones 236(a–c), respectively (Scheme 51). Furthermore, the desired compounds 237(a–f) were synthesized by reacting thiosemicarbazones 230(a–c) with hydrazonoyl chlorides (Scheme 52). 4-Methylthiazole-5-carboxylate derivative (Compound 234a) was identified as the most potent compound of the series with good inhibition activity against MCF-7 (IC_50_ = 6.4 µM, HCT-116 (IC_50_ = 20.4 µM), and HepG2 (IC_50_ = 57.2 µM) cell lines. Furthermore, this compound exhibited impressive inhibition against EGFR enzyme with IC_50_ value of 0.184 µM which is close to the IC_50_ value of erlotinib (IC_50_ = 0.088 µM). From SAR analysis it was found that the presence of ester group on thiazole ring increased the activity through hydrogen bond forming ability. Also, the carboxylate group played a crucial role in forming hydrogen bonds with important residues like Met769, Leu694, and Lys721 of the EGFR enzyme with the docking score of −9.265 kcal mol^−1^. The thiazole–coumarin hybrid scaffold offers both planar π-interactions and polar contacts, stabilising the binding in kinase active sites.^70^

In the thiazole–coumarin hybrid series, compounds 214d, 227b, and 234a all showed strong EGFR binding through a balanced mix of hydrogen-bonding and hydrophobic interactions. 214d benefited from its chlorophenyl group, where the chlorine improved lipophilicity and helped the molecule fit more comfortably within the active site. 227b, containing a 7-isobutoxy group, displayed the highest potency (IC_50_ = 0.15 µM) due to key hydrogen bond and extensive hydrophobic contacts that allowed it to anchor firmly in the pocket. Meanwhile, 234a, with a 4-methylthiazole-5-carboxylate substituent (IC_50_ = 0.184 µM), formed several hinge-region hydrogen bonds and complemented the surrounding hydrophobic environment, giving it a stable and well-oriented binding pose.

### Thiazole–triazole hybrids

**Scheme 53:**
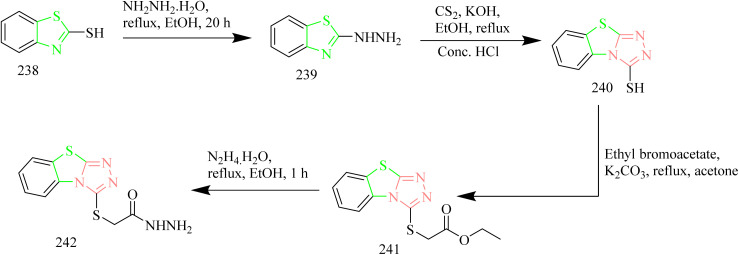
Synthesis of 242.

**Scheme 54:**
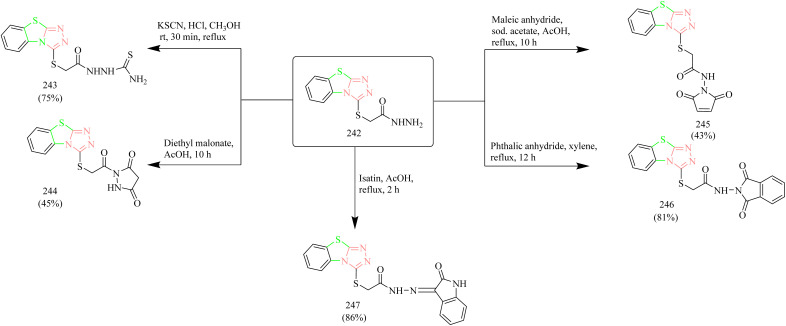
Synthesis of 243, 245, and 246.

Novel benzothiazolo–triazolo hybrids (Table 5) as anticancer and EGFR inhibitor was designed and synthesized by Abdelazeem and coworkers. Synthesis involved the reaction of thiol precursor 238 with hydrazine hydrate, resulted in the formation of 2-hydrazinylbenzo[*d*]thiazole 239. This intermediate was then cyclized using carbon disulfide under acidic conditions in ethanol, yielding thiazole-triazole intermediate 240. Subsequent reaction of compound 240 with ethyl bromoacetate under basic conditions in acetone, followed by treatment with hydrazine hydrate, produced two products ethyl 2-(benzo[4,5]thiazolo[2,3-c][1,2,4]triazol-3-ylthio)acetate 241 and 2-(benzo[4,5]thiazolo[2,3-c][1,2,4]triazol-3-ylthio)acetohydrazide 242 (Scheme 53). Finally, the acid hydrazine derivative 242 was subjected to cyclisation reactions with various reagents including diethyl malonate, maleic anhydride, isatin, and phthalic anhydride resulting in the formation of number of heterocyclic compounds labelled from 243–247 (Scheme 54). It was found that the compound 247 with isatin moiety showed the anticancer activity comparable to standard doxorubicin with IC_50_ values of 3.53, 2.90, and 2.40 µM against A549, MCF-7, and Hep3B cell lines. The docking study revealed that compound 247 exhibited the most favourable binding affinity toward EGFR, with a BE of −9.58 kcal mol^−1^ which is significantly lower than that of reference ligand erlotinib (BE −7.39 kcal mol^−1^).^71^

**Table 5:** Overview of EGFR inhibition activity of thiazole–triazole hybrids

Compound	Key substituent	Major interactions	EGFR IC_50_ (µM)	SAR observation
247	Isatin moiety	H bonding with Met769, Thr766	—	The bulky, electron rich isatin ring helps the molecule to interact more effectively with its target and boosts its activity inside cells
253c	Hydroxylated glycosides	H bonding with Met769, Thr766, Gly767, Asp831	0.12	The presence of free hydroxyl groups improves solubility and allows the molecule to adopt a better orientation
258m	3,5-Dicyano groups on aryl rings	H bonding and π-stacking interaction with Lys851 and Trp856	0.18	The 3,5-di-cyano groups on the aryl ring enhance binding affinity and cytotoxicity, likely due to increased polarity and hydrogen bonding potential
266d	*p*-nitro substituent	H bonding with Lys721, Gly700, Phe699, and Ala698	0.210	The p-nitro substituent enhances polarity and hydrogen bonding capacity, improving interaction with EGFR active site

**Scheme 55:**
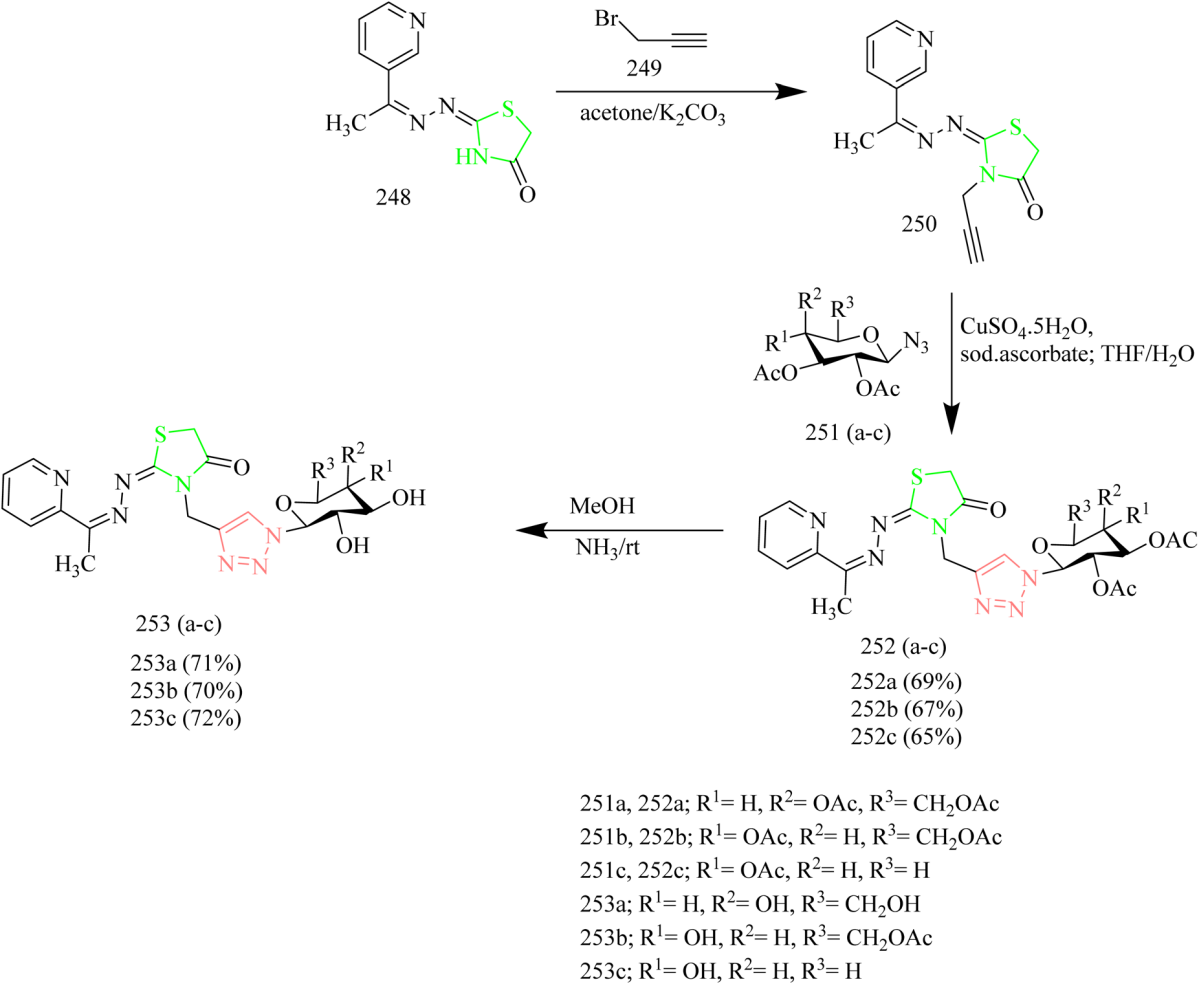
Synthesis of 253(a–c).

Kassem *et al.* designed and synthesized a new series of pyridine–thiazolidinone–triazole hybrid glycosides aimed at targeting EGFR enzyme. The synthesis utilized a click chemistry approach, beginning with the preparation of a terminal acetylenic intermediate through the alkylation of key compound 248 with propargyl bromide 249. The resulting acetylenic derivative 250 was then subjected to Cu(i)-catalyzed click reaction with various acetylated glycopyranosyl azides 251(a–c), leading to the formation of the desired thiazole-triazole hybrid compounds 252(a–c) (Scheme 55). Compound 253c, a hydroxylated sugar-containing pyridine-thiazolidinone-triazole glycoside, demonstrated the strongest cytotoxic effect against HepG-2 and MCF-7 cancer cell lines, with IC_50_ values of 2.09 µM and 0.51 µM, respectively. It also exhibited minimal toxicity towards normal cells. Additionally, 253c showed potent EGFR inhibitory activity, with an IC_50_ value of 0.12 µM compared to the standard drug erlotinib, which has an IC_50_ value of 0.15 µM. SAR analysis revealed that hydroxylated glycosides exhibited enhanced cytotoxic activity compared to their acylated counterparts which suggest that the hydroxyl group facilitates stronger hydrogen bonding while triazole unit contributes to improved binding affinity through electron donating effects. Docking analysis revealed that compound 253c occupied the same binding site on EGFR as the reference inhibitor erlotinib. It established hydrogen bonds with key amino acid residues Met769, Gly772, and Asp831 within the EGFR active site.^72^

**Scheme 56:**
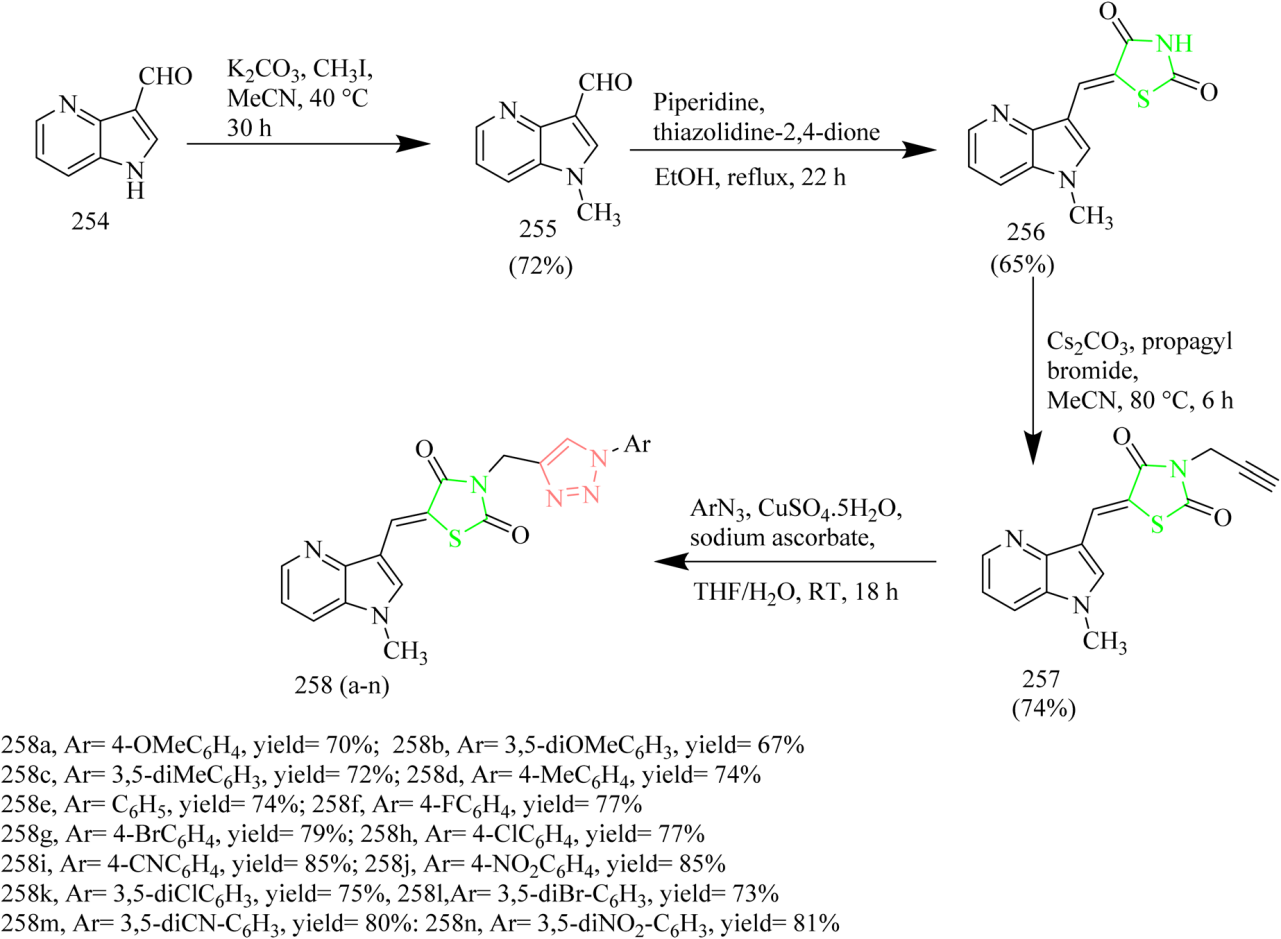
Synthesis of 258(a–n).

Shreedhar Reddy *et al.* carried out the synthesis of 4-azaindole-thiazolidine-2,4-dione based compounds intergrated with 1,2,3-triazoles, designed to target EGFR for anticancer applications. The target molecules 258(a–n) were synthesized *via* multi-step process. Initially, 4-azaindole aldehyde 254 underwent methylation with methyl iodide in a basic medium to produce an intermediate 255. This was followed by condensation with thiazolidine-2,4-dione, generating compound 256. Subsequent alkylation with propargyl bromide afforded the terminal alkyne intermediate 257. In the final step, a Cu(i)-catalyzed azide alkyne cycloaddition (click reaction) with various aromatic azides yielded the desired 1,2,3-triazole containing derivatives 258(a–n) (Scheme 56). Compound 258m exhibited outstanding cytotoxic effects against three cancer cell lines MCF-7, A549, and HepG2 with IC_50_ values of 1.71, 7.15, and 5.15 5.15 µM, respectively. Additionally, compound 258m displayed potent EGFR inhibitory activity, with an IC_50_ value of 0.18 µM making it approximately twice as effective as erlotinib, which possess an IC_50_ value of 0.41 µM. Molecular docking analysis confirmed that compound 258m fits precisely into the active site of EGFR, exhibiting a favorable BE of −10.37 kcal mol^−1^. It forms key interactions with critical residues Lys851 and Trp856, supporting its high binding affinity and potent EGFR inhibitory activity. Hence the compound 258m demonstrated dual functionality by integrating both anticancer activity and kinase inhibition within a single molecular structure.^73^

**Scheme 57:**
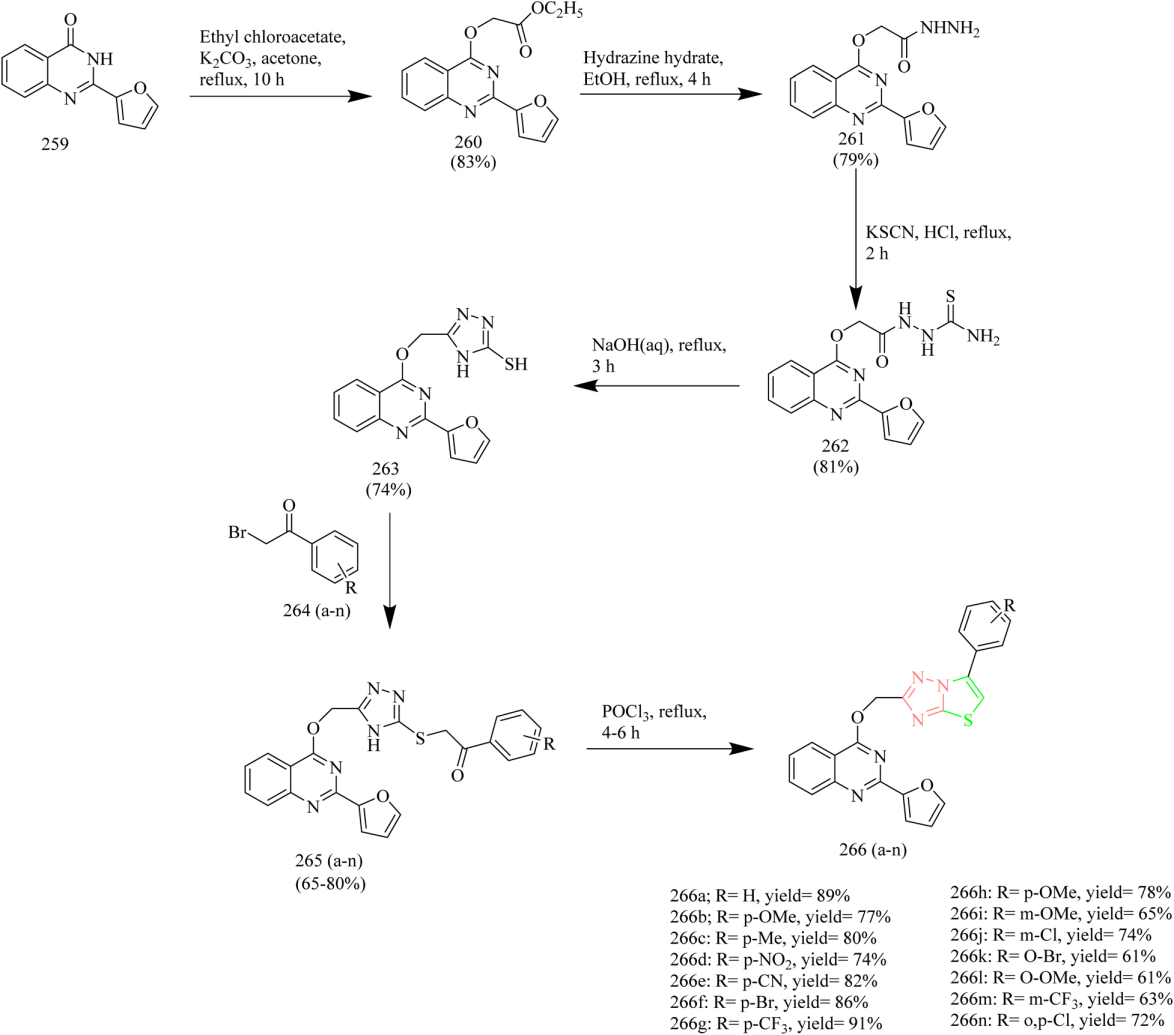
Synthesis of 266(a–n).

Farveen *et al.* reported the synthesis of novel quinazoline–thiazolotriazole hybrid molecules as promising EGFR inhibitors through anticancer activity assessment and computational evaluations. The synthetic route commenced with the reaction of 2-(furan-2-yl)quinazolin-4(3H)-one 259 and ethyl chloroacetate under basic conditions to afford ethyl 2-((2-furan-2-yl)quinazolin-4-yl)oxy)acetate 260. This ester was subsequently treated with hydrazine hydrate to produce the corresponding hydrazide 261. Further reaction with potassium thiocyanate in an acidic medium yielded the key thioamide intermediate 262, which underwent cyclization in the presence of aqueous sodium hydroxide to form 5-(((2-furan-2-yl)quinazolin-4-yl)oxy)methyl)-4h-1,2,4-triazole-3-thiol 263. Intermediate 263 was then condensed with various phenacyl bromides 264(a–n) under ethanol reflux yielded intermediates 265(a–n). Finally, treatment of these intermediates with POCl_3_ under reflux conditions furnished the target thiazolo–triazole hybrid compounds 266(a–n) (Scheme 57). From anticancer evaluation it was found that the compound 266d exhibited superior cytotoxicity against MCF-7 and A549 cancer cell lines with IC_50_ values of 3.70 ± 0.55 µM and 8.45 ± 0.16 µM, respectively, outperforming standard drug erlotinib which had an IC_50_ value of 4.61 ± 0.12 µM and 8.93 ± 0.63 µM. Compound 266d also demonstrated potent EGFR inhibition, with an IC_50_ value of 0.210 ± 0.01 µM, significantly surpassing erlotinib, which showed an IC_50_ value of 0.421 ± 0.003 µM. From SAR analysis it was found that the electron withdrawing groups at the para-position, such as –NO_2_ in 266d and –CN in 266e, markedly improve activity by strengthening hydrogen bonding and π-interactions within the EGFR binding site. Docking analysis revealed that compound 266d established six hydrogen bonds with key EGFR residues, including Lys721, Gly700, Phe699, and Ala698, which likely contributes to its strong binding affinity. Despite exhibiting potent activity comparable to erlotinib, compound 266d showed moderate cytotoxicity toward normal MCF-10A breast cells (IC_50_ = 16.03 ± 0.14 µM), indicating limited selectivity and the need for further optimization to improve its safety profile.^74^

Within the thiazole–triazole hybrid series, small changes in the substituents noticeably shape EGFR activity. Compound 247 benefits from its bulky, electron-rich isatin ring, which strengthens key hydrogen bonds. 253c is the most active molecule, as its hydroxyl group improves solubility and help it fit more effectively into the binding pocket. The 3,5-dicyano group in 258m increases polarity and support stronger interactions with crucial residues, while the p-nitro group in 266d similarly boosts polarity and hydrogen-bonding potential, leading to reliable EGFR engagement.

### Thiazole–indole hybrids

**Scheme 58:**
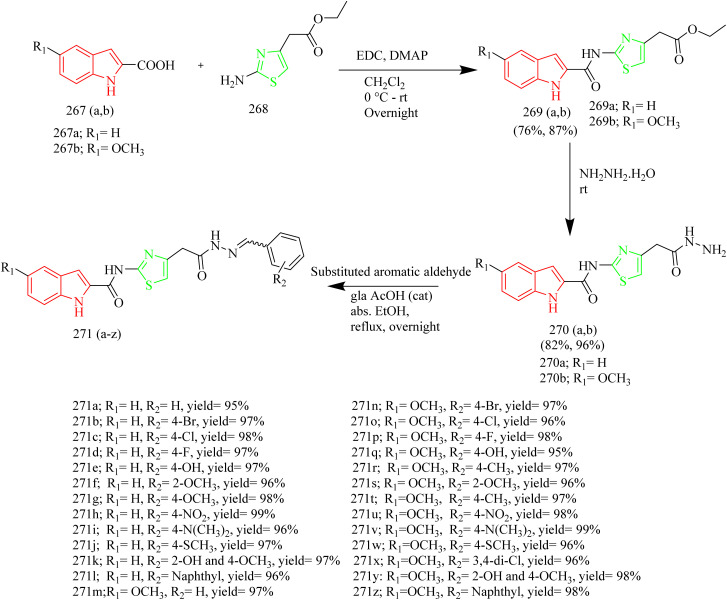
Synthesis of 271(a–z).

**Scheme 59:**
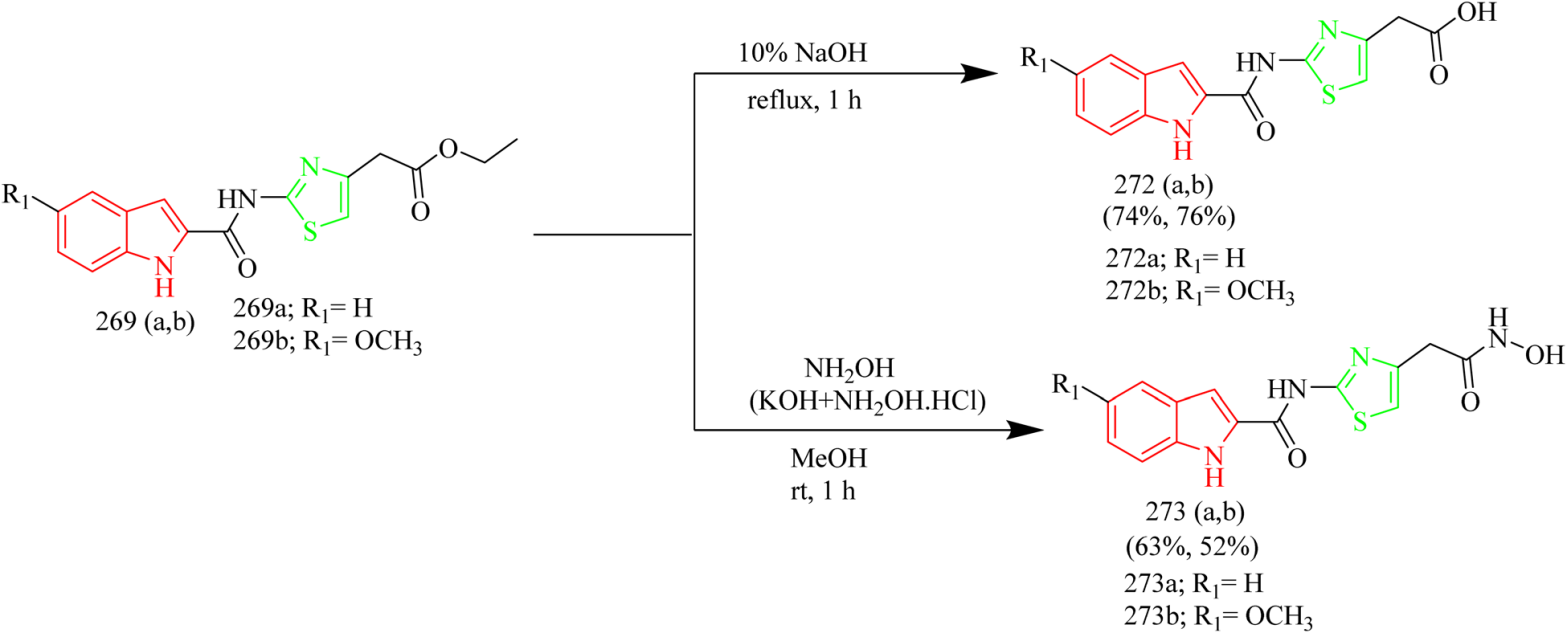
Synthesis of 272(a,b) and 273(a,b).

Saadan *et al.* designed and synthesized thiazolyl-indole-2-carboxamide derivatives as promising multitarget agents for anticancer therapy. The synthesis involved reaction of indole-2-carboxylic acid 267(a,b) with aminothiazole precursor 268 through peptide coupling reaction to yield an amide intermediate 269(a,b). This intermediate upon reaction with hydrazine hydrate yielded intermediate 270(a,b). Finally intermediates 270(a,b) were reacted with various aldehydes to yield the target molecules 271(a–z) (Scheme 58). Further the intermediate 269(a,b) underwent hydrolysis in presence of 10% NaOH to give corresponding acid 272(a,b). Also, thiazole-indole carboxamides 273(a,b) were synthesized *via* reaction of intermediate 264(a,b) with hydroxylamine (Scheme 59). The compound 271i demonstrated potent cytotoxic effects, particularly against MCF-7 (IC_50_ = 6.10 ± 0.4 µM), HeLa cells (IC_50_ = 4.36 ± 0.3 µM), and HCT-116 colon cancer cells (IC_50_ = 9.69 ± 0.8 µM). Moreover, it showed lower toxicity towards normal cells, with an IC_50_ value of 51.26 µM. Additionally, compound 271i exhibited strong EGFR inhibitory activity, with an IC_50_ of 0.063 µM, surpassing the reference drug dasatinib, which showed an IC_50_ of 0.126 µM. Docking analysis revealed that compound 271i established hydrogen bonding interactions with crucial EGFR residues, specifically with Cys797 and Lys745. These results suggest that the synthesized compound 271i has a strong ability to interact with the ATP binding site and effectively inhibit EGFR kinase activity.^75^

**Scheme 60:**
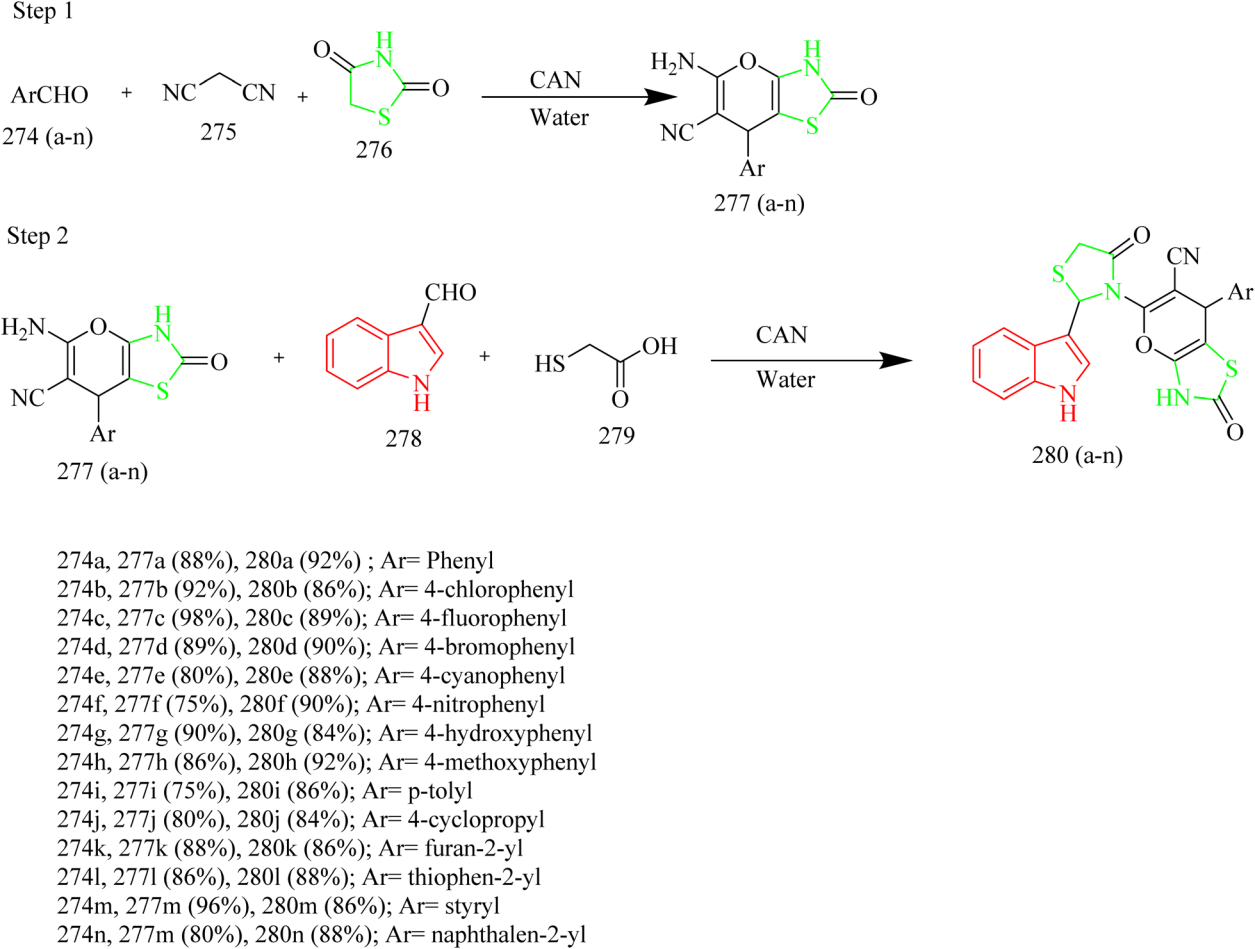
Synthesis of 280(a–n).

Tiwari *et al.* synthesized new indole-thiazole derivatives (Table 6) as EGFR-TK inhibitors, specifically targeting resistance mutations associated with lung cancer therapy. The newly developed compounds 277(a–n) were synthesized following a green chemistry approach. Aromatic aldehydes 274(a–n) were reacted with malonitrile 275 and thiazolidine-2,4-dione 276, resulting in the formation of 277(a–n) derivatives. 277(a–n) Derivatives were further reacted with indole aldehyde 278 and thioglycolic acid 279 leading to the formation of 280(a–n) derivatives (Scheme 60). The compound 276e exhibited potent cytotoxic effects, displaying IC_50_ values of 0.23 µM against HCC827 cells (EGFR Del E746-A750), 0.38 µM against H1975 cells (EGFR L858R/T790M), and 9.87 µM against A549 cells (WT-EGFR). It also displayed low toxicity toward normal BEAS-2B lung cells (IC_50_ > 20 µM), highlighting its favourable selectivity profile. Enzyme assay results showed that compound 280e exhibited reversible inhibition of EGFR L858R/T790M, with an IC_50_ value of 140 nM, closely matching the potency of Osimertinib, which has an IC_50_ of 118 nM. Compound 280e showed strong affinity toward various EGFR isoforms, with binding energies of −4.475 kcal mol^−1^ for WT-EGFR, −6.276 kcal mol^−1^ for EGFR L858R, −8.155 kcal mol^−1^ for EGFR T790M, and −4.769 kcal mol^−1^ for the L858R/T790M double mutant. These results suggest that 280e can effectively bind to the ATP binding pocket, potentially overcoming drug resistance.^76^

**Table 6:** Overview of EGFR inhibition activity of thiazole–indole hybrids

Compound	Key substituent	Major interactions	EGFR IC_50_ (µM)	SAR observation
271i	*p*-dimethylamine group on the benzylidene ring	Hydrogen bonding with Cys797 and Lys745	0.063	The *para*-dimethylamine group increases the electron density and polarity of the molecule enabling favorable interactions within the EGFR ATP binding site
280e	*p*-cyanophenyl substitution	Hydrogen bonding with key residues like Lys745, Met793, and Thr854 in EGFR mutants	140 nM	Electron withdrawing cyano group enhances polarity and hydrogen bonding

Within the thiazole–indole hybrid series, substituent effects strongly influence EGFR inhibition. Compound 271i, which contains a *p*-dimethylamine group, is the most active showing an IC_50_ of 0.063 µM. This substituent increases the molecule's electron density and polarity, helping it to form strong, stable hydrogen bonds with key EGFR residues like Cys797 and Lys745. Meanwhile, compound 280e, with its *p*-cyanophenyl group, also delivers strong activity especially against EGFR mutants (IC_50_ = 140 nM). The cyano group withdraws electrons, increasing polarity and supporting effective interactions with residues such as Lys745, Met793, and Thr854. Overall, both electron-rich and electron-deficient groups help tune the molecule's polarity, allowing it to engage the receptor more effectively and strengthen its binding interactions.

### Thiazole–thiophene hybrids

**Scheme 61:**
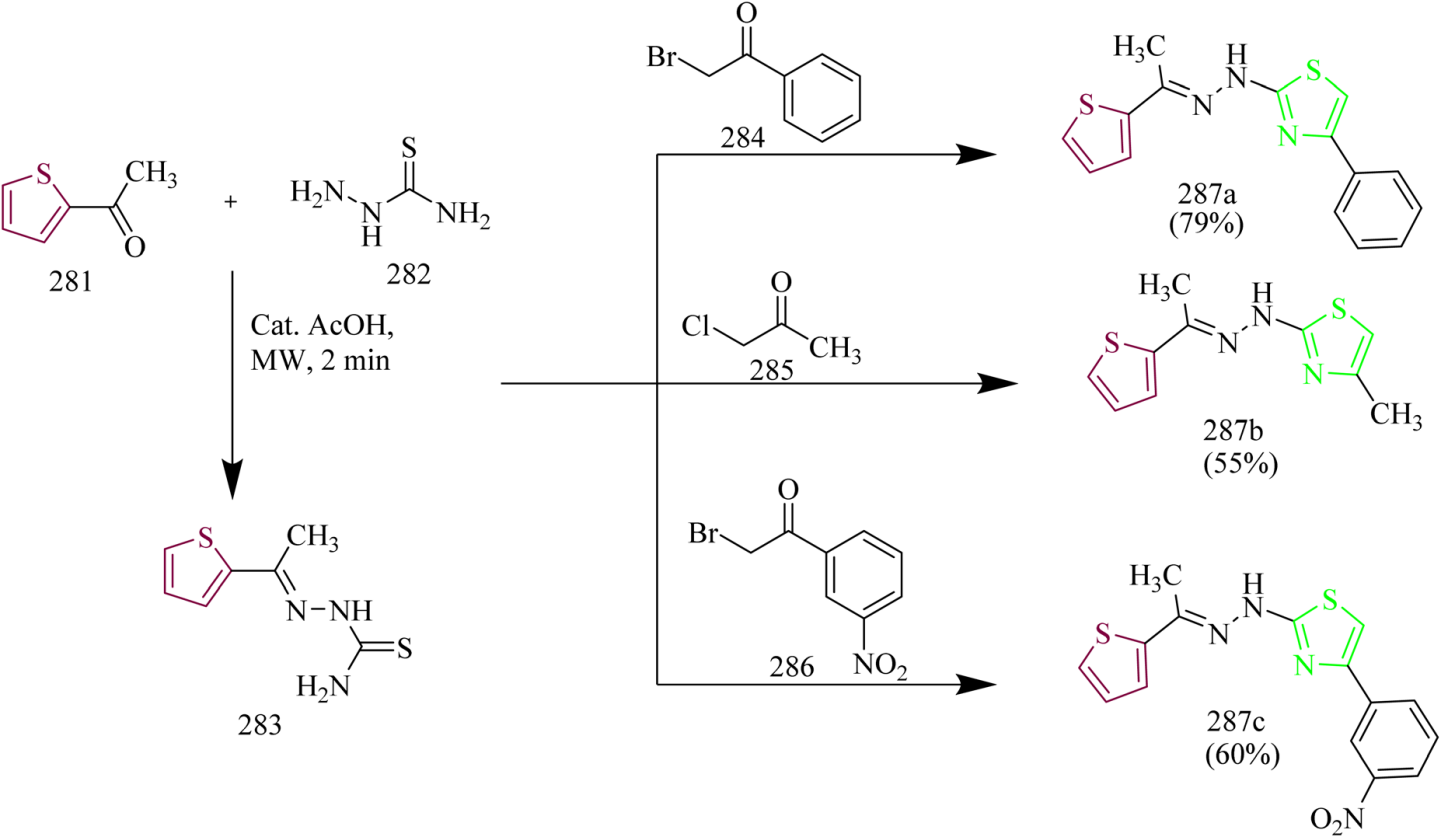
Synthesis of 283, 287a, 287b, and 287c.

**Scheme 62:**
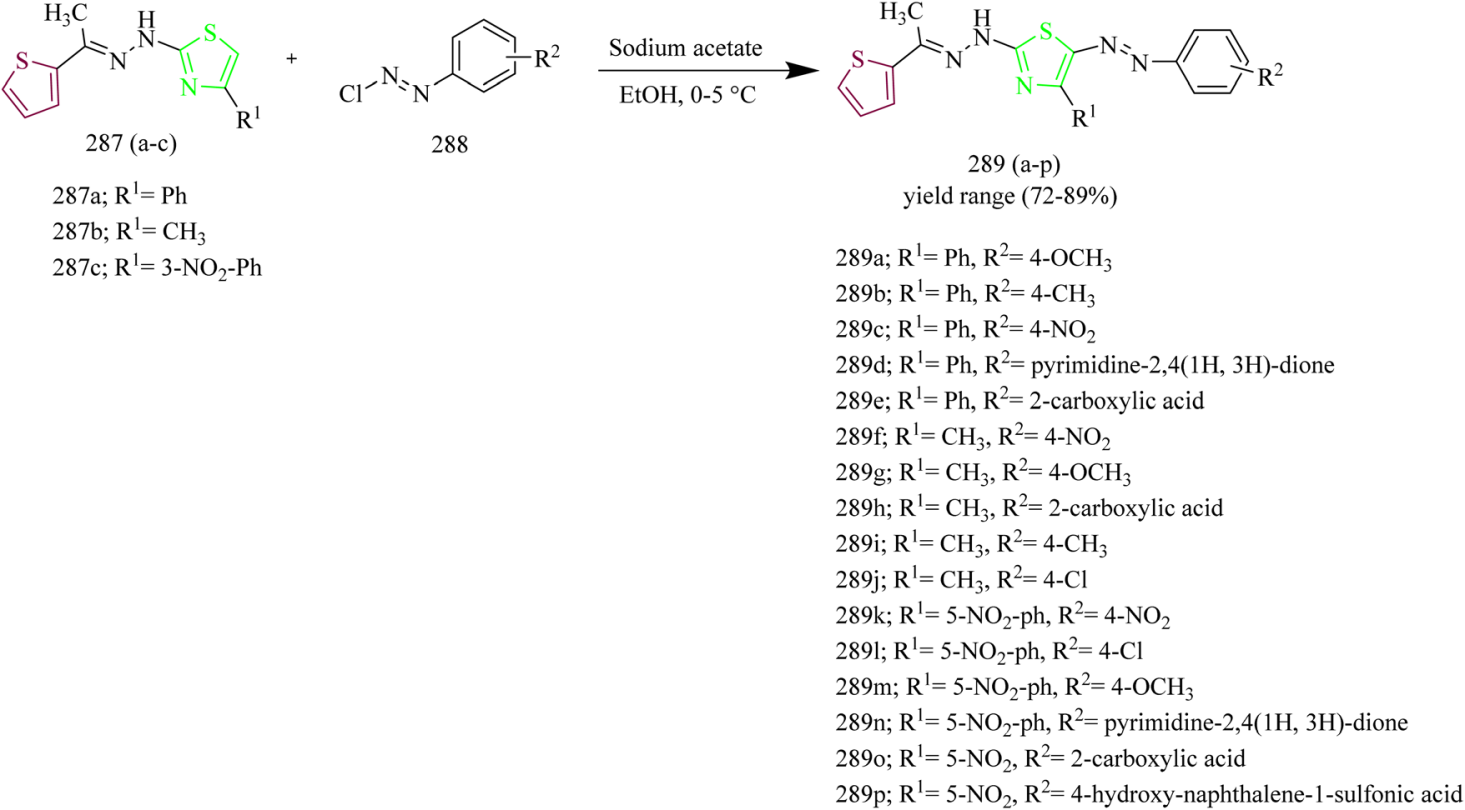
Synthesis of 289(a–p).

El-Naggar *et al.* carried out the rational design, chemical synthesis, and docking investigations of a new series of hydrazinyl–thiazole thiophene hybrid molecules. A set of ethyidene-hydrazinyl-thiazole derivatives, named as 287(a–c), was synthesized by following the steps outlined in (Scheme 61). Initially, 2-acetylthiophene 281 was condensed with thiosemicarbazide 282 to produce ethylidenethiosemicarbazone 283. This intermediate then underwent cyclisation with different α-halocarbonyl compounds like phenacyl bromide 284, chloroacetone 285, and m-nitrophenacyl bromide 286 in presence of 2 ml of acetic acid, resulting in the formation of thiazole–thiophene hybrids 287(a–c) (Scheme 61, Table 7). Subsequently, the intermediates 287(a–c) were treated with different arenediazonium salts 288 under ethanol reflux conditions using sodium acetate as a base, leading to the formation of the desired azo derivatives incorporating thiazole–thiophene hybrids, labelled as 289(a–p) (Scheme 62). Compound 289j demonstrated potent and selective anticancer activity, particularly against MCF-7 and HepG-2 (liver cancer) cell lines, with IC_50_ values of 6.73 µM and 10.87 µM respectively. These results reflect stronger cytotoxic effects compared to the standard drug roscovitine, which showed IC_50_ values ranging from 9.32 to 13.82 µM. In addition to this, compound 289j showed strong inhibitory activity against EGFR, achieving an IC_50_ value of 82.8 nM, which is comparable to that of the reference drug erlotinib (IC_50_ = 62.4 nM). SAR analysis revealed that incorporation of 4-chlorophenyl group at the 3-position of the thiazole ring in compound 289j led to a marked improvement in both cytotoxic and enzyme inhibitory activities. Compound 289j was found to exhibit stronger binding interactions compared to erlotinib, showing nearly equivalent binding energies. It was found to form hydrogen and hydrophobic bonds with methionine, and it interacted with additional residues like Glu738 of the EGFR enzyme making it a promising inhibitor for the further research.^77^

**Table 7:** Overview of EGFR inhibition activity of thiazole–thiophene hybrids

Compound	Key substituent	Major interactions	EGFR IC_50_ (µM)	SAR observation
289j	*p*-chlorophenyl thiazole derivative	Hydrogen bonding and hydrophobic interaction with Met793 and Glu738	82.8 nM	*p*-chlorophenyl group was found to increase lipophilicity helping the molecule to fit well into the hydrophobic pocket of the EGFR enzyme

Thiazole–thiophene hybrids work well as EGFR inhibitors because the thiazole ring helps the molecule to fit into the hinge region through hydrogen bonds. The thiophene ring adds strong hydrophobic interactions, allowing the compound to bind into the ATP pocket. Adding different groups especially at para position of a phenyl or heteroaryl ring can tune the polarity and binding of the molecule. Overall, the strategical placement of the substituents can lead to strong EGFR inhibition in the series.

### Thiazole–pyridine hybrids

**Scheme 63:**
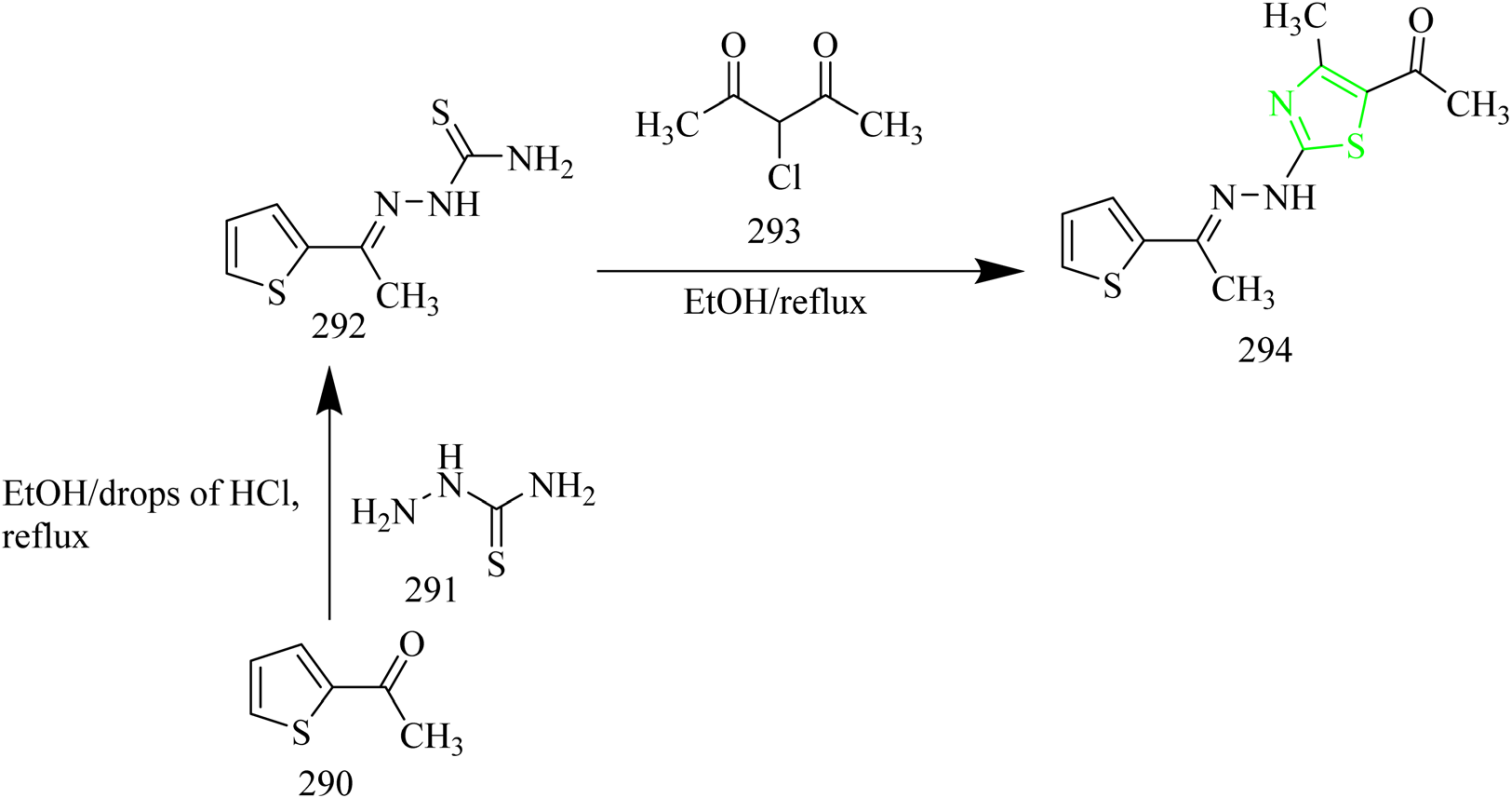
Synthesis of 294.

**Scheme 64:**
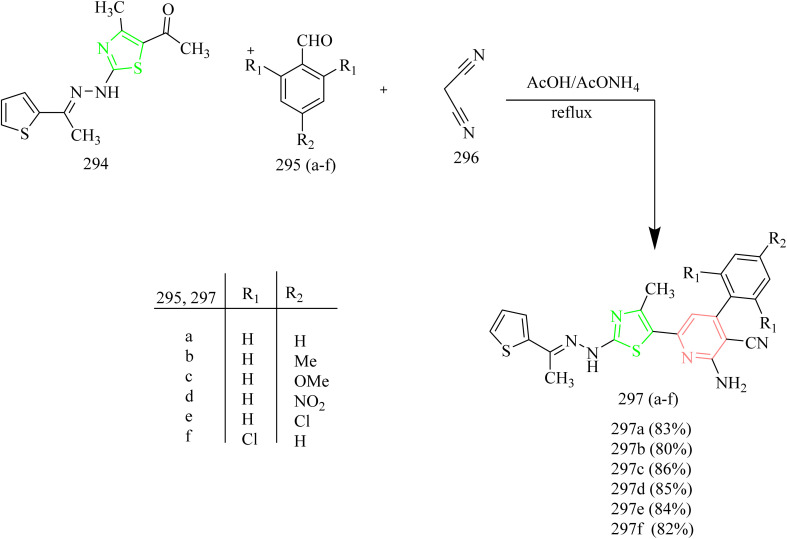
Synthesis of 297(a–f).

Ashmawy and coworkers carried out the synthesis of novel thiophenyl thiazolyl–pyridine hybrids (Table 8). The synthetic route involved the condensation of thiophene ketone 290 and thiosemicarbazide 291 resulting in the formation of the carbothioamide intermediate 292. This intermediate was subsequently reacted with 3-chloropentane-2,4-dione 293 to produce the thiazole–thiophene based hybrid intermediate 294 (Scheme 63). Subsequently, compound 294 was reacted with various benzaldehyde derivatives 295(a–f) and malononitrile 296, leading to the formation of a novel series of thiazolyl-pyridine derivatives bearing a thiophene moiety, designated as compounds 297(a–f) (Scheme 64). Among the synthesized compounds 297(a–f), compound 297e emerged as the most potent compound, showing superior cytotoxic activity against A549 lung cancer cells with an IC_50_ value of 0.302 µM, outperforming the standard drug doxorubicin which has an IC_50_ value of 0.460 µM. This remarkable efficacy is likely due to the fusion of thiazole, thiophene, pyridine moieties. From docking analysis, it was found that the compound 297e showed a high affinity for the EGFR tyrosine kinase domain with a binding value of −24.45 kcal mol^−1^. It primarily interacted with the Lys721 and Arg817 residue of EGFR enzyme through arene–cation interactions. From SAR analysis, it was found that adding electron-withdrawing groups, such as chlorine in case of compound 297e, increased the activity. Also replacing the phenyl ring with a pyridine ring greatly enhanced cytotoxicity compared to the intermediate 294. Additionally, the inclusion of thiophene ring improved the membrane permeability and target interaction.^78^

**Table 8:** Overview of EGFR inhibition activity of thiazole–pyridine hybrids

Compound	Key substituent	Major interactions	EGFR IC_50_ (µM)	SAR observation
297e	Chlorophenyl substitution	Arene cation interaction with Lys721 and Arg817	—	Electron withdrawing substituents improved the activity, and swapping the phenyl ring for a pyridine ring led to a marked increase in cytotoxic potency

Thiazole–pyridine hybrids are gaining attention as effective EGFR tyrosine kinase inhibitors due to their capacity to form diverse non-covalent interactions within the enzyme's active site. Incorporating electron-withdrawing substituents like –NO_2_, –CN, or –CF_3_ on the pyridine or phenyl rings enhances their inhibitory potential by increasing molecular polarity and promoting hydrogen bonding with critical residues such as Met793, Lys745, and Asp831. The thiazole ring contributes structural rigidity and planarity, aiding in optimal alignment within the ATP-binding cleft. Additionally, the nitrogen atom in the pyridine ring serves as a hydrogen bond acceptor, further stabilizing interactions with polar amino acids in the EGFR binding domain.

### Thiazole–pyrimidine hybrids

**Scheme 65:**
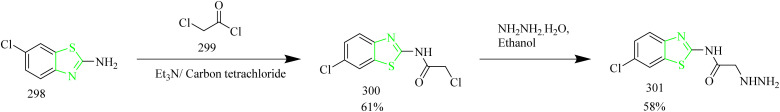
Synthesis of 301.

**Scheme 66:**
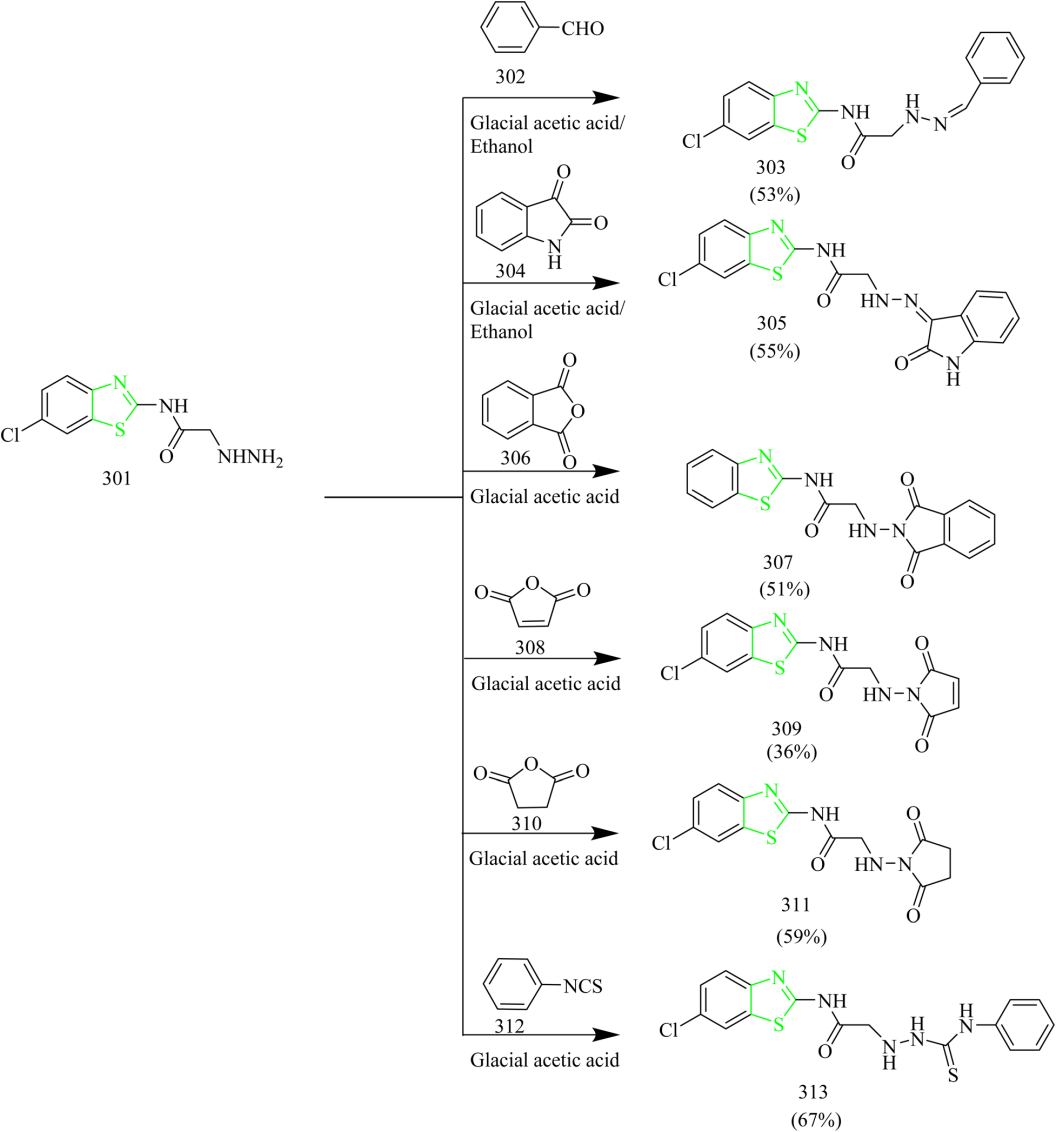
Synthesis of 303, 305, 307, 309, 311, 313.

**Scheme 67:**
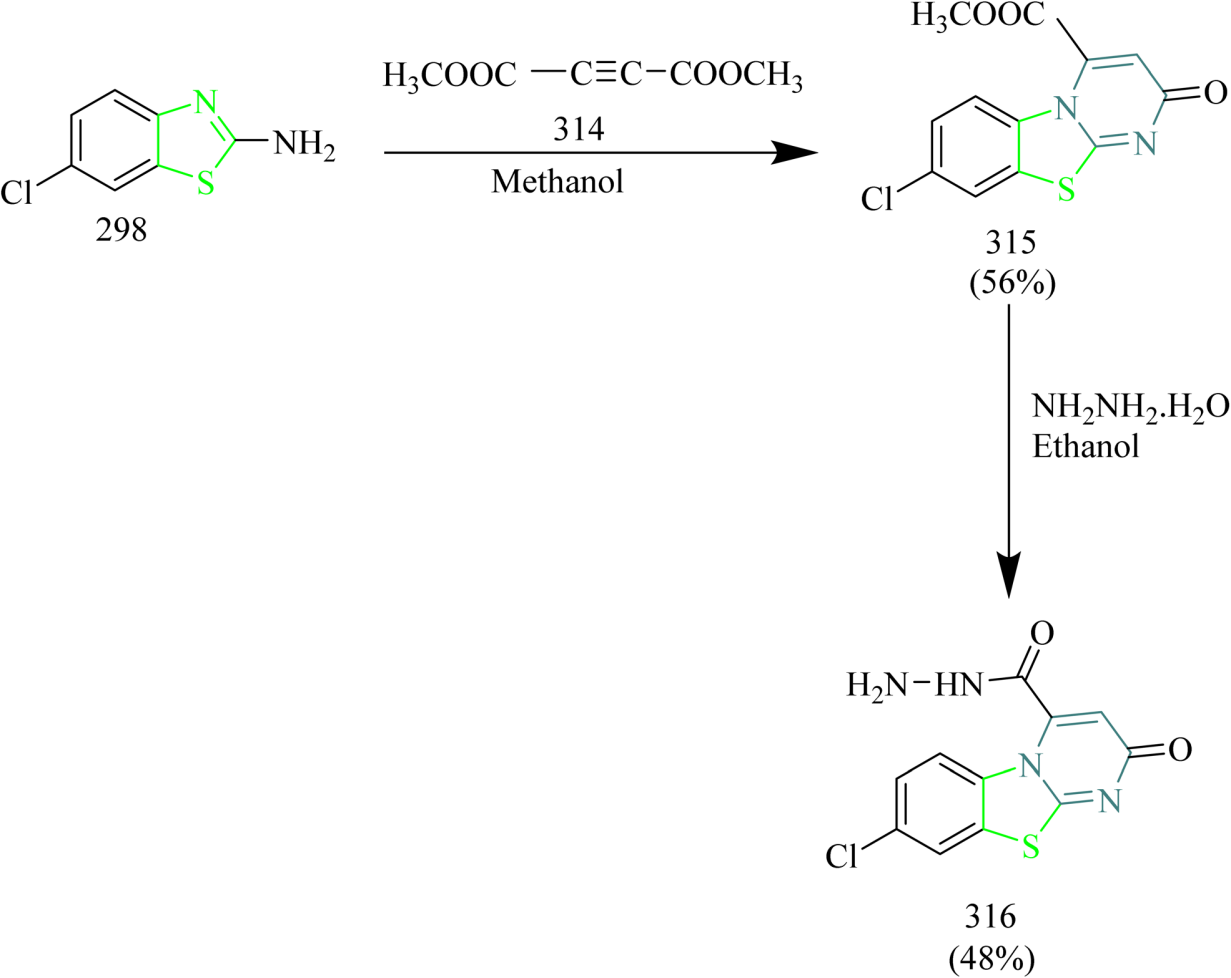
Synthesis of 316.

**Scheme 68:**
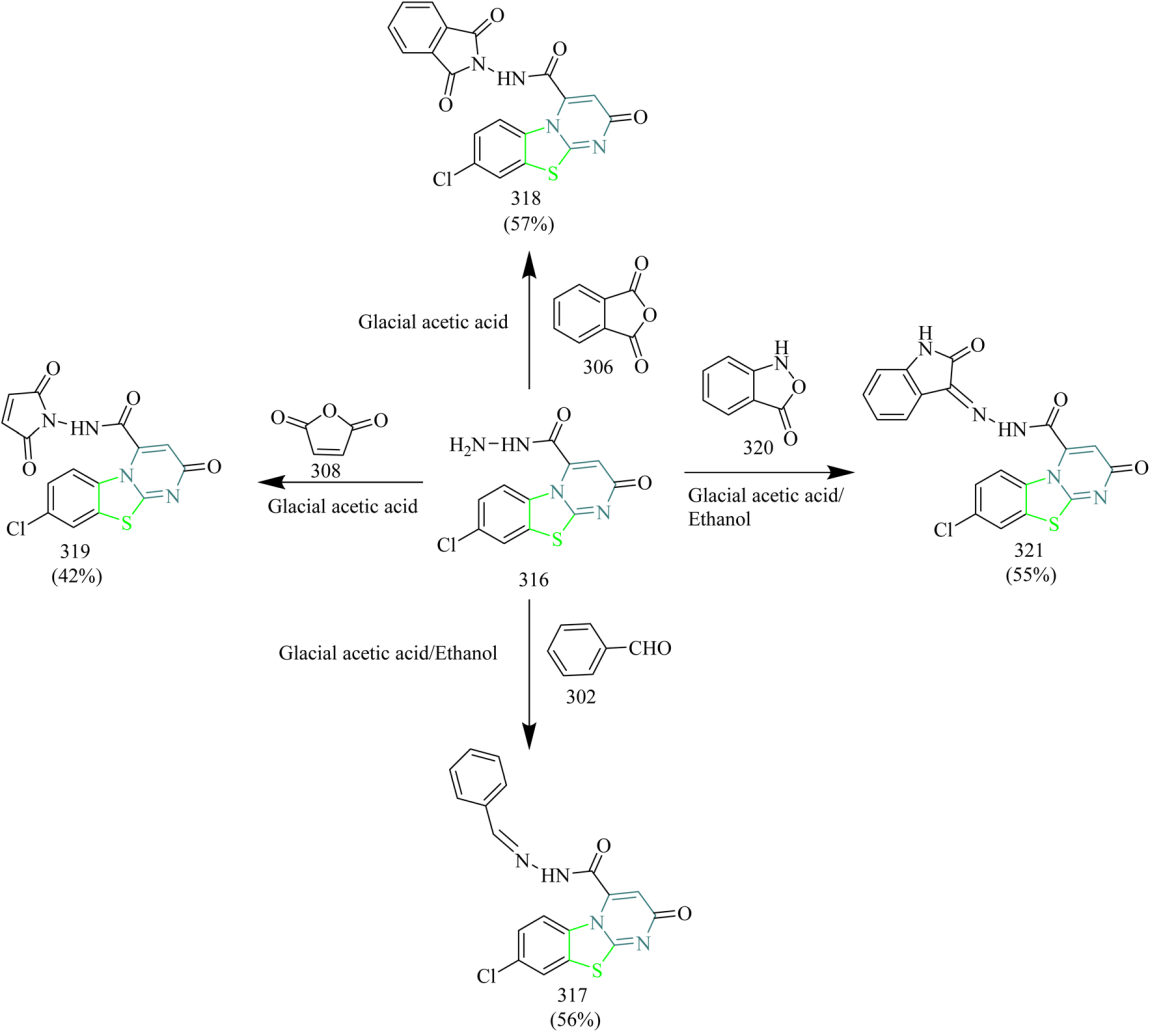
Synthesis of 317, 318, 319, and 321.

Pyrimido–benzothiazole hybrid compounds were synthesized by Gabr *et al.* and evaluated their antitumor potential, with a focus on EGFR tyrosine kinase inhibition. The synthetic strategy commenced with the transformation of 2-amino-6-chlorobenzothiazole 298 into the corresponding chloroacetamide derivative 300*via* acylation with chloroacetyl chloride 299 in carbon tetrachloride and triethylamine. Subsequent treatment of compound 300 with hydrazine hydrate in ethanol afforded the key hydrazide intermediate 301 (Scheme 65). Reaction of 301 with benzaldehyde 302 or isatin 304 in ethanol in the presence of glacial acetic acid furnished the Schiff base derivatives 303 and 305, respectively. Furthermore, cyclocondensation of 301 with phthalic, maleic, or succinic anhydride under acidic conditions produced the corresponding acetamide analogues 307, 309, 311 while condensation with phenyl isothiocyanate in ethanol yielded the thiourea derivative 313. A parallel synthetic pathway involved cyclization of 2-amino-6-chlorobenzothiazole 298 with dimethyl acetylenedicarboxylate 314 in methanol, providing the methyl pyrimido[2,1-*b*]benzothiazole-4-carboxylate 315 as the sole isolable regioisomer. Hydrazinolysis of 315 delivered the hydrazide 316, which was subsequently diversified through additional condensation reactions (Scheme 66). Treatment of 316 with phthalic or maleic anhydride afforded the corresponding carboxamide derivatives 318 and 319 (Scheme 67). Finally, reaction of 316 with isatin or benzaldehyde in ethanol and glacial acetic acid produced the hydrazone derivatives 317 and 321 respectively (Scheme 68). Among the synthesized compounds, the compound 311 showed highest inhibition in case of breast cancer (MDA-MB-468), non-small cell lung cancer (NCI-H522), renal cancer (A498), and leukemia (RPMI-8226) with the GI% values of 62.5%, 56.5%, 46.6%, and 44%. Compound 311 exhibited the strongest EGFR-TK inhibition in the series, achieving 40.33% activity, which was consistent with its overall biological performance. This result indicates that introducing the 2,5-dioxopyrrolidine fragment onto the benzothiazole scaffold significantly enhanced its binding affinity toward EGFR-TK.^79^

**Scheme 69:**
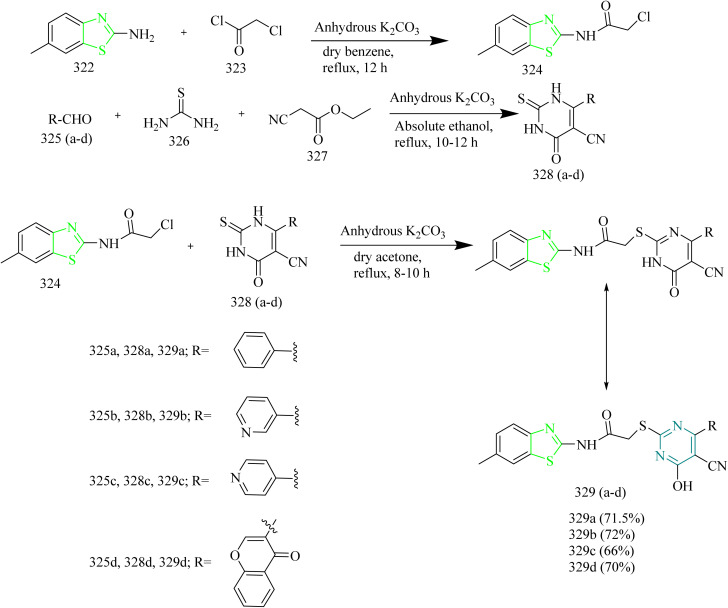
Synthesis of 329(a–d).

**Scheme 70:**
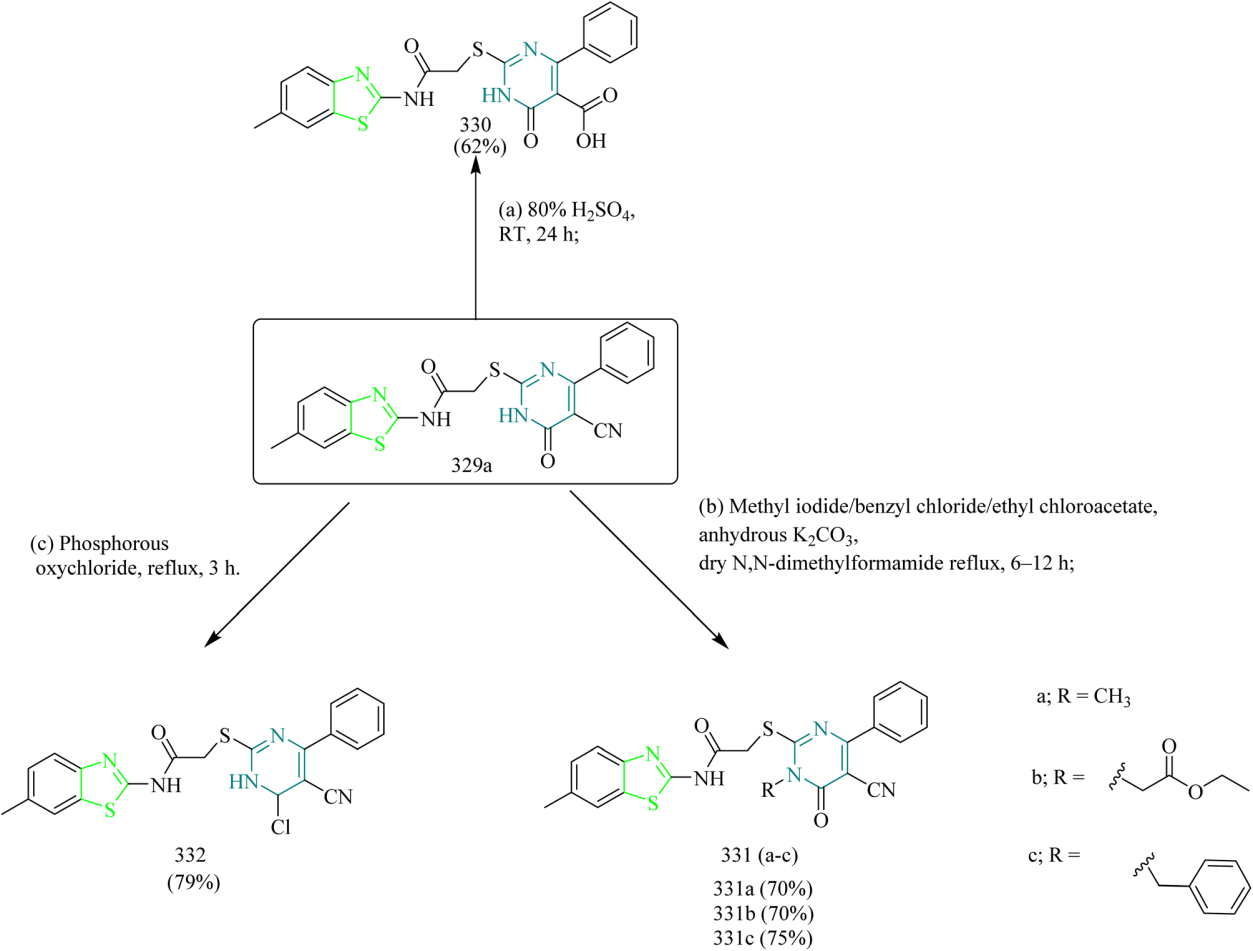
Synthesis of 330, 331(a–c), and 332.

**Scheme 71:**
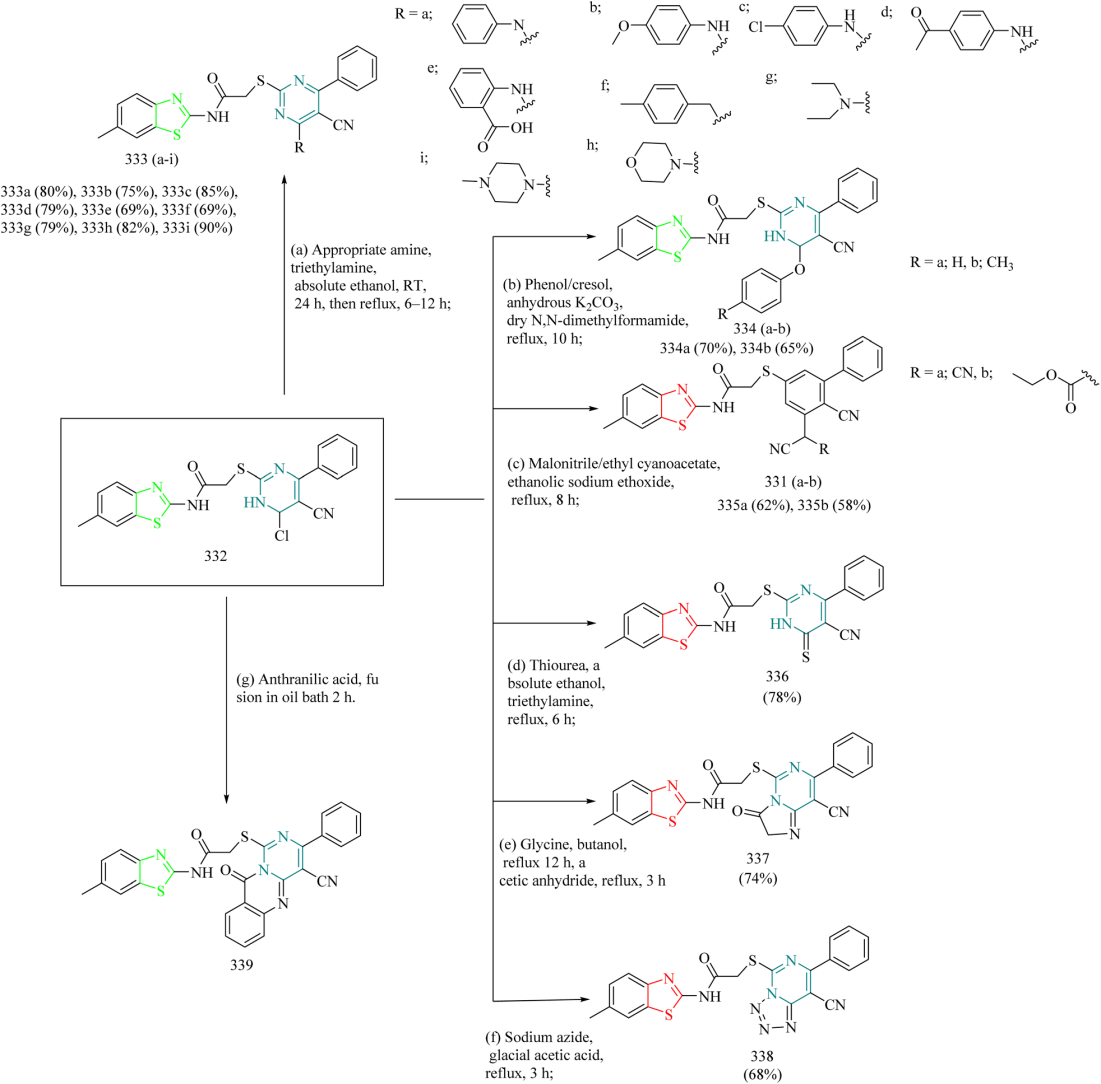
Synthesis of 333(a–i), 334(a,b), 335(a,b), 336, 337, 338, and 339.

**Scheme 72:**
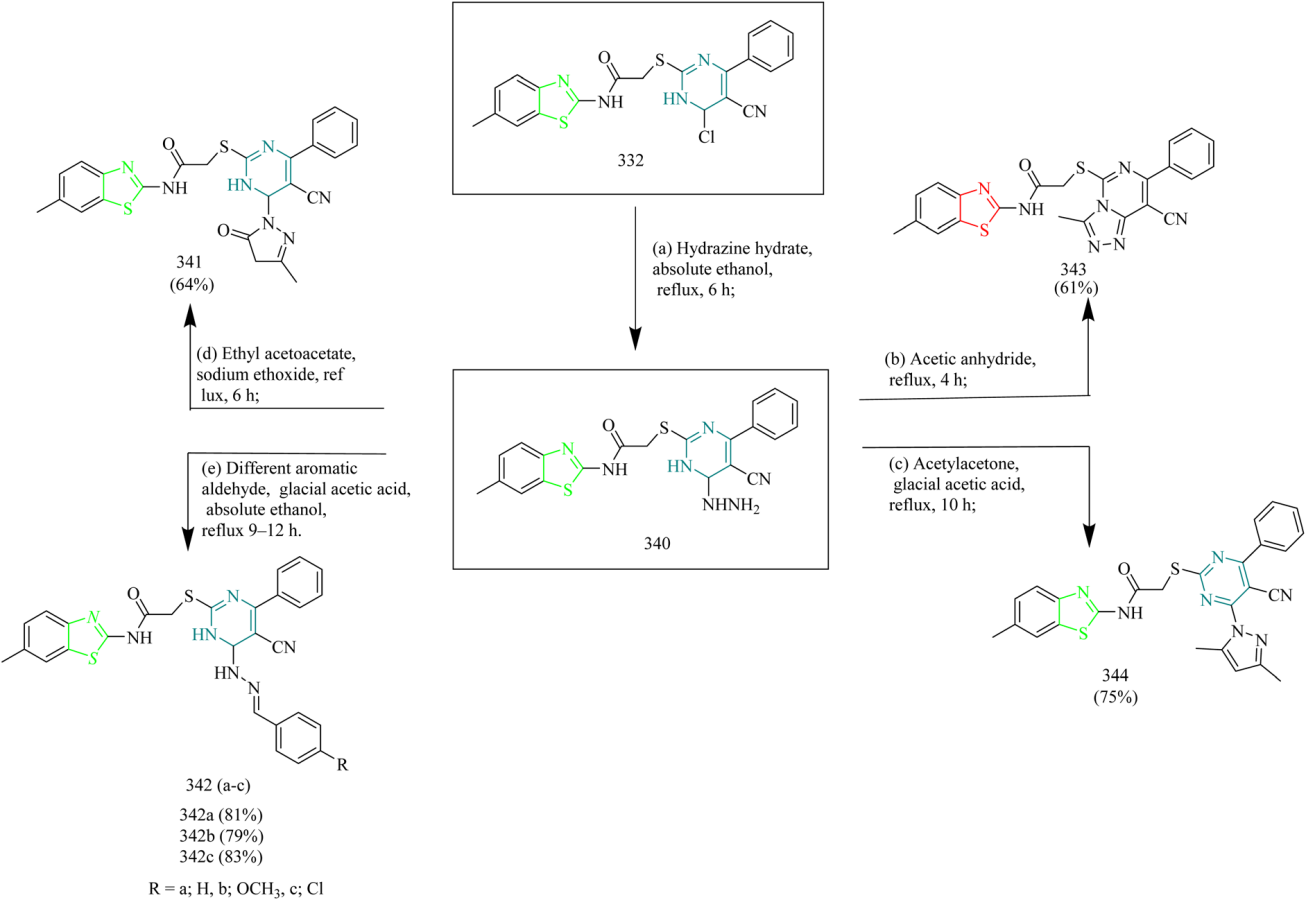
Synthesis of 340, 341, 342(a–c), 343, 344.

Abdellatif and coworkers synthesized a novel series of benzothiazole hybrids incorporating a pyrimidine moiety and evaluated their antiproliferative activity. The synthetic pathway began with the reaction of benzoaminothiazole 322 and chloroacetyl chloride 323 resulting in the formation of thiazole-acetamide intermediate 324. Separately, various aromatic aldehydes 325(a–d) were condensed with thiourea 326 and ethyl cyanoacetate 327 in ethanol under basic conditions to produce 2-thioxopyrimidine-5-carbonitrile derivatives 328(a–d). In the final step, intermediate 324 was coupled with compounds 316(a–d) in ethanol under basic conditions to yield the target thiazole–pyrimidine hybrids 328(a–d) (Scheme 69 and Table 9). Then the nitrile group at C5 position of compound 329a was hydrolyzed using 80% sulfuric acid, resulting in the formation of the corresponding carboxylic acid derivative 330. Compound 329a underwent *N*-alkylation with methyl iodide, benzyl chloride, or ethyl chloroacetate in the presence of anhydrous potassium carbonate as a catalyst and *N*,*N*-dimethylformamide as the solvent, yielding derivatives 331(a–c), respectively. Additionally, compound 329a was chlorinated by refluxing with excess phosphorus oxychloride, leading to the formation of the 4-chloropyrimidine derivative 332 (Scheme 70). Compounds 333(a–i) were obtained by reacting compound 332 with various primary or secondary amines. Compounds 334(a,b) were obtained by condensing compound 332 with phenol and cresol, respectively. The chlorine atom at the 4-position of compound 332 was displaced through nucleophilic substitution using either malonitrile or ethyl cyanoacetate, yielding compounds 335(a,b). Furthermore, compound 332 underwent substitution of the chlorine atom with a mercapto group upon treatment with thiourea leading to the formation of compound 336. The reaction of compound 332 with glycine in butanol, followed by intramolecular cyclization, produced the imidazopyrimidine derivative 337. Refluxing compound 332 with sodium azide afforded the tetrazole derivative 334. Additionally, compound 327 was synthesized by fusing anthranilic acid with the chloro derivative 320, as illustrated in (Scheme 71). Hydrazinolysis of compound 332 using 98% hydrazine in refluxing ethanol resulted in the formation of the hydrazinopyrimidine derivative 340. Subsequent reactions of compound 332 with acetic anhydride, acetylacetone, and ethyl acetoacetate yielded compounds 341, 342, and 343, respectively. The target hydrazone derivatives 344(a–c) were synthesized by condensing the hydrazine derivative of compound 332 with various substituted aromatic benzaldehydes, as shown in (Scheme 72). Among these, compound 329d exhibited potent cytotoxicity against MCF-7, with an IC_50_ value of 0.693 µM, which was superior to that of erlotinib (IC_50_ = 2.32 µM). It also showed notable growth inhibition in non-small cell lung cancer (HOP-62) and renal cancer (UO-31) cell lines, achieving over 30% inhibition. Against EGFR, compound 329d demonstrated an IC_50_ of 0.203 µM, comparable to that of lapatinib (IC_50_ = 0.205 µM). Molecular docking studies confirmed a strong binding affinity, with a favorable docking score of −11.31 kcal mol^−1^. The compound established key hydrogen bonds with amino acid residues in the hinge region.^80^

**Table 9:** Overview of EGFR inhibition activity of thiazole–pyrimidine hybrids

Compound	Key substituent	Major interactions	EGFR IC_50_ (µM)	SAR observation
311	2,5-Dioxopyrrolidine moiety on benzothiazole scaffold	Hydrophobic interaction with Met742, Val702, Asp831, Leu764, Lys721, Thr766, Ile765, Ala719 and hydrogen bond with Lys721, Asp831	—	Saturation of the olefinic bond within the 2,5-dioxopyrrolidine ring markedly enhanced the hydrophobic interaction capability
329d	Chromone moiety on pyrimidine ring	Hinge region hydrogen bonding with Met793	0.203	The chromone ring system strengthened π–π interactions within the binding site which in turn contributed to the increased potency of compound
347	Fused thiazolopyrimidine scaffold	Hydrogen bond with Asp800, Asp855, Lys745 and π–H interaction with Leu718	233.87	Cyclisation of the pyrimidinethione into a 6,5-fused bicyclic system makes the molecule more rigid and planar, strengthening π-conjugation and electron delocalization which helps in effective EGFR binding
363b	*p*-methoxyphenyl substitution on pyridine ring	Hydrogen bond with Met769, Gln767, Thr766, Asp831, and Ala717	0.35	The *p*-methoxyphenyl group enhanced the alignment in the EGFR pocket by enabling hydrogen bonding, which in turn positioned thiazole sulfur to interact efficiently within the EGFR site

**Scheme 73:**
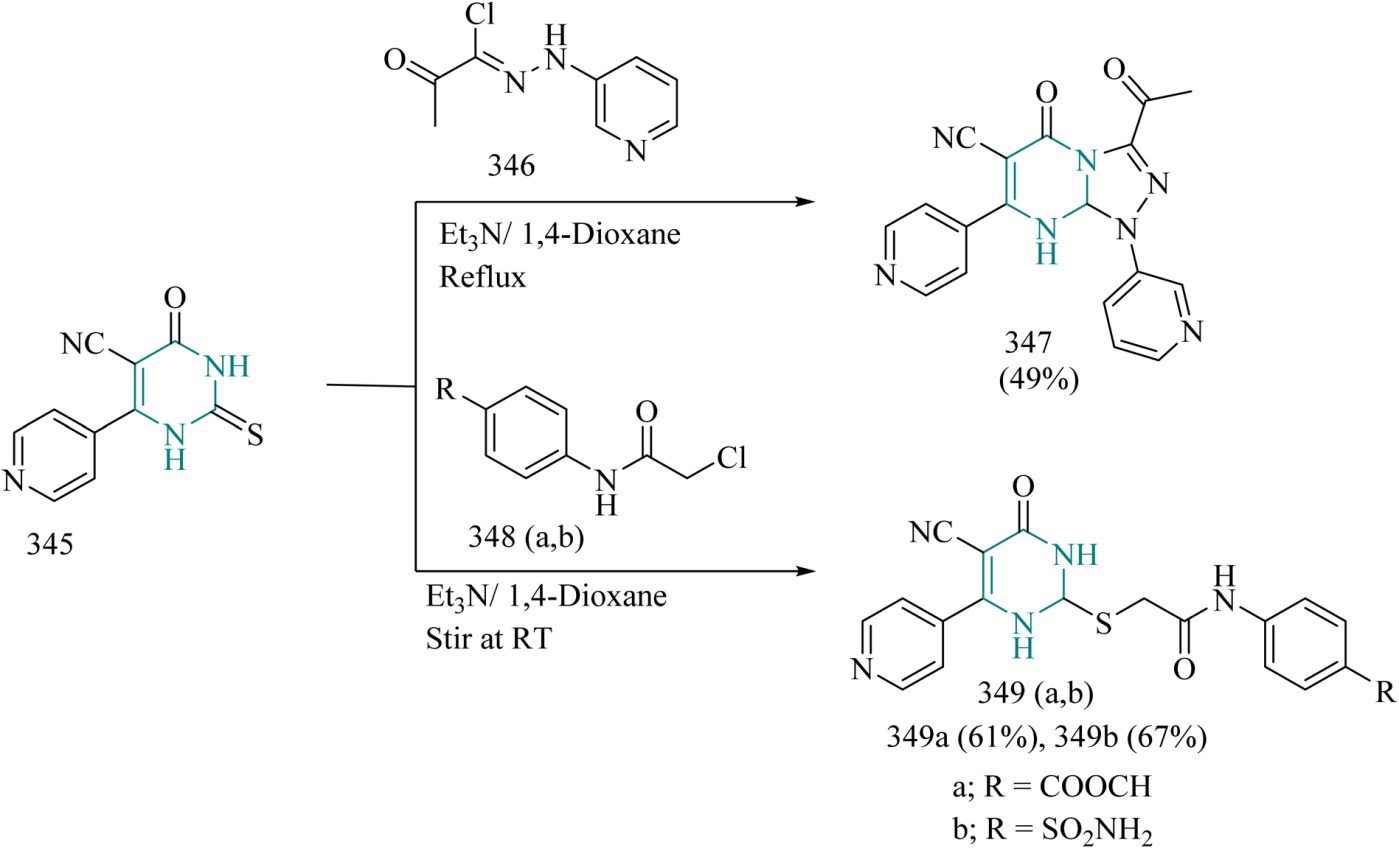
Synthesis of 347 and 349(a,b).

**Scheme 74:**
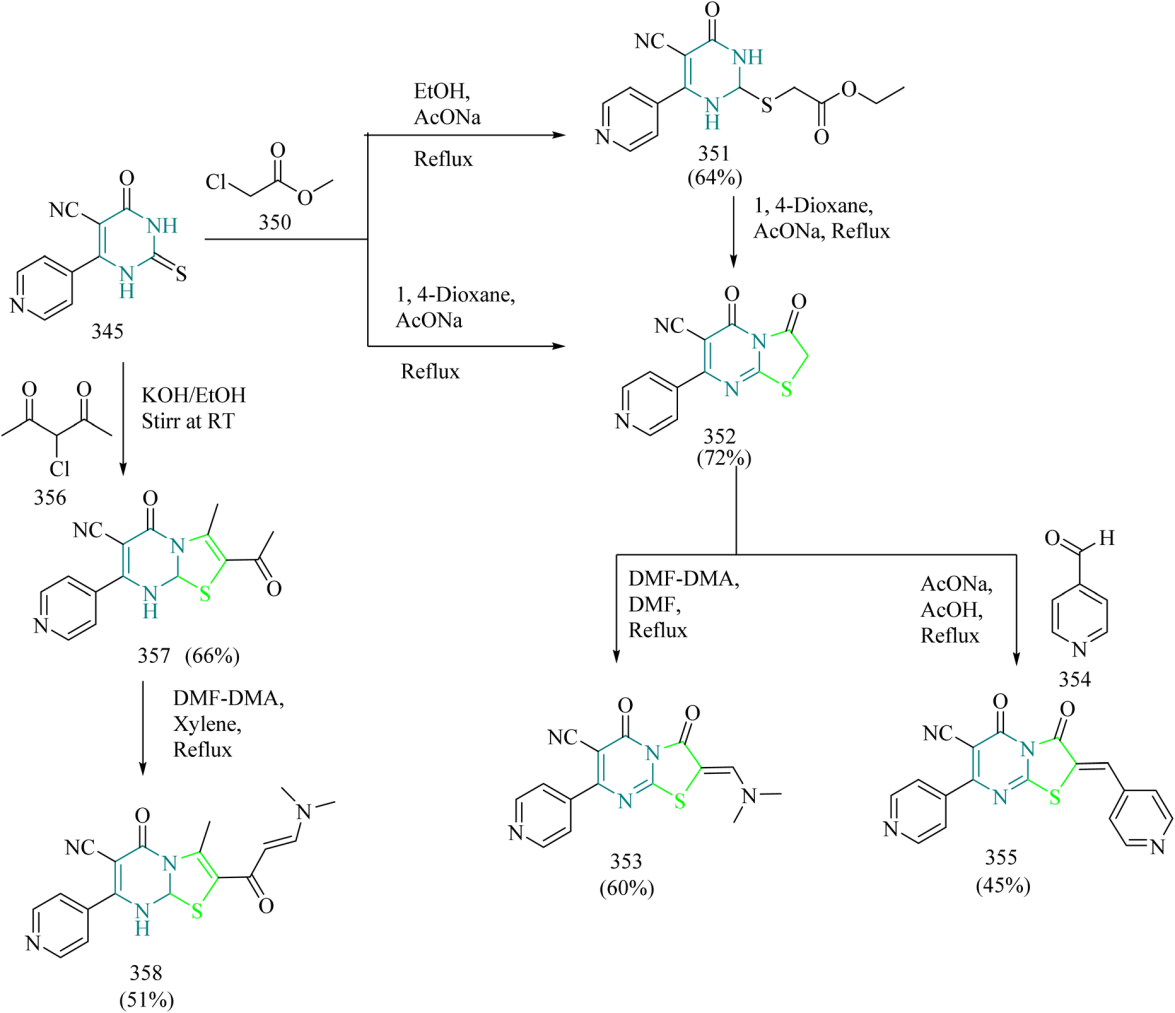
Synthesis of 353, 355, and 358.

Abdelrahman *et al.* carried out the design and synthesis of thiazolo–pyrimidine hybrids, supported by computational studies to evaluate their potential as EGFR inhibitors. The study also explored their antiproliferative activity to assess their effectiveness as anticancer agents. The reaction of pyrimidinethione 345 with hydrazonoyl chloride 346 led to the formation of triazolopyrimidine derivative 347 (Scheme 73). Alternatively, stirring pyrimidinethione 345 with chloroacetamide derivatives 348(a,b) yielded the corresponding S-alkylated compounds 349(a,b) (Scheme 73). Treatment of pyrimidinethione 345 with ethyl chloroacetate 350 resulted in the formation of the S-alkylated derivative 351. However, carrying out the reaction in 1,4-dioxane led to the formation of an intramolecular 5-*exo*-trig cyclisation, leading to the formation of the thiazolopyrimidine ring system 352. Compound 352 was likewise synthesized by refluxing compound 345 in 1,4-dioxane in the presence of anhydrous sodium acetate. Furthermore, condensation of compound 352 with DMF-DMA or 4-formylpyridine 354 resulted in the formation of α,β-unsaturated carbonyl derivatives 353 and 355, respectively. Reaction of pyrimidinethione 345 with α-chloroacetylacetone 356 in ethanolic potassium hydroxide led to the formation of thiazolopyrimidine derivative 357. The acetyl group in compound 357 was then subjected to treatment with DMF-DMA under reflux in xylene, resulting in the formation of enaminone derivative 358 (Scheme 74). Compound 347 exhibited IC_50_ values of 7.21 ± 3.0 µM against MCF-7 and 9.15 ± 1.9 µM against HCT116 colorectal cancer cells. Similarly, compound 355 demonstrated IC_50_ values of 7.54 ± 0.2 µM for MCF-7 and 9.11 ± 0.5 µM for HCT116, confirming their notable antiproliferative effects. Furthermore, compounds 347 and 355 were assessed for their EGFR inhibitory potential and were found to be effective EGFR inhibitors. Compound 347 exhibited an IC_50_ value of 495.24 ± 18.68 µM, while compound 355 showed a stronger inhibition with an IC_50_ of 233.87 ± 8.82 µM. Both compounds outperformed the reference drug erlotinib, which displayed an IC_50_ value of 809.26 ± 30.53 µM. Molecular docking studies using PDB ID: 3W32 (breast cancer protease) demonstrated that compound 347 exhibited strong binding affinity toward the EGFR kinase domain, with a BE of −8.0989 kcal mol^−1^ compared to roscovitine (−8.6504 kcal mol^−1^) and W32 (co-crystallized ligand) (−9.8047 kcal mol^−1^). The molecule formed four hydrogen bonds with essential amino acid residues. Notably, it contains two strategically positioned carbonyl groups (acting as side-chain acceptors) that chelate with Lys745, while the pyridine and nitrile nitrogen atoms interact with Met793 and Asp855, respectively. These interactions suggest that its dual potential as an EGFR inhibitor and a DNA intercalator.^81^

**Scheme 75:**
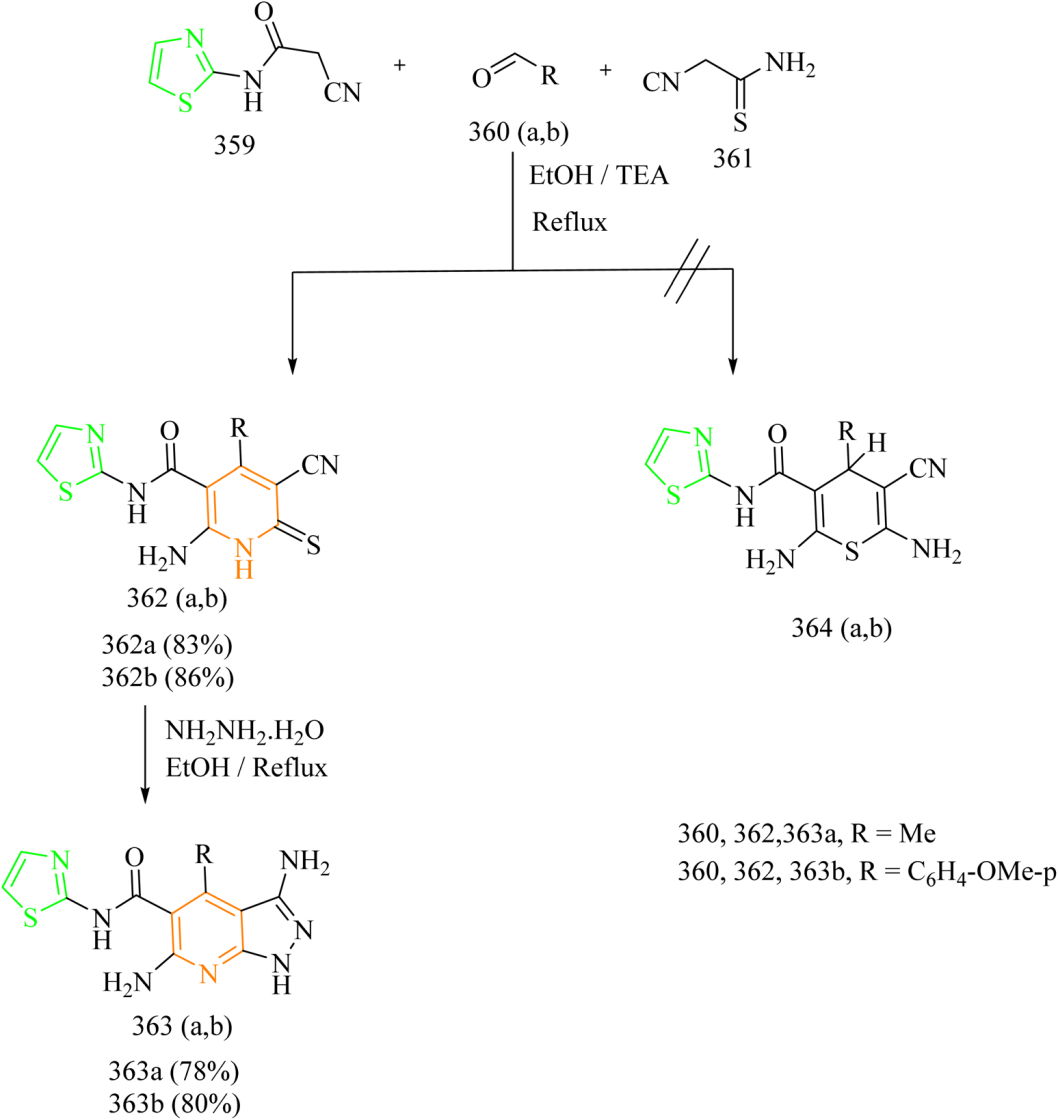
Synthesis of 363(a,b) and 364(a,b).

**Scheme 76:**
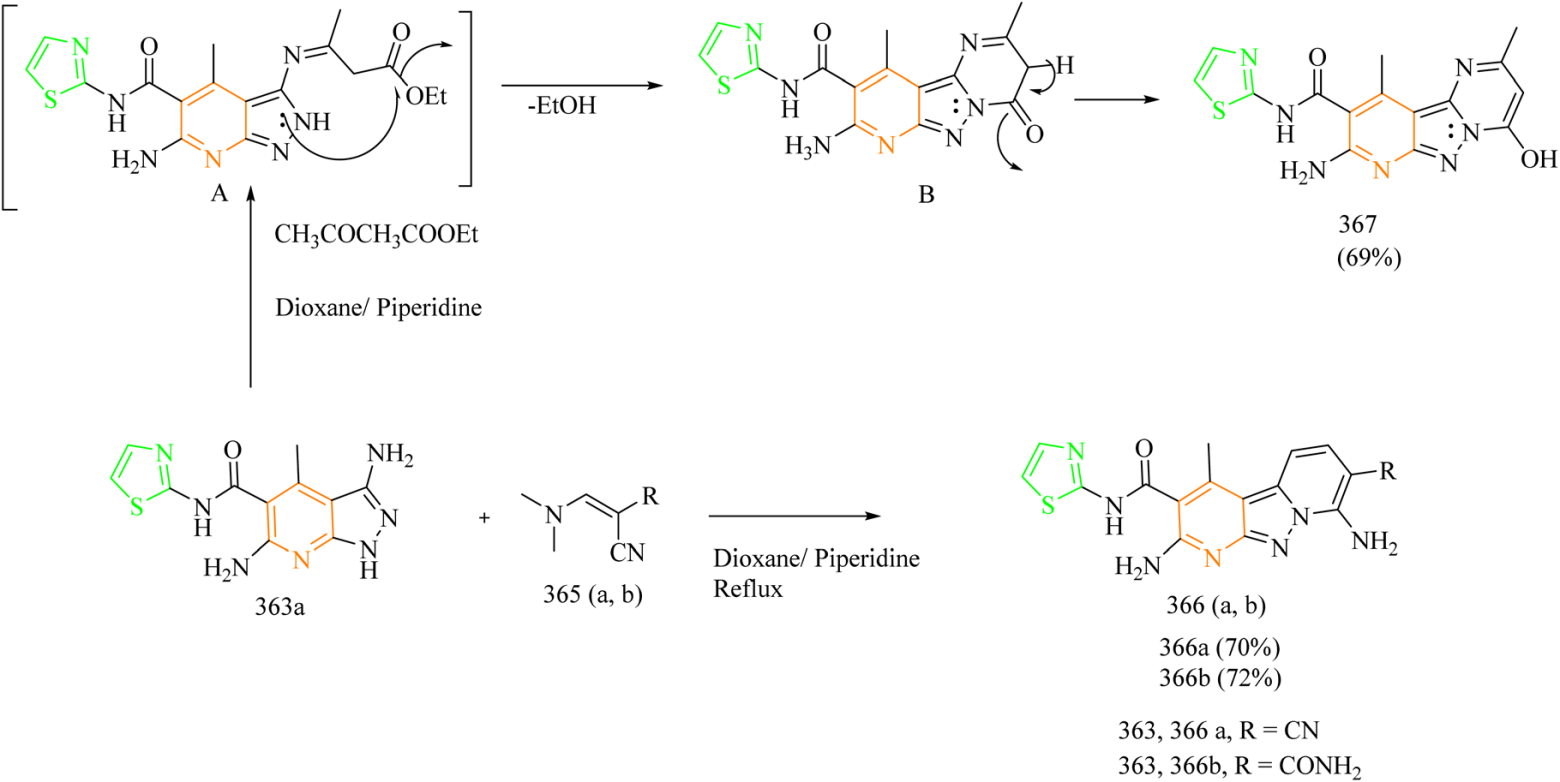
Synthesis of 366(a,b) and 367.

Alamshany and coworkers designed and synthesized pyridine-based and fused pyridine–thiazole hybrid compounds as anticancer agents and inhibitors of EGFR. The pivotal intermediates, pyridinethiones 362(a,b), were prepared *via* a one-pot reaction involving the cyanoacetamide derivative 359, different aldehydes 360(a,b), and 2-cyanoethanethioamide 361 in ethanol under reflux conditions. Subsequently, the intermediate compounds 362(a,b) were reacted with hydrazine hydrate leading to the formation of the target pyrazolo-pyridine-5-carboxamide derivatives 363(a,b) (Scheme 75). The aminopyrazolopyridine derivative 363a was subjected to a cyclocondensation reaction with either malanonitrile 365a or acrylamide 365b. This reaction led to the formation of pyrido-pyrazolecarboxamide derivatives 366a and 366b, respectively. Alternatively, compound 363a was treated with an equimolar amount of ethyl acetoacetate in dioxane resulting in the formation of the corresponding pyrido-pyrazolo- pyrimidine- 9-carboxamide derivative 363 (Scheme 76). Compound 350b demonstrated remarkable cytotoxic potency, exhibiting IC_50_ values of 3.84 µM against HepG-2 and 4.40 µM against MCF-7 cell lines. These results highlight its superior or comparable efficacy to the standard chemotherapeutic agent doxorubicin, which recorded IC_50_ values of 5.78 ± 0.13 µM and 7.32 ± 0.20 µM, respectively. In case of EGFR inhibition, compound 363b demonstrated strong activity, reaching an IC_50_ value of 0.35 ± 0.20 µM, which is close to the potency of reference drug erlotinib (IC_50_ = 0.27 µM). In comparison, compound 363a showed only moderate inhibition with an IC_50_ of 0.61 µM. SAR findings suggested that introducing a pyrazole ring in case of compound 363(a,b) improved greatly improved anticancer activity when compared to the simple pyridine-based compounds 362(a,b). The *p*-methoxyphenyl group in 363b further contributed to its enhanced cytotoxic effects and stronger EGFR inhibition relative to the methyl substituted analogue 363a. Molecular docking supported these results, as 363b achieved a notable binding score of −11.32 kcal mol^−1^ and formed key hydrogen bonds with Met769, Gln767, Thr766, and Asp831 along with an additional interaction between the thiazole sulfur and Ala719. Overall, the 4-methoxyphenyl substituent emerges as an important structural feature that significantly enhances both biological activity and EGFR binding affinity.^82^

The thiazole–pyrimidine hybrids displayed a range of EGFR inhibitory activities, largely influenced by the nature of their substituents. For instance, molecule 311, which carries a 2,5-dioxopyrrolidine ring, fitted more effectively into the hydrophobic regions of the binding pocket. The chromone moiety in molecule 329d strengthened its attachment to Met793 and improved π–π stacking, contributing to its superior potency. In molecule 347, replacing the pyrimidinethione with a compact 6,5-fused system increased the rigidity and electron density of the structure, enabling more stable hydrogen bonds with residues such as Asp800, Asp855 and Lys745. Likewise, molecule 363b showed excellent positioning within the active site where the *p*-methoxyphenyl group supported multiple hydrogen bonds and helped to orient the thiazole sulfur for optimal contact. Overall, incorporating rigid frameworks or electron-rich substituents consistently improved the binding behaviour of these thiazole–pyrimidine hybrids towards EGFR.

## Conclusion

In summary, targeting the EGFR tyrosine kinase domain remains a promising strategy in the battle against cancer, particularly through the development of thiazole-based hybrid molecules. Although thiazole-based hybrids show encouraging *in vitro* activity, several key issues have held them back from advancing toward clinical use and FDA approval. Many compounds still lack strong selectivity for major EGFR mutants like T790M and L858R, reducing their clinical value and increasing the risk of off-target effects. Their potency also tends to be lower than that of existing approved EGFR inhibitors, and they often suffer from poor ADMET properties, including weak metabolic stability and limited solubility. Concerns about toxicity and the limited availability of *in vivo* studies further slow their progress. In addition, the absence of detailed co-crystal structures makes rational optimisation difficult. Together, these factors show that more advanced design strategies and thorough preclinical testing are needed before thiazole hybrids can move closer to clinical translation. Despite existing challenges like drug resistance and genetic mutations, recent advancements in drug discovery have paved the way for designing more selective and effective inhibitors. Continued research in this area holds great potential for improving cancer therapies and overcoming current limitations. Several EGFR inhibitors like avitinib, nazartinib, gefitinib, osimertinib, erlotinib, lazertinib, afatinib, and lapatinib have already been received approval and are known to reduce cell proliferation by blocking protein tyrosine kinase (PTK) activity, thereby dampening downstream signaling pathways. Building on key pharmacophoric features along with insights gained from docking analysis and structure activity relationship (SAR) studies, researchers have been actively investigating a wide range of chemical scaffolds particularly thiazole-based hybrids as promising EGFR-targeting agents.

From the current review, future efforts should focus on designing novel molecules guided by existing SAR data of thiazole analogues. Future research should use advanced structure-based design tools such as improved docking, molecular dynamics simulations, and free-energy calculations to more accurately shape and optimise the thiazole scaffold. At the same time, machine-learning methods, including QSAR models and generative AI, help predict biological activity and suggest new derivatives that align with the SAR trends observed in this study. Techniques like pharmacophore modelling, fragment-based screening, and early ADMET assessment further guide the design of thiazole molecules with greater potency and better drug-like properties. By integrating these computational approaches with experimental validation, the discovery of next-generation thiazole-based inhibitors moves forward in a more targeted and efficient manner.

However, before any new candidates are viable, it is essential to thoroughly evaluate both their short-term and long-term toxicity. Insights gained from SAR studies and molecular docking support more rational design decisions by clarifying which structural features enhance activity and which may contribute to adverse effects. The use of modern AI-based docking tools further strengthens this process by efficiently predicting binding behaviour, identifying potential issues at an early stage, and helping prioritise the most promising molecules. Ultimately, this can pave the way for the design of more effective and safer EGFR inhibitors, enhancing the overall quality of life for patients battling cancer.

## Ethical statement

This study doesn't involve the use of any humans or animals.

## Author contributions

Ancilla Dsouza: writing-original draft, software; V. M. Subrahmanyam: methodology, validation, writing-original draft; Nitinkumar S. Shetty: writing-review & editing, supervision.

## Conflicts of interest

On behalf of the authors, the corresponding author declares no conflicts of interest.

## Data Availability

No primary research results, software or code have been included, and no new data were generated or analysed as part of this review.
